# Integration of mass spectral fingerprinting analysis with precursor ion (MS1) quantification for the characterisation of botanical extracts: application to extracts of *Centella asiatica* (L.) Urban

**DOI:** 10.1002/pca.2936

**Published:** 2020-04-12

**Authors:** Armando Alcazar Magana, Kirsten Wright, Ashish Vaswani, Maya Caruso, Ralph L. Reed, Conner F. Bailey, Thuan Nguyen, Nora E. Gray, Amala Soumyanath, Joseph Quinn, Jan F. Stevens, Claudia S. Maier

**Affiliations:** ^1^ Department of Chemistry Oregon State University Corvallis OR USA; ^2^ Department of Neurology Oregon Health and Science University Portland OR USA; ^3^ Department of Neurology and Parkinson's Disease Research Education and Clinical Care Center (PADRECC), VA Portland Healthcare System Portland OR USA; ^4^ Department of Pharmaceutical Sciences Oregon State University Corvallis OR USA; ^5^ Linus Pauling Institute Oregon State University Corvallis OR USA; ^6^ OHSU‐PSU School of Public Health, Oregon Health & Science University Portland OR USA

**Keywords:** botanicals, *Centella asiatica* (L.) Urb, liquid chromatography, mass spectrometry, precursor (MS1) quantification, Tanimoto 2D structural similarity

## Abstract

**Introduction:**

The phytochemical composition of plant material governs the bioactivity and potential health benefits as well as the outcomes and reproducibility of laboratory studies and clinical trials.

**Objective:**

The objective of this work was to develop an efficient method for the in‐depth characterisation of plant extracts and quantification of marker compounds that can be potentially used for subsequent product integrity studies. *Centella asiatica* (L.) Urb., an Ayurvedic herb with potential applications in enhancing mental health and cognitive function, was used as a case study.

**Methods:**

A quadrupole time‐of‐flight analyser in conjunction with an optimised high‐performance liquid chromatography (HPLC) separation was used for in‐depth untargeted fingerprinting and post‐acquisition precursor ion quantification to determine levels of distinct phytochemicals in various *C. asiatica* extracts.

**Results:**

We demonstrate the utility of this workflow for the characterisation of extracts of *C. asiatica*. This integrated workflow allowed the identification or tentative identification of 117 compounds, chemically interconnected based on Tanimoto chemical similarity, and the accurate quantification of 24 phytochemicals commonly found in *C. asiatica* extracts.

**Conclusion:**

We report a phytochemical analysis method combining liquid chromatography, high resolution mass spectral data acquisition, and post‐acquisition interrogation that allows chemical fingerprints of botanicals to be obtained in conjunction with accurate quantification of distinct phytochemicals. The variability in the composition of specialised metabolites across different *C. asiatica* accessions was substantial, demonstrating that detailed characterisation of plant extracts is a prerequisite for reproducible use in laboratory studies, clinical trials and safe consumption. The methodological approach is generally applicable to other botanical products.

## Abstract 2

Botanical products are popular for promoting resilience, maintaining health and preventing or treating disease. Challenges in defining effects of botanical products reside in achieving batch‐to‐batch reproducibility regarding their composition and product stability, i.e. providing consistent products for clinical trials and consumers. In this work, using *Centella asiatica* (L.) Urb. as a case study, we demonstrate the efficacy of integrating untargeted high‐resolution mass spectrometry with post acquisition precursor ion extraction for determining chemical profiles and quantifying selected marker compounds.

## 1 INTRODUCTION

Plants are extraordinary factories of specialised metabolites, producing more than 200000 distinct compounds across the plant kingdom ^.^ ^1^, ^2^ Phytochemicals play a pivotal role in defence, communication, as well as signalling or regulation of primary metabolic pathways. Their large structural diversity is thought to be due to specific adaptations or functions inherited by evolutionary pressure.^3^

Phytochemicals are the primary source of medicines in many countries.^4^ Their use can reach as much as 80% of people in indigenous populations,^5^ and they are becoming increasingly popular in Western countries.^6^, ^7^, ^8^ Upon ingestion, the dose‐dependent effects of these compounds may have a variety of benefits for human health.^9^ Numerous new reports popularise the use of plant‐derived supplements, including phytochemicals, for promoting human health. The industry is responding to this demand by expanding the diversity of plant‐derived products and dietary supplements. However, concomitantly, concerns regarding the quality, safety and purity of these products have been stimulated by an increasing number of toxicity reports involving plant‐derived supplements.^10^

Botanical supplements are typically complex mixtures of specialised metabolites of which some are biologically active compounds. The phytochemical profiles of botanical preparations can differ significantly from batch to batch since they can be affected by geography, genetics, ontogenetic stage, plant materials used and post‐harvest processing methods among others.^11^, ^12^ The stochastic nature of the phytochemical composition influences the biological and pharmacological activity of the product and thus severely impacts the reproducibility of preclinical studies and clinical trials. Many botanical supplements are plant extracts instead of raw materials. In addition to concerns of adulteration and misidentification of raw materials, the phytochemical profiles in extracts depend highly on the extraction methods used.^13^ Thermal and chemical treatments of plant extracts may cause the degradation of phytochemicals.^14^ During the preparation of botanical supplements, composition and levels of phytochemicals may change, which in turn may affect the bioactivity of the preparations.

Unless disease treatment is claimed, the US Food and Drug Administration (FDA) regulates plant extracts as food; therefore, the requirements to demonstrate safety and efficacy are less stringent compared to pharmaceuticals.^6^ Due to enhanced efforts in improving the characterisation of unregulated over‐the‐counter botanicals, advanced analysis strategies are needed to ascertain authentication and consistency of botanical supplements..^6^, ^10^, ^15^, ^16^, ^17^

In the past, the analysis of phytochemical preparations was largely based on thin layer chromatography (TLC) or liquid chromatography (LC) in conjunction with ultraviolet (UV) spectrophotometric or fluorometric detection. Currently, LC methods in combination with mass spectrometry (MS) are considered state‐of‐the‐art for chemical analysis of plant materials and derived products. Modern high resolving MS platforms offer accurate mass measurements in combination with collision‐induced dissociation techniques for structural analysis and quantification of compounds in phytochemical preparations. Combining high resolution MS with tandem mass spectrometric techniques allows for establishing mass spectral fingerprints of the phytochemical diversity of plant materials and derived products.

In this proof‐of‐concept study, we applied a high resolution MS‐based workflow in conjunction with post acquisition interrogation for the characterisation of aqueous extracts of the medicinal plant *Centella asiatica* (CA), a member of the Apiaceae family, which has been used to improve memory and mental health.^18^, ^19^, ^20^, ^21^ Recent studies in humans and rodent models are supportive of *C. asiatica* preparations as complementary medicine to improve memory in ageing‐related mild cognitive decline and potentially Alzheimer's disease.^21^, ^22^, ^23^ *Centella asiatica* has also been reported to possess other biological activities benefiting human health including anti‐inflammatory and immunostimulant properties, promoting wound healing, and ameliorating leprosy, lupus, tuberculosis and gastric ulcers.^19^, ^21^, ^24^, ^25^, ^26^ The activities of *C. asiatica* have largely been attributed to its constituent triterpenes, saponins and sapogenins.^27^ Almost 60 compounds belonging to these and other phytochemical classes have been reported in *C. asiatica.* ^21^ For many of these, their role in *C. asiatica*'s biological activity or their mode of action is not known, and many others, as yet unidentified compounds are present which may also contribute to its activity. We report here a new phytochemical analysis workflow that allows both untargeted fingerprinting for determining distinct phytochemicals in various *C. asiatica* plant extracts, the tentative identification of 117 of these compounds, and simultaneously the accurate, targeted quantification of eight caffeoylquinic acids, seven flavonoids, five hydroxycinnamic acids and four pentacyclic triterpenoids. We report the analytical method's characteristics including limit of detection (LOD), limit of quantification (LOQ), dynamic range and reproducibility for the quantification method. The integrated workflow was applied to the characterisation of aqueous extracts of *C. asiatica* plant materials from multiple sources.

## 2 EXPERIMENTAL

### 2.1 Chemicals

LC–MS grade methanol and water were purchased from EMD Millipore (Burlington, MA, USA). Formic acid ACS reagent was from Fisher Chemicals (Hampton, NH, USA). The following certified standard compounds were used: 5‐*O*‐caffeoylquinic acid (**1**), epigallocatechin (**2**), catechin (**3**), dihydrocaffeic acid (**4**), 4‐*O*‐caffeoylquinic acid (**5**), 3‐*O*‐caffeoylquinic acid (**6**), caffeic acid (**7**), epicatechin (**8**), 1,5‐dicaffeoylquinic acid (**9**), 1,3‐dicaffeoylquinic acid (**10**), rutin (**11**), dihydroferulic acid (**12**), 3,4‐dicaffeoylquinic acid (**13**), 3,5‐dicaffeoylquinic acid (**14**), ferulic acid (**15**), 4,5‐dicaffeoylquinic acid (**16**), naringin (**17**), isoferulic acid (**18**), quercetin (**19**), madecassoside (**20**), asiaticoside (**21**), kaempferol (**22**), madecassic acid (**23**) and asiatic acid (**24**). Compounds **2**, **3**, **6**, **12**, **15** and **18** were from Sigma Aldrich (St Louis, MO, USA); **1**, **11**, **17** and **19** were from TCI America (Portland, OR, USA); **13** and **16** were from ChromaDex (Irvine, CA, USA) and the remaining compounds were from Toronto Research Chemicals (Canada).

Caffeoylquinic acids are prone to degradation or isomerisation under certain conditions including pH, light exposure, and temperature.^28^, ^29^, ^30^ To protect compounds from degradation, all standards and samples were prepared in methanolic solutions containing 0.1% *v*/*v* formic acid and kept in the dark at −20°C until analysis.

### 2.2 Plant materials and preparation of aqueous extracts of *Centella asiatica*

The identity of the plant materials was confirmed at the supplier (Oregon's Wild Harvest) by organoleptic analysis and Fourier‐transform infrared analysis (FTIR). Identity was further verified at Oregon Health and Science University by thin‐layer chromatographic comparison of zone profiles with earlier batches of *C. asiatica*, and with reference standards of characteristic triterpenes (asiatic acid, madecassic acid, asiaticoside, madecassoside) as well as caffeoylquinic acids known to be found in *C. asiatica*. Voucher samples of all plant materials have been deposited in the Herbarium at Oregon State University. Voucher numbers for plant materials from which extracts were derived are CA1 (OSC‐V‐258635), CA2 (OSC‐V‐258632), CA3 (OSC‐V‐258631), CA4 (OSC‐V‐258629), CA5 (OSC‐V‐258627), CA6 (OSC‐V‐258630), CA7 (OSC‐V‐258634), and CA8 (OSC‐V‐258633).^23^ The preparation of the *C. asiatica* water extracts was reported previously.^23^, ^31^ In brief, dried *C. asiatica* extracts were prepared by refluxing aerial parts of the plant (stems, leaves and flowers but not roots; 80 grams per 1 litre of deionised water) for 1.5 h, cooling for 30 min to allow for handling, and then filtering the suspension to remove plant debris. The aqueous extracts were freeze‐dried and stored at −20°C and analysed within 2 months of preparation. This method was modified from earlier work by Veerendra *et al*.^32^, ^33^ who showed that exhaustive water extraction of *C. asiatica* produced a residue with greater cognitive enhancing properties than extracts made with methanol or chloroform.

For quantification of the individual compounds in dried *C. asiatica* extracts, a stock solution was prepared as follows. Briefly, 10 mg of each freeze‐dried extract powder was resuspended in 10 mL of aqueous methanol (70% *v*/*v* with 0.1% *v/v* of formic acid) by sonication (30 min, 25°C; see Supporting Information Figure S9), centrifuged (14000 × *g* for 10 min) and filtered with 0.22‐μm polyvinylidene fluoride (PVDF) Whatman filters before analysis. This procedure was used to prepare extracts from eight different accessions of the plant materials labelled from CA1 to CA8. Aliquots of 1 mL from each extract were pooled to generate a quality control sample (QC) used for evaluating LC–MS/MS platform performance.

### 2.3 Fingerprinting of *Centella asiatica* extracts by untargeted data‐dependent analysis

For the chemical profiling analyses, a pooled CA sample (QC sample) was used. Untargeted high‐performance liquid chromatography (HPLC) combined with high resolution accurate LC–MS/MS was conducted using a Shimadzu Nexera UHPLC system connected to an AB SCIEX TripleTOF® 5600 mass spectrometer equipped with a Turbo V ionisation source operated in the electrospray ionisation (ESI) mode.

Chromatographic separation was achieved using an Inertsil Phenyl‐3 column (4.6 mm × 150 mm, 100 Å, 5 μm; GL Sciences, Torrance, CA, USA). The injection volume was 10 μL. Three technical replicates were conducted. Gradient elution was performed using a mobile phase consisting of solvent A, water containing 0.1% *v*/*v* formic acid, and solvent B, methanol containing 0.1% *v/v* formic acid. Flow rate was 0.4 mL/min. The chromatographic method was 30 min, and the gradient design was as follows: an initial 1 min at 5% B, followed by 5 to 30% B from 1 to 10 min, then 30 to 100% B from 10 to 20 min, hold at 100% B from 20 to 25 min, and then return to 5% B from 25 to 30 min.

Data‐dependent acquisitions (DDAs) were conducted for obtaining precursor and fragment ion information for aiding in annotating compounds in the CA extracts.^34^ DDA analyses were conducted using the negative (ESI−) and positive ionisation (ESI+) mode. For detecting negative ions, the following parameter settings were used to operate the mass spectrometer: spray voltage −4200 V; source temperature 550°C and a period cycle time of 950 ms was used. The following settings were used: full scan with ion accumulation of 150 ms, followed by a dynamic MS/MS selection of the eight most intense ions with 100 ms accumulation; after two MS/MS acquisitions the precursor (fragmented) ions were excluded for 30 s; collision energy 35 V with collision energy spread (CES) of 15 V ramped through each MS/MS scan using a range of *m/z* 100–1200. For ESI+ acquisitions, the instrument settings were the same as used in the negative ion mode except that the spray voltage was set to 4500 V. The mass spectrometer was equipped with a calibrant delivery system. Mass calibration was automatically performed after every fifth LC run.

### 2.4 Data processing and annotation of plant metabolites

Annotation confidence was established according to reporting criteria for chemical analysis suggested by the Metabolomics Standards Initiative.^35^, ^36^ For Level 1 (L1) annotations (Tables 1 and 2) we used accurate mass, fragment ion spectral similarity and retention time (RT) co‐elution based on an authentic commercially available standards. Standard addition experiments were also conducted as part of developing the quantitative method (see later).

In Figure S1 the workflow is described to obtain in putative annotations [Level 2 (L2) annotations^35^, ^36^] based on exact mass, isotopic pattern and MS/MS spectral data. In addition, three manual data evaluations were included: (1) examination of the metabolite structures with respect to the suitability of the ionisation mode in which a compound was detected (i.e. basic sites in a molecule that can be protonated in ESI+ or labile protons for ESI−), (2) elution peaks for tentatively annotated features were interrogated to omit compounds originating from in‐source fragmentation, and (3) only compounds previously reported in plants were kept and listed in Table 1. It is important to note, that these compounds are tentative annotations only in accordance to the guidelines described by Sumner *et al*.^35^ and will need to be further confirmed in future work.

**TABLE 1:** Summary of detected compounds in *Centella asiatica* extracts (pooled sample) by extensive querying and comparison with spectral data (METLIN, HMDB, ChEBI and our in‐house library) and compound libraries (KNApSAcK and PantMAT) using Progenesis QI™ and applying the workflow shown in Figure S1. Compounds are labelled with their respective PubChem CID. Additional parameters are shown in Table S1. Categories were assigned according to structural similarity using Tanimoto algorithm, and they may correspond to more than one compound class. Compounds confirmed using authentic standards are shown in bold [Level 1 (L1) identifications]; all other compound assignments are based on Level 2 annotations (MS/MS spectral matches are presented in Figure S7). Eighty‐seven compounds that were detected for the first time in *C. asiatica* extracts are denoted with an asterisk ‘*’

Compound	CID	InChIKey	Molecular formula
*Amino acid derivatives*			
2‐Pyrrolidone‐5‐carboxylic acid	499	ODHCTXKNWHHXJC‐UHFFFAOYSA‐N	C_5_H_7_NO_3_
1‐beta‐d‐Glucopyranosyl‐l‐tryptophan*	11772967	ZHBHZDMTVVJASV‐JOSVURMMSA‐N	C_17_H_22_N_2_O_7_
2,6‐Piperidinedicarboxylic acid*	557515	SOOPBZRXJMNXTF‐UHFFFAOYSA‐N	C_7_H_11_NO_4_
4‐Guanidinobutanoic acid*	25200642	TUHVEAJXIMEOSA‐UHFFFAOYSA‐N	C_5_H_11_N_3_O_2_
5‐Methoxy‐l‐tryptophan*	151018	KVNPSKDDJARYKK‐JTQLQIEISA‐N	C_12_H_14_N_2_O_3_
6‐amino‐9H‐purine‐9‐propanoic acid*	255450	QXAYJKFBMWMARF‐UHFFFAOYSA‐N	C_8_H_9_N_5_O_2_
6‐Oxo‐2‐piperidinecarboxylic acid*	3014237	FZXCPFJMYOQZCA‐UHFFFAOYSA‐N	C_6_H_9_NO_3_
l‐Arginine*	28782	ODKSFYDXXFIFQN‐BYPYZUCNSA‐N	C_6_H_14_N_4_O_2_
*N*‐(1‐Deoxy‐1‐fructosyl)phenylalanine*	101039148	FAVRCIXPIVJIPN‐VJDSNFAGSA‐N	C_15_H_21_NO_7_
*N*‐Acetyl‐l‐glutamic acid*	70914	RFMMMVDNIPUKGG‐YFKPBYRVSA‐N	C_7_H_11_NO_5_
Niacin (nicotinic acid)*	938	PVNIIMVLHYAWGP‐UHFFFAOYSA‐N	C_6_H_5_NO_2_
Pantothenic acid	6613	GHOKWGTUZJEAQD‐ZETCQYMHSA‐N	C_9_H_17_NO_5_
Succinyl‐l‐proline*	194156	NEBOPDYAXPDYHQ‐LURJTMIESA‐N	C_9_H_13_NO_5_
Vincosamide*	10163855	LBRPLJCNRZUXLS‐AZVRXDBZSA‐N	C_26_H_30_N_2_O_8_
*Amino sugar derivatives*			
Enicoflavine*	5281564	GBJQPSBGSKNYHV‐YVMONPNESA‐N	C_10_H_13_NO_4_
Muramic acid*	433580	MSFSPUZXLOGKHJ‐UHFFFAOYSA‐N	C_9_H_17_NO_7_
*N*‐Acetyl‐d‐glucosamine*	899	OVRNDRQMDRJTHS‐UHFFFAOYSA‐N	C_8_H_15_NO_6_
Soyacerebroside I*	131751281	HOMYIYLRRDTKAA‐UYIJODJPSA‐N	C_40_H_75_NO_9_
d‐1‐[(3‐Carboxypropyl)amino]‐1‐deoxyfructose*	131752417	HUEOABWGBTXQNF‐SFKDOBOXSA‐N	C_10_H_19_NO_7_
*Choline derivatives*			
Betaine*	247	KWIUHFFTVRNATP‐UHFFFAOYSA‐N	C_5_H_11_NO_2_
Choline*	305	OEYIOHPDSNJKLS‐UHFFFAOYSA‐N	C_5_H_14_NO^+^
Choline *O*‐sulphate*	486	WXCQAWGXWVRCGP‐UHFFFAOYSA‐O	C_5_H_14_NO_4_S^+^
Phosphocholine	1014	YHHSONZFOIEMCP‐UHFFFAOYSA‐O	C_5_H_15_NO_4_P^+^
*Di‐caffeoylquinic acids*			
**1,3‐Di‐caffeoylquinic acid**	6474640	YDDUMTOHNYZQPO‐PSEXTPKNSA‐N	C_25_H_24_O_12_
1,4‐Di‐caffeoylquinic acid*	12358846	IYXQRCXQQWUFQV‐RDJMKVHDSA‐N	C_25_H_24_O_12_
**1,5‐Di‐caffeoylquinic acid**	5281769	YDDUMTOHNYZQPO‐RVXRWRFUSA‐N	C_25_H_24_O_12_
**3,4‐Di‐caffeoylquinic acid**	5281780	UFCLZKMFXSILNL‐PSEXTPKNSA‐N	C_25_H_24_O_12_
**3,5‐Di‐caffeoylquinic acid**	6474310	KRZBCHWVBQOTNZ‐RDJMKVHDSA‐N	C_25_H_24_O_12_
**4,5‐Di‐caffeoylquinic acid**	6474309	UFCLZKMFXSILNL‐RVXRWRFUSA‐N	C_25_H_24_O_12_
*Fatty acid derivatives*			
Caprylic acid*	379	WWZKQHOCKIZLMA‐UHFFFAOYSA‐N	C_8_H_16_O_2_
Palmitic acid*	985	IPCSVZSSVZVIGE‐UHFFFAOYSA‐N	C_16_H_32_O_2_
Tetradecanedioic acid*	13185	HQHCYKULIHKCEB‐UHFFFAOYSA‐N	C_14_H_26_O_4_
16‐Hydroxypalmitic acid*	10466	UGAGPNKCDRTDHP‐UHFFFAOYSA‐N	C_16_H_32_O_3_
Traumatic acid*	5283028	MAZWDMBCPDUFDJ‐VQHVLOKHSA‐N	C_12_H_20_O_4_
12‐Oxodihydrophytodienoic acid*	5716902	BZXZFDKIRZBJEP‐GTOOTHNYSA‐N	C_18_H_30_O_3_
Nomilinic acid 17‐glucoside*	444212	MUZNNCNJBAPYJF‐UNWTYGGYSA‐N	C_34_H_48_O_16_
*Flavonoids*			
3,5‐Dihydroxyphenyl 1‐*O*‐(6‐*O*‐galloyl‐β‐d‐glucopyranoside)	131752603	WVHDGXKUZIDTIN‐UHFFFAOYSA‐N	C_19_H_20_O_12_
6‐C‐α‐l‐Arabinosyl‐8‐C‐β‐l‐arabinosylapigenin	122391238	LDVNKZYMYPZDAI‐DKEQBBAASA‐N	C_25_H_26_O_13_
Apimaysin*	101920411	LCQVQAZLYBJMGJ‐YWOWJUDASA‐N	C_27_H_28_O_13_
Astragalin	5282102	JPUKWEQWGBDDQB‐QSOFNFLRSA‐N	C_21_H_20_O_11_
**Kaempferol**	5280863	IYRMWMYZSQPJKC‐UHFFFAOYSA‐N	C_15_H_10_O_6_
Mangiferin*	5281647	AEDDIBAIWPIIBD‐ZJKJAXBQSA‐N	C_19_H_18_O_11_
**Naringin**	442428	DFPMSGMNTNDNHN‐ZPHOTFPESA‐N	C_27_H_32_O_14_
Pelargonidin 3‐*O*‐glucoside*	443648	ABVCUBUIXWJYSE‐GQUPQBGVSA‐O	C_21_H_21_O_10_+
**Quercetin**	5280343	REFJWTPEDVJJIY‐UHFFFAOYSA‐N	C_15_H_10_O_7_
Quercetin 3‐(6′‐acetylglucoside)*	44259187	IGLUNMMNDNWZOA‐CHUITGBKSA‐N	C_23_H_22_O_13_
Quercetin 3‐*O*‐glucoside*	5280804	OVSQVDMCBVZWGM‐QSOFNFLRSA‐N	C_21_H_20_O_12_
**Rutin**	5280805	IKGXIBQEEMLURG‐NVPNHPEKSA‐N	C_27_H_30_O_16_
Glabraoside A*	102393599	OKKMHZTVGJIQAP‐FVELTCQYSA‐N	C_30_H_30_O_13_
*Hexoside derivatives*			
Stachyose*	439531	UQZIYBXSHAGNOE‐XNSRJBNMSA‐N	C_24_H_42_O_21_
Carlosic acid methyl ester*	122391261	MQWPWZMHPWXSGL‐ZETCQYMHSA‐N	C_11_H_14_O_6_
Daucic acid*	5316316	KUKCUROTFRBUNU‐UHFFFAOYSA‐N	C_7_H_8_O_7_
Digalacturonate*	439694	IGSYEZFZPOZFNC‐LKIWRGPLSA‐N	C_12_H_18_O_13_
Dihydroactinidiolide*	27209	IMKHDCBNRDRUEB‐UHFFFAOYSA‐N	C_11_H_16_O_2_
Furaneol 4‐(6‐malonylglucoside)*	131750900	QJYOBEMAMLWZTF‐UHFFFAOYSA‐N	C_15_H_20_O_11_
Isovalerylglucuronide*	137383	VOJAALAAOYUSCT‐ZCLKDUABSA‐N	C_11_H_18_O_8_
Linustatin*	119301	FERSMFQBWVBKQK‐CXTTVELOSA‐N	C_16_H_27_NO_11_
Purgic acid B*	16091605	YTQXXUYELDKIKL‐YLGYQDNRSA‐N	C_52_H_92_O_29_
*Hydroxycinnamic acids*			
**Caffeic acid** ^**a**^	689043	QAIPRVGONGVQAS‐DUXPYHPUSA‐N	C_9_H_8_O_4_
**Isoferulic acid** *	736186	QURCVMIEKCOAJU‐HWKANZROSA‐N	C_10_H_10_O_4_
**Dihydrocaffeic acid** *	348154	DZAUWHJDUNRCTF‐UHFFFAOYSA‐N	C_9_H_10_O_4_
**Dihydroferulic acid** *	14340	BOLQJTPHPSDZHR‐UHFFFAOYSA‐N	C_10_H_12_O_4_
**Ferulic acid**	445858	KSEBMYQBYZTDHS‐HWKANZROSA‐N	C_10_H_10_O_4_
*Monocaffeoylquinic acids*			
**3‐*O*‐Caffeoylquinic acid**	1794427	CWVRJTMFETXNAD‐JUHZACGLSA‐N	C_16_H_18_O_9_
**4‐*O*‐Caffeoylquinic acid**	9798666	GYFFKZTYYAFCTR‐AVXJPILUSA‐N	C_16_H_18_O_9_
**5‐*O*‐Caffeoylquinic acid**	5280633	CWVRJTMFETXNAD‐NXLLHMKUSA‐N	C_16_H_18_O_9_
*Organic acids*			
Citric acid*	19782904	KRKNYBCHXYNGOX‐UHFFFAOYSA‐N	C_6_H_8_O_7_
l‐Ribulose*	644111	ZAQJHHRNXZUBTE‐UCORVYFPSA‐N	C_5_H_10_O_5_
Malate*	20130941	BJEPYKJPYRNKOW‐UHFFFAOYSA‐N	C_4_H_6_O_5_
Succinate*	1110	KDYFGRWQOYBRFD‐UHFFFAOYSA‐N	C_4_H_6_O_4_
*Phenolic compounds*			
3‐Hydroxy‐2‐oxo‐3‐phenylpropanoic acid	71581094	ZHLWCBHWYUISFY‐ZETCQYMHSA‐N	C_9_H_8_O_4_
1‐Caffeoyl‐5‐feruloylquinic acid*	121225501	DJXURFUTIYZESV‐BQYLRUKMSA‐N	C_26_H_26_O_12_
3,4‐Dihydroxybenzaldehyde*	8768	IBGBGRVKPALMCQ‐UHFFFAOYSA‐N	C_7_H_6_O_3_
3,5‐Dihydroxy‐2‐methylphenyl beta‐d‐glucopyranoside*	46184089	AXTQBXDFUAAFPD‐UJPOAAIJSA‐N	C_13_H_18_O_8_
3‐Hydroxycoumarin	13650	MJKVTPMWOKAVMS‐UHFFFAOYSA‐N	C_9_H_6_O_3_
4‐Hydroxybenzaldehyde*	126	RGHHSNMVTDWUBI‐UHFFFAOYSA‐N	C_7_H_6_O_2_
5‐Methoxysalicylic acid*	75787	IZZIWIAOVZOBLF‐UHFFFAOYSA‐N	C_8_H_8_O_4_
8‐Acetoxy‐4′‐methoxypinoresinol 4‐glucoside*	73830447	ZKCRENDTQNGLGO‐UHFFFAOYSA‐N	C_29_H_36_O_13_
Aesculin*	5281417	XHCADAYNFIFUHF‐TVKJYDDYSA‐N	C_15_H_16_O_9_
**Catechin**	9064	PFTAWBLQPZVEMU‐DZGCQCFKSA‐N	C_15_H_14_O_6_
Coumarin	323	ZYGHJZDHTFUPRJ‐UHFFFAOYSA‐N	C_9_H_6_O_2_
**Epicatechin**	72276	PFTAWBLQPZVEMU‐UKRRQHHQSA‐N	C_15_H_14_O_6_
**Epigallocatechin** *	72277	XMOCLSLCDHWDHP‐IUODEOHRSA‐N	C_15_H_14_O_7_
Folinic acid*	6006	VVIAGPKUTFNRDU‐ABLWVSNPSA‐N	C_20_H_23_N_7_O_7_
Ginkgoic acid*	5281858	YXHVCZZLWZYHSA‐FPLPWBNLSA‐N	C_22_H_34_O_3_
Kuwanon Y*	14334307	YYUHPJKWIHNMSV‐UJOAHLBMSA‐N	C_34_H_30_O_9_
Kynurenic acid*	3845	HCZHHEIFKROPDY‐UHFFFAOYSA‐N	C_10_H_7_NO_3_
N1,N5,N10,N14‐tetra‐*trans*‐*p*‐Coumaroylspermine*	9810941	KKJYIHSXTUGJLP‐BRJCPHQQSA‐N	C_46_H_50_N_4_O_8_
Phlorin*	476785	WXTPOHDTGNYFSB‐RMPHRYRLSA‐N	C_12_H_16_O_8_
Tropic acid*	10726	JACRWUWPXAESPB‐UHFFFAOYSA‐N	C_9_H_10_O_3_
Xanthurenic acid*	5699	FBZONXHGGPHHIY‐UHFFFAOYSA‐N	C_10_H_7_NO_4_
*Purine derivatives*			
2′‐*O*‐Methyladenosine*	102213	FPUGCISOLXNPPC‐IOSLPCCCSA‐N	C_11_H_15_N_5_O_4_
Adenine*	190	GFFGJBXGBJISGV‐UHFFFAOYSA‐N	C_5_H_5_N_5_
Adenosine*	60961	OIRDTQYFTABQOQ‐KQYNXXCUSA‐N	C_10_H_13_N_5_O_4_
cAMP*	6076	IVOMOUWHDPKRLL‐KQYNXXCUSA‐N	C_10_H_12_N_5_O_6_P
5′‐Deoxy‐5′‐(methylsulfinyl)adenosine*	165114	WXOJULRVRHWMGT‐JLQSGANNSA‐N	C_11_H_15_N_5_O_4_S
Succinoadenosine*	126969142	VKGZCEJTCKHMRL‐PRWBTVLESA‐N	C_14_H_17_N_5_O_8_
Guanosine*	6802	NYHBQMYGNKIUIF‐UUOKFMHZSA‐N	C_10_H_13_N_5_O_5_
*Terpenoids*			
26‐(2‐Glucosyl‐6‐acetylglucosyl]‐1,3,11,22‐tetrahydroxyergosta‐5,24‐dien‐26‐oate*	131752817	CRRPFFTZRFACDM‐ZCXUNETKSA‐N	C_42_H_66_O_17_
**Asiatic acid**	119034	JXSVIVRDWWRQRT‐UYDOISQJSA‐N	C_30_H_48_O_5_
**Asiaticoside**	24721205	WYQVAPGDARQUBT‐HJCNVAKJSA‐N	C_48_H_78_O_19_
Dysolenticin B*	56601655	RPPAVMFODBKIDO‐HALKBXBMSA‐N	C_30_H_42_O_3_
Gentiopicroside*	88708	DUAGQYUORDTXOR‐GPQRQXLASA‐N	C_16_H_20_O_9_
**Madecassic acid**	73412	PRAUVHZJPXOEIF‐AOLYGAPISA‐N	C_30_H_48_O_6_
**Madecassoside**	91885295	BNMGUJRJUUDLHW‐RSQPUIDYSA‐N	C_48_H_78_O_20_
Sambacin*	131752486	SIVWXOPASOMQQC‐PANBKRDHSA‐N	C_26_H_36_O_12_
Swertiamarin*	442435	HEYZWPRKKUGDCR‐QBXMEVCASA‐N	C_16_H_22_O_10_
Tsangane L 3‐glucoside*	73981648	UJRMJTIXXKZFGB‐UHFFFAOYSA‐N	C_19_H_34_O_7_
b‐Chlorogenin 3‐[4′‐(2′‐glucosyl‐3′‐xylosylglucosyl)galactoside]	74193143	WRRAISMCUAHXHF‐UHFFFAOYSA‐N	C_50_H_82_O_23_
Shanzhiside*	11948668	YSIFYNVXJOGADM‐KDYWOABDSA‐N	C_16_H_24_O_11_
*Others*			
Cytosine*	597	OPTASPLRGRRNAP‐UHFFFAOYSA‐N	C_4_H_5_N_3_O
Longicamphenylone*	91747202	VMYWIJUHQAMXNC‐UHFFFAOYSA‐N	C_15_H_24_O
Longifolenaldehyde*	565584	PBMHTGOFWRRJFS‐UHFFFAOYSA‐N	C_15_H_24_O
Uric acid*	1175	LEHOTFFKMJEONL‐UHFFFAOYSA‐N	C_5_H_4_N_4_O_3_
6‐Docosenamide*	44584605	COUPDYRANTUKKV‐MSUUIHNZSA‐N	C_22_H_43_NO
Deoxyfructosazine*	73452	FBDICDJCXVZLIP‐VSSNEEPJSA‐N	C_12_H_20_N_2_O_7_
Ginsenoyne K*	15736266	SYNBBWLEYQBFQT‐NTCAYCPXSA‐N	C_17_H_24_O_3_

Raw data processing was performed using Progenesis QI™ software with METLIN™ plugin V1.0.6499.51447 (NonLinear Dynamics, Newcastle Upon Tyne, UK) and entailed peak picking, alignment and searching of multiple databases to assist in compound annotations. For the current study, we searched the mass spectral data against METLIN,^37^ Human Metabolome Database (HMDB),^38^ Chemical Entities of Biological Interest (ChEBI)^39^ (online versions, April 2018) and an in‐house compound library based on the Mass Spectrometry Metabolite Library of Standards (MSMLS) consisting of 619 standards (IROA Technologies, Bolton, MA, USA) and other commercially available standards including the 24 compounds used as phytochemical markers in this study (650 total).^40^ Progenesis QI™ uses as built‐in search engine Metascope and provides a “score” for the quality of the compound annotation, using a range from 0 to 100, with 100 being a perfect match based on the mean of multiple similarity metrics.^41^ The current data were evaluated based on the accurate mass similarity, isotope similarity, and fragmentation score (ranging from 0 to 60 representing how well the observed data matches the spectral library entries or the theoretical fragment data based on the bond dissociation approach which is a computational method that calculates expected fragments based on theoretically derived bond dissociation energies^42^). Progenesis QI's fragmentation algorithm is described by Wolf *et al*.^42^ and Horai *et al*.^43^ A Progenesis QI score ≥ 50 is typically reached when isotopic pattern similarity is above 90%, MS/MS spectral data similarity is > 50% and the deviation of the accurate mass from the exact mass is lower than 5 ppm. Progenesis QI score ≥ 50 was considered as adequate for being considered as a candidate for putative annotation (L2 annotations according to Sumner *et al*.^35^). This score is more rigorous than previous reports using Progenesis QI™ with a score > 31.6,^44^ putative annotations based only on accurate mass and isotope similarity^45^ or with a mass error of 20 ppm.^46^ Additional features were assigned by querying and comparison with KNApSAcK online library.^47^ Supporting Information Table S1 lists identified (L1) and putatively assigned (but unverified) metabolites (L2 annotations), and provides access to the following properties: RT, monoisotopic ion mass, ions observed and molecular formula. Figure S7 compiles positive matches (red lines) with the entries in the respective spectral libraries. In the case of structural isomers, the best match (highest score) against the MS/MS spectral data was selected. To illustrate chromatographic performance, we used extracted ion chromatograms (XICs) of 22 annotated ions detected in the positive ion mode (Figure S2) and 24 detected using the negative ion mode (Figure S3).

### 2.5 Chemical similarity network and clustering

We built a chemical space network based on the compounds listed in Tables 1 and S1. In this network, terminal nodes represent compounds and edges (branches) identify similarity relationships based on two‐dimensional (2D) chemical structures. We used the Tanimoto algorithm, T(A,B) = A∩B/A∪B, often referred to as intersection over union, with A and B representing structures of molecule A and molecule B, for calculating measures of similarity. For this purpose, we used PubChem Score Matrix Service V1.3 according to Sunghwan Kim 2016.^48^ The PubChem server uses the simplified molecular input line‐entry system (SMILES) identifiers to compute Tanimoto coefficients and then creates edges between similar structures if the coefficients are greater than or equal to the set threshold value (0.68). The derived Tanimoto coefficient represents an associative coefficient with a value ranging from 0 to 1, numerically expressing the structural similarity between a 2D binary comparison (0 being no similarity and 1 being complete similarity).^49^, ^50^ A Tanimoto coefficient greater or equal to 0.68 indicates that the compounds being compared are structurally similar and statistically significant at the 95% confidence interval.^51^ Tanimoto coefficients were exported to Cytoscape (V3.6.1) for graphic visualisation and a 2D structural similarity network was created for the compounds listed in Table 1.

### 2.6 Method development for quantification of selected phytochemicals in extracts

A quantification method was developed for 24 compounds (Figure S4). The method uses the same chromatographic conditions as described in the untargeted analysis section and the mass spectrometer was operated in ESI− mode using the following settings: spray voltage −4200 V; source temperature 550°C; period cycle time 950 ms; precursor ions accumulation time 100 ms; scan range *m/z* 100–1200 (as described earlier).

External calibration curves were acquired for the 24 authentic compounds based on using the area under the curve of the precursor ion (MS1‐based). Solutions containing analytical blanks, 0.005, 0.01, 0.05, 0.10, 0.50, 1.00, 5 and 10 mg/L of all compounds were prepared in 70% *v*/*v* methanol containing 0.1% *v/v* of formic acid. For quantification, SCIEX MultiQuant™ V3.0.2 analysis software was used, calculating the peak areas under the curve for precursor ions.

#### 2.6.1 Accuracy and recovery experiments

To test the accuracy of the method using precursor ions, three standard mixtures of known concentrations (low, 0.05 mg/L; medium, 0.50 mg/L; high, 5.00 mg/L) were evaluated. Standard addition of authentic standards was performed, and recovery experiments were conducted for CA extracts using precursor ions. Quality control samples were spiked with the 24 available standards at two different concentration levels (0.25 ng and 5 ng on‐column for each compound). Thus, 1 mL of standard mix containing 0.0, 0.05 or 1.0 mg/L of each authentic compound was added separately to 1.0 mL of the pooled sample (200 mg dried CA powder/L).

#### 2.6.2 Application of precursor ion (MS1) quantification method for plant extracts

For precursor ion quantification (MS1 quantification) of phytochemicals in extracts, the same chromatographic runs for untargeted analysis were used for quantification of distinct phytochemicals.

## 3 RESULTS AND DISCUSSION

### 3.1 Untargeted fingerprinting analysis of CA extracts

We developed a chromatographic method for chemical profiling of botanical samples that uses a phenyl‐bonded phase, whereby the phenyl groups are directly bonded to the silica surface and separations are governed by π–π interactions. This stationary phase was selected to take advantage of the presence of phenolic scaffolds in many specialised metabolites, in particular those originating from the phenylpropanoid biosynthesis pathway. The method requires 30 min per chromatographic run. This LC separation method provides suitable resolution and peak capacity for chemical fingerprinting based on DDAs, while minimising peak suppression and matrix effects. Representative XICs are shown in Figures S2 and S3. The peak width at half height is sufficient to conduct quantification of phenolic compounds using precursor ion (MS1) quantification with sufficient reproducibility of peak area determinations.

**FIGURE 1:**
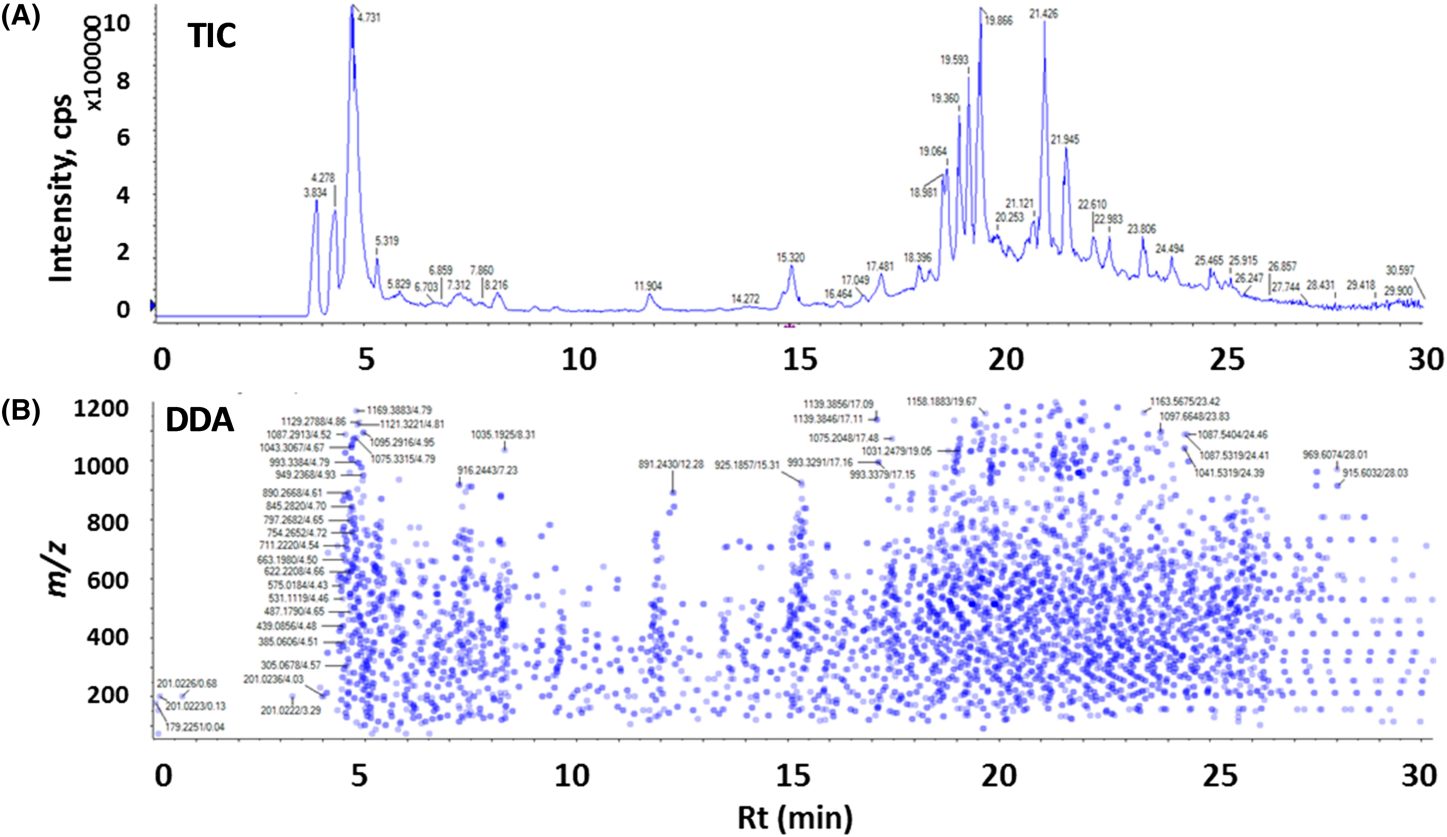
Examples of typical data obtained by untargeted analysis of a pooled *Centella asiatica* (CA) water extract using the data dependent acquisition mode. (A) Total ion chromatogram (ESI−) (10 μL injection, 1 mg/L). (B) Distribution map of precursor ions submitted to collision induced dissociation along the elution period. The *y*‐axis provides *m/z* information for the precursor ion; the *x*‐axis represents the elution times for each precursor ion. Each one of the 5512 dots contains a fragmentation spectrum. The intensity of the colour blue represents the ion abundance of the precursor ion. TIC, total ion chromatogram; DDA, data dependent acquisition. [Colour figure can be viewed at wileyonlinelibrary.com]

Figure 1 shows a typical total ion chromatogram (TIC) for a *C. asiatica* water extract and the most intense molecular features fragmented in the DDA experiment acquired in negative ion mode. From over 20000 *m/z*‐features detected, 117 compounds were annotated (Table 1) after applying the workflow outlined in Figure S1. To our knowledge, this analysis includes 87 compounds that have been reported in plants previously but have been now detected for the first time in *C. asiatica* extracts.^17^, ^19^, ^21^, ^24^, ^25^, ^27^, ^52^, ^53^, ^54^, ^55^, ^56^, ^57^, ^58^, ^59^ MS/MS spectra and spectral matches of the newly detected compounds in CA are provided in Figure S7. Some of the most abundant compounds include six di‐caffeoylquinic acid isomers, quinic acid, mono‐caffeoylquinic acids, and several glycosides, such as asiaticoside, madecassoside and quercetin 3‐*O*‐glucoside. It is noteworthy that the current chromatographic separation conditions resolved di‐caffeoylquinic acids isomers 3,4‐, 3,5‐ and 4,5‐di‐caffeoylquinic acids (Table S1, Figure S5). Analytical parameters for the annotated compounds, namely *m/z*, RT, detected adducts and molecular formulas are shown in detail in Table S1. When compounds were detected in both ion modes, the one with the highest signal‐to‐noise (*S*/*N*) ratio was included. Annotated compounds include five hydroxycinnamic acids, nine mono‐ and di‐caffeoylquinic acids, 12 terpenoids, 13 flavonoids, 11 hexosides among other phytochemicals (Table 1).

**FIGURE 2:**
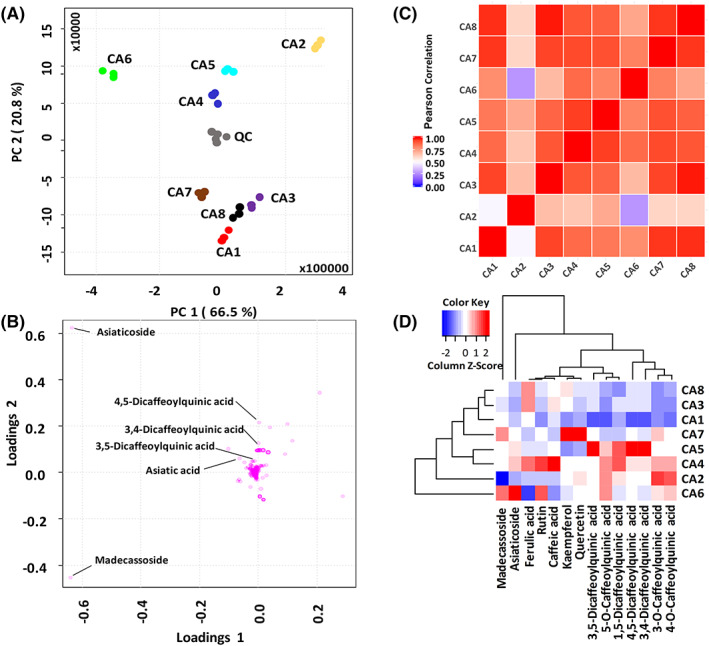
Analysis of similarities and dissimilarities of *Centella asiatica* (CA) water extracts. **(**A) Scores plot, each set of dots (technical triplicates) represents a CA extract from eight different accessions (CA1–CA 8). (B) Loadings plot indicating 11 selected compounds with higher concentration across quantified phytochemicals. Di‐caffeoylquinic acids and triterpenes change across accessions. Principal component analysis (PCA) was performed using 5512 *m/z* features that provided MS/MS information (negative ion mode) and were consistently found in all CA extracts. (C) Correlation matrix between different *C. asiatica* accessions based on 5512 *m/z* features as the PCA. (D) Heatmap visualising area under the curve for the chromatographic peaks of the compounds presented in Figure 5. The area under the curve has been averaged across three replicates. The colours in the heatmap indicate the *z*‐score which was calculated by subtracting the mean of the peak areas for a metabolite across different samples and dividing it by the standard deviation of the metabolite across all the samples. The red colour indicates positive *z*‐score, the white colour indicates zero *z*‐score, whereas the blue colour indicates negative *z*‐score. Higher intensity of the colour in the scale indicates a higher magnitude of the *z*‐score. The dendrogram on the *x*‐axis indicates the degree of similarity between the metabolites, the closer the metabolites the higher the level of similarity in them and the metabolites have been clustered using hierarchical clustering. Similarly, the dendrogram on the *y*‐axis indicates the degree of similarity between the different samples (different CA accessions), the closer the samples the higher the level of similarity in them and they have been clustered using hierarchical clustering (Ward, Euclidean distance). PCA was performed using MetaboAnalyst V4.0. [Colour figure can be viewed at wileyonlinelibrary.com]

In order to capture the similarities and differences in metabolite composition observed for the eight CA accessions available to us, we used principal component analysis (PCA) on log‐transformed and Pareto‐scaled data using 5512 *m/z* features containing MS/MS spectral information in negative ion mode (Figure 2A). PCA revealed significant differences across the CA accessions. The PCA loading plots show the constituents with more variability among the *C. asiatica* accessions (Figure 2B). Di‐caffeoylquinic acids and triterpenes showed high variation between the different accessions. For additional contrasting of CA accessions, we also used as tools a correlation matrix and a heatmap based on area under the curve for extracted ion peaks. The correlation matrix aids in evaluating similarities and dissimilarities of extracts based on the correlation score (Figure 2C). Higher correlation scores (between 0.75 and 1) are indicated by red; scores between 0.74 and 0.51 are indicated by white; scores between 0.5 and 0.25 are represented by purple; and lower correlation scores (less than 0.25) are represented by blue. The Pearson correlation value calculated between samples CA6 and CA2 sample is 0.27, which indicates that there is a small linear relationship between CA2 and CA6. The Pearson correlation value calculated between CA2 and CA1 samples is 0.48, which also indicates a small linear relationship between CA2 and CA1 samples. The Pearson correlation between CA6 and CA4 samples is 0.87 which indicates that these two samples are linearly related, indicating similarities in metabolite contents for CA4 and CA6 extracts. The heatmap with hierarchical clustering (Figure 2D) visualises the precursor ion peak areas for 14 compounds evaluated in all CA extracts; these compounds were selected because they were present at relatively high concentration in all plant accessions and authentic standards were commercially available. Peak areas were averaged across three replicates. The dendrogram on the *y*‐axis indicates the degree of similarity or difference between the CA compound levels in the CA accessions, e.g. CA3 and CA8 are closer in the clustering tree indicating higher similarity of the compound levels in extracts CA3 and CA8, whereas extract CA6 is separated in the dendrogram from CA3 and CA8 indicting little similarity of the compound levels of the C6 extract with the levels found in the extracts of CA3 and CA8.

### 3.2 Structural similarity network

**FIGURE 3:**
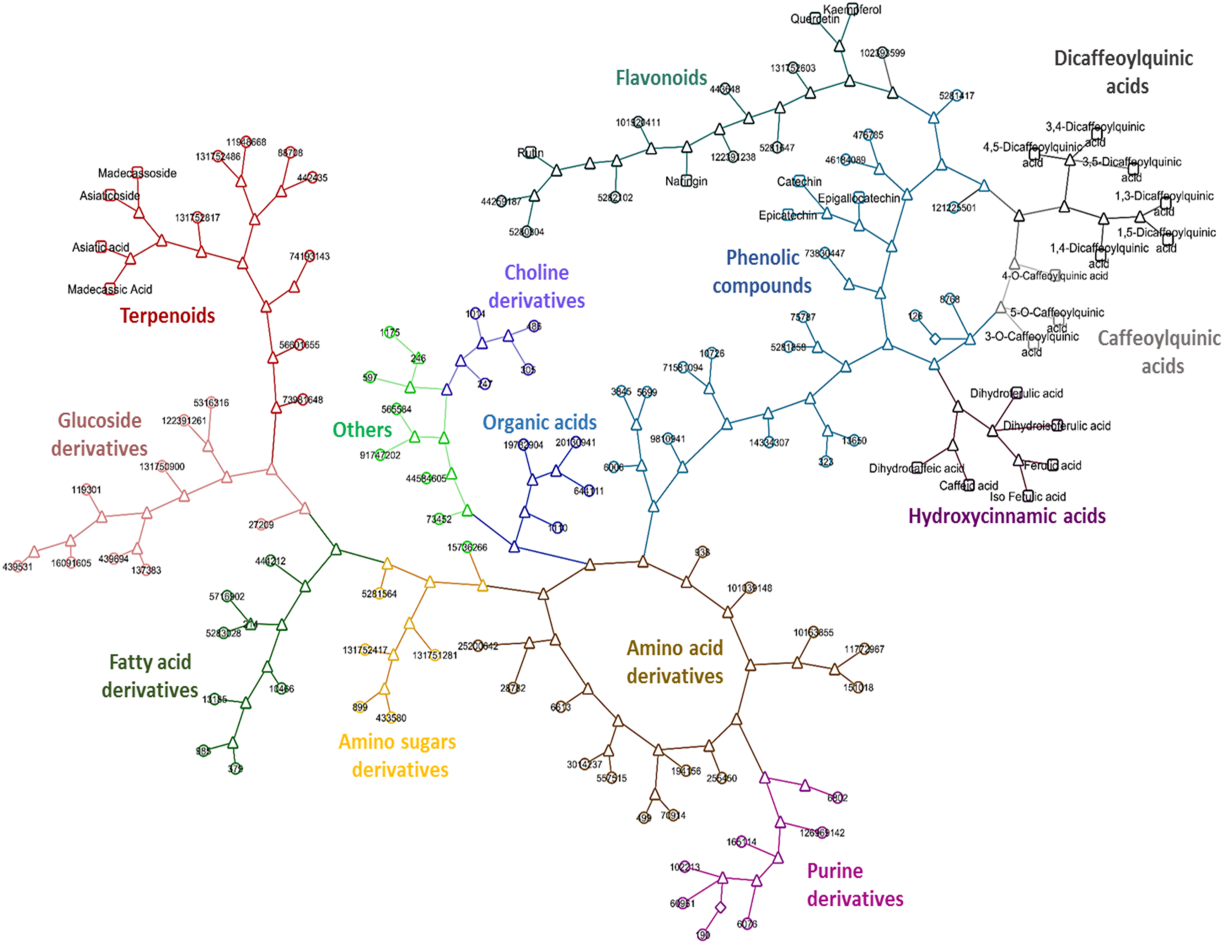
Cytoscape network for 117 assigned compounds in *Centella asiatica* described in Table 1. The clustering relationship is based on the molecular input line‐entry system (SMILES) as a fingerprint for the molecules being compared (Tanimoto coefficient). A Tanimoto coefficient greater than or equal to 0.68 indicates that the compounds being compared are structurally similar and statistically significant at the 95% confidence interval according to Kim *et al*..^51^ In this Cytoscape network, compounds are indicated by circular terminal nodes and labelled with their respective PubChem ID. Identified compounds, i.e. compounds for which authentic standards were available (Level 1 annotations), are indicated by a square node. Tanimoto scores greater than or equal to 0.68 are represented by triangular branch nodes, while scores less than 0.68 are depicted by diamond shaped branch nodes. [Colour figure can be viewed at wileyonlinelibrary.com]

Figure 3 shows a 2D structural similarity network of 117 assigned compounds (Table S1) found consistently in the aqueous extract of all eight CA accessions, which was created using the Tanimoto similarity score.^49^ Compounds are arranged in 14 interconnected clusters that are structurally similar at the boundary nodes at the 95% confidence level. We used the 2D structural similarity network to support our tentative annotations of metabolites and the associated categorisation into compound classes or clusters (Table 1). Compounds fall into the following clusters: 14 amino acid derivatives, five amino sugar derivatives, four choline derivatives, six di‐caffeoylquinic acids, seven fatty acid derivatives, 13 flavonoids, nine hexoside derivatives, five hydroxycinnamic acids, three mono‐caffeoylquinic acids, four organic acids, 21 phenolic compounds, seven purine derivatives, 12 terpenoids, and seven other compounds. Classification was established according to structural similarity (Tanimoto algorithm); consequently, some compounds may belong to more than one compound class. While *C. asiatica* is most known as a rich source of pentacyclic triterpenoids,^19^, ^24^ relatively high percentages of caffeoylquinic acids and flavonoids have also been identified.^19^, ^55^ These specialised metabolites, specifically phenylpropanoid derivatives, have been associated with *C. asiatica*'s anti‐inflammatory, antioxidant, or other biological activities.^60^, ^61^

Noteworthy, but beyond the current study, the enzymatic machinery required to produce specialised metabolites in most plants, including *C. asiatica*, is largely uncharacterised.^62^ The lack of intermediate specialised metabolites along the metabolic pathways makes this characterisation more complex. The detection of specialised metabolites that have not yet been reported in plant extracts, in conjunction with a Cytoscape network clustered according to the structural similarity (Tanimoto algorithm), can potentially help with characterising new metabolic pathways by searching for potential enzymes responsible for the interconversion of metabolites clustering together.

### 3.3 Accurate quantification of phytochemicals in extracts using precursor ion (MS1) quantification

In this work, we demonstrate the suitability of using a LC–MS/MS DDA method for the screening of compounds and molecular ion (MS1) extraction for quantification of selected compounds in the same chromatographic run in conjunction with external calibrations for the selected marker compounds. From over 20000 recorded *m/z* features, the 5512 and 6906 most prominent *m/z* features acquired in negative (Figure 1) and positive ion mode, respectively, were fragmented in the DDA experiment. This untargeted approach provides a spectral library for thousands of potential compounds that can be mined in future applications. From the 117 tentatively assigned compounds (Table 1), 24 compounds were selected as a proof of concept for the MS1‐based quantification, including three mono‐caffeoylquinic acids, five di‐caffeoylquinic acids, seven flavonoids, five hydroxycinnamic acid derivatives and four triterpenes (structures shown in Figure S4).

### 3.4 Method validation for selected compounds

For accuracy, precision, repeatability, linearity, LOD, LOQ and range, the proposed method followed the typical validation procedure in accordance with the ICH Harmonised Tripartite Guideline.^63^

Figure S5 shows LC–MS XICs obtained from authentic standards of the 24 compounds that were selected as phytochemical marker compounds. Analytical parameters, namely [M‐H]^−^ *m/z*, RT, accuracy for three different concentration levels, LOD, LOQ and inter‐day coefficient of variation [relative standard deviation (RSD)] were established for the 24 phytochemical markers using precursor ion (MS1) extraction (Table 2). The analytical accuracy for three known concentration samples at the low (0.05 ppm), medium (0.50 ppm) and high (5.0 ppm) calibration curve intervals ranged from 87 to 125% (Table 2). The RSD was measured for a solution of 1 mg/L and ranged from 6.8 to 24% for nine repetitions measured in a span of 6 months (Table 2).

**TABLE 2:** Analytical parameters for authentic standards. Exact *m/z* used for extracted ion chromatogram (XIC), retention times (RTs), limit of detection (LOD) and limit of quantification (LOQ), percentage of accuracy for three concentrations, and percentage of relative standard deviation (%RSD) are given for 24 selected compounds. Compounds are sorted by RT

Compound	[M‐H]^‐ a^	RT^b^ (min)	LOD^c^ (μg/L)	LOQ^d^ (μg/L)	Low QC^e^ 0.05 mg/L	Medium QC^e^ 0.5 mg/L	High QC^e^ 5 mg/L	%RSD^f^
5‐*O*‐Caffeoylquinic acid	353.0867	11.85	0.078	0.260	109	108	93	13.11
Epigallocatechin	305.0656	13.48	0.146	0.485	101	104	99	21.70
Catechin	289.0707	14.48	0.018	0.060	123	106	98	11.54
Dihydrocaffeic acid	181.0495	14.85	0.005	0.015	109	117	93	11.18
4‐*O*‐Caffeoylquinic acid	353.0867	15.08	0.040	0.134	125	116	93	12.13
3‐*O*‐Caffeoylquinic acid	353.0867	15.30	0.040	0.134	122	116	97	17.33
Caffeic acid	179.0339	15.83	0.319	1.064	111	110	99	11.71
Epicatechin	289.0707	16.80	0.013	0.045	107	100	98	12.52
1,5‐Di‐caffeoylquinic acid	515.1184	17.49	0.061	0.202	110	104	97	15.32
1,3‐Di‐caffeoylquinic acid	515.1184	17.49	0.061	0.202	110	104	97	15.32
Rutin	609.145	18.96	0.016	0.052	107	102	100	11.65
Dihydroferulic acid	195.0652	19.02	0.027	0.089	123	104	96	8.98
3,4‐Di‐caffeoylquinic acid	515.1184	19.11	0.169	0.562	105	99	100	15.30
3,5‐Di‐caffeoylquinic acid	515.1184	19.45	0.166	0.552	106	100	102	15.25
Ferulic acid	193.0495	19.55	0.001	0.004	111	105	93	6.79
4,5‐Di‐caffeoylquinic acid	515.1184	19.92	0.108	0.358	101	99	102	12.09
Naringin	579.1708	20.07	0.025	0.083	97	100	88	10.66
Isoferulic acid	193.0495	20.42	0.001	0.003	94	102	103	16.39
Quercetin	301.0342	21.10	0.068	0.227	111	100	97	12.15
Madecassoside	973.5003	21.47	0.008	0.025	110	97	87	16.94
Asiaticoside	957.5054	21.97	0.001	0.004	102	99	98	18.87
Kaempferol	285.0390	22.01	0.028	0.092	112	103	97	10.46
Madecassic acid	503.3367	23.74	0.009	0.031	97	100	97	11.41
Asiatic acid	487.3418	24.41	0.005	0.015	96	95	92	12.61

a Exact mass; negative ionisation; mass error < 5 ppm.

b Retention time.

c Calibration detection limit evaluated as *S/N* ratio 3:1.

d Calibration quantification limit evaluated as *S/N* ratio 10:1.

e % of accuracy = (measured concentration/true concentration) × 100 in quality control sample.

f %RSD measured for 1 mg/L based on nine measurements over a span of 6 months. Values were calculated using the following equation %RSD = (standard deviation/average) × 100 in Microsoft Excel 2016.

The availability of high‐resolution accurate mass data allowed us to obtain comprehensive fingerprints for botanical extracts that can be further interrogated post acquisition to obtain accurate quantification of phytochemicals by extracting the precursor ions and using the area under the peak for quantification (MS1 quantification) in the same analytical run. Quantified compounds showed good linearity over three orders of magnitude (0.005–5.0 mg/L, *r* > 0.990, Table S2).

Matrix effects are frequently observed when analysing complex samples. In order to evaluate matrix effects in the CA extracts, pooled CA extract samples were spiked with the 24 available standards. TICs obtained for a CA extract and the same sample after standard addition is shown in Figure S6. For plant extracts, recoveries of individual compounds ranged from 71 to 144% and 91 to 132% for 0.25 and 5.0 ng on‐column, respectively (Table 3), confirming the feasibility of the proposed procedure for quantitative analysis of marker compounds in CA extracts.

**TABLE 3:** Recovery experiment. Recovery percentage and mean concentration of individual quantified compounds were measured in a pooled CA sample (100 mg/L) using precursor ions with respective standard deviations obtained without standard addition and after addition of a mixture of 24 standards in two different concentration levels (0.25 and 5 ng of each standard on‐column). All measurements are given in nanograms

Compound	QC (ng on column)	QC + 0.25 ng standards	% Recovery^a^	QC +5 ng^b^ standards	% Recovery^a^
5‐*O*‐Caffeoylquinic acid	1.02 ± 0.01	1.28 ± 0.01	102	6.53 ± .46	110
Epigallocatechin	<LOQ^a^	0.18 ± 0.02	71	4.55 ± 0.20	91
Catechin	<LOQ	0.33 ± 0.09	134	5.88 ± 0.32	117
Dihydrocaffeic acid	<LOQ	0.28 ± 0.01	113	5.34 ± 0.27	107
4‐*O*‐Caffeoylquinic acid	0.88 ± 0.02	1.17 ± 0.03	115	6.31 ± 0.09	109
3‐*O*‐Caffeoylquinic acid	2.45 ± 0.03	2.79 ± 0.09	133	7.68 ± 0.10	105
Caffeic acid	0.67 ± 0.07	0.97 ± 0.03	123	6.06 ± 0.43	108
Epicatechin	<LOQ	0.30 ± 0.06	119	5.57 ± 0.14	111
1,5‐Di‐caffeoylquinic acid	0.38 ± 0.01	0.68 ± 0.06	121	5.91 ± 0.28	110
1,3‐Di‐caffeoylquinic acid	0.38 ± 0.01	0.68 ± 0.06	121	5.91 ± 0.28	110
Rutin	0.04 ± 0.01	0.31 ± 0.02	106	5.44 ± 0.35	108
Dihydroferulic acid	<LOQ	0.25 ± 0.01	101	5.71 ± 0.17	114
3,4‐Di‐caffeoylquinic acid	3.23 ± 0.02	3.57 ± 0.02	136	8.86 ± 0.17	113
3,5‐Di‐caffeoylquinic acid	3.78 ± 0.02	4.14 ± 0.03	144	8.94 ± 0.50	103
Ferulic acid	0.11 ± 0.01	0.40 ± 0.04	118	5.59 ± 0.11	109
4,5‐Di‐caffeoylquinic acid	3.93 ± 0.01	4.16 ± 0.05	92	9.16 ± 0.10	105
Naringin	<LOQ	0.29 ± 0.03	117	5.86 ± 0.84	117
Isoferulic acid	0.18 ± 0.01	0.52 ± 0.04	135	6.35 ± 0.21	123
Quercetin	0.29 ± 0.22	0.56 ± 0.17	109	5.94 ± 0.38	113
Madecassoside	25.5 ± 0.95	25.77 ± 0.07	108	31.6 ± 2.20	110
Asiaticoside	10.74 ± 0.12	11.0 ± 0.16	114	16.1 ± 0.55	108
Kaempferol	0.31 ± 0.02	0.62 ± 0.01	121	5.79 ± 0.49	109
Madecassic acid	1.35 ± 0.01	1.65 ± 0.03	120	7.58 ± 0.24	124
Asiatic acid	0.62 ± 0.02	0.97 ± 0.28	138	7.25 ± 0.53	132

a % Recovery = (Cf − Ci)/Cspiked × 100. Ci, nanograms measured (on‐column) before standard addition; Cf, nanograms measured after standard addition; Cspiked, nanograms of spiked standard.

b Calibration quantification limit evaluated as S/N ratio 10:1.

A range of three orders of magnitude is typical for time‐of‐flight (TOF) analysers, which is a disadvantage when compared with the dynamic range of triple quadrupole analysers, which usually feature a linear dynamic range that extends over six orders of magnitude. Nevertheless, the high resolution allows us to obtain chemical fingerprint and quantification of marker compounds in the same analytical run,^64^ saving instrument time and solvents, and avoiding sample degradation due to storage.

For the developed quantification method, the combination of an optimised separation method with a high resolution quadrupole time‐of‐flight (q‐TOF) mass spectrometer allowed the detection and quantification of phytochemicals in plant extracts at sub‐parts per billion levels (except caffeic acid; LOQ 1.06 μg/L) with minimum sample processing. Modern q‐TOF mass spectrometers possess sensitivities typically associated with MS/MS‐based selected reaction monitoring (SRM) methods. Reported LOD values for 15 phenolic acids and 17 flavonoids acquired using SRM in a triple quadrupole mass spectrometer range from 3.4 to 228 μg/L^65^ and are comparable with our LODs. Contemporary q‐TOF instruments typically offer mass resolving power of ≥ 25000 [full width at half maximum (FWHM)] at *m/z* 195. These q‐TOF platforms obtain accurate mass measurements with high resolution for precursor ions and fragment ions thus allowing structural characterisation and quantification of phytochemicals in complex mixtures with high confidence.

### 3.5 Quantification of phytochemical marker compounds in CA extracts from different sources

In previous studies, comparisons of specialised metabolite production of *C. asiatica* were limited to four triterpenoids (asiatic acid, madecassic acid and their glycosides asiaticoside and madecassoside).^55^, ^66^, ^67^, ^68^, ^69^, ^70^, ^71^ Some other compounds (flavonoids and caffeoyl esters) were analysed by LC–MS^31^ and HPLC‐DAD (diode array detector).^12^, ^55^, ^72^, ^73^ A comparative study of nutrient content and yield performance of *C. asiatica* at different harvesting periods was reported. The study focused on yield measured by dry weight of leaves and on nutrient comparisons [phosphorus (P), potassium (K), sulphur (S), calcium (Ca), magnesium (Mg), zinc (Zn), copper (Cu), iron (Fe), manganese (Mn) and nitrogen (N)].^74^

**FIGURE 4:**
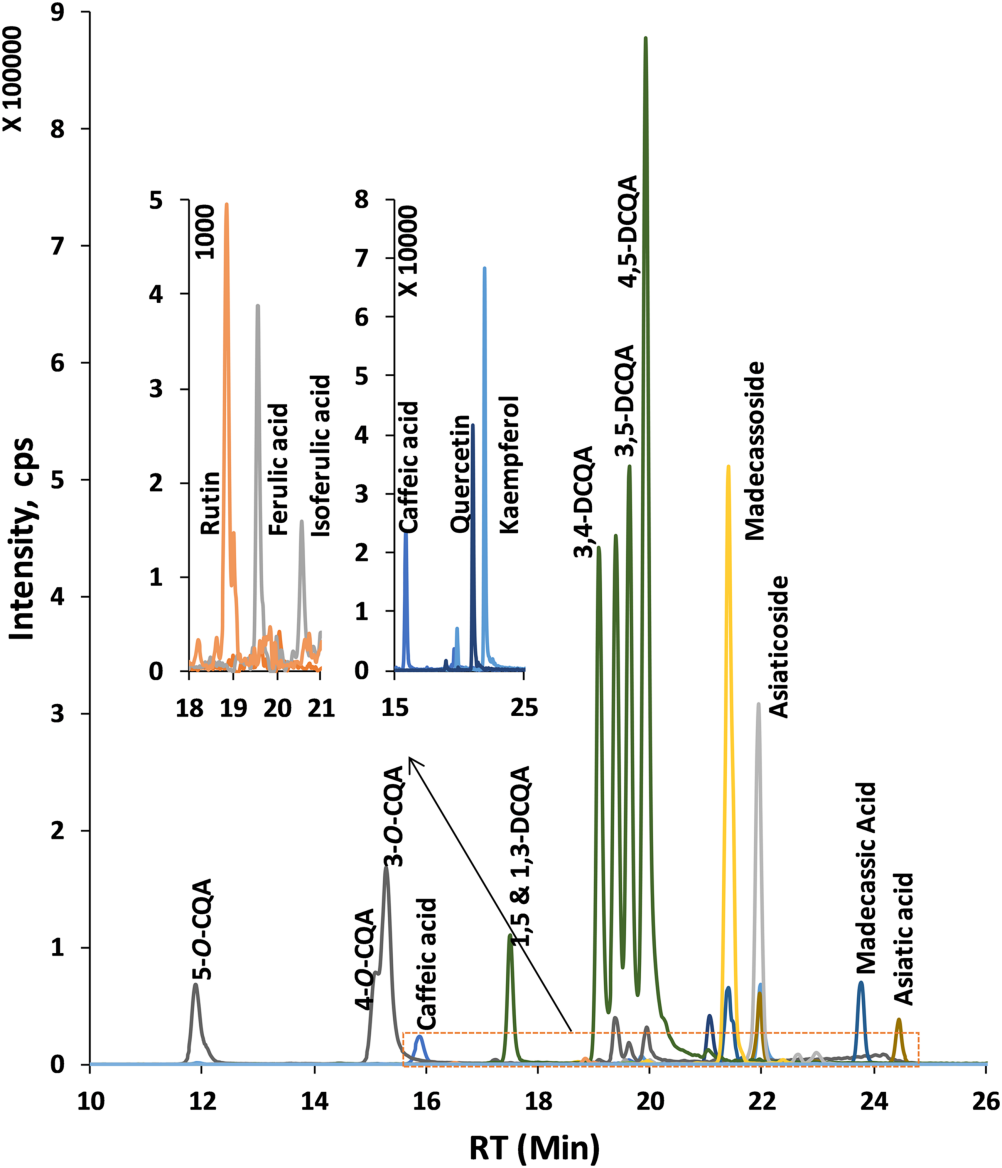
Extracted ion chromatograms (XICs) of 18 compounds that were used for precursor ion (MS1) quantification. Individual analytical parameters are shown in Table 2. XICs were obtained using the data‐dependent acquisition (DDA) (ESI−) mode obtained for a pooled *Centella asiatica* (CA) sample. [Colour figure can be viewed at wileyonlinelibrary.com]

**FIGURE 5:**
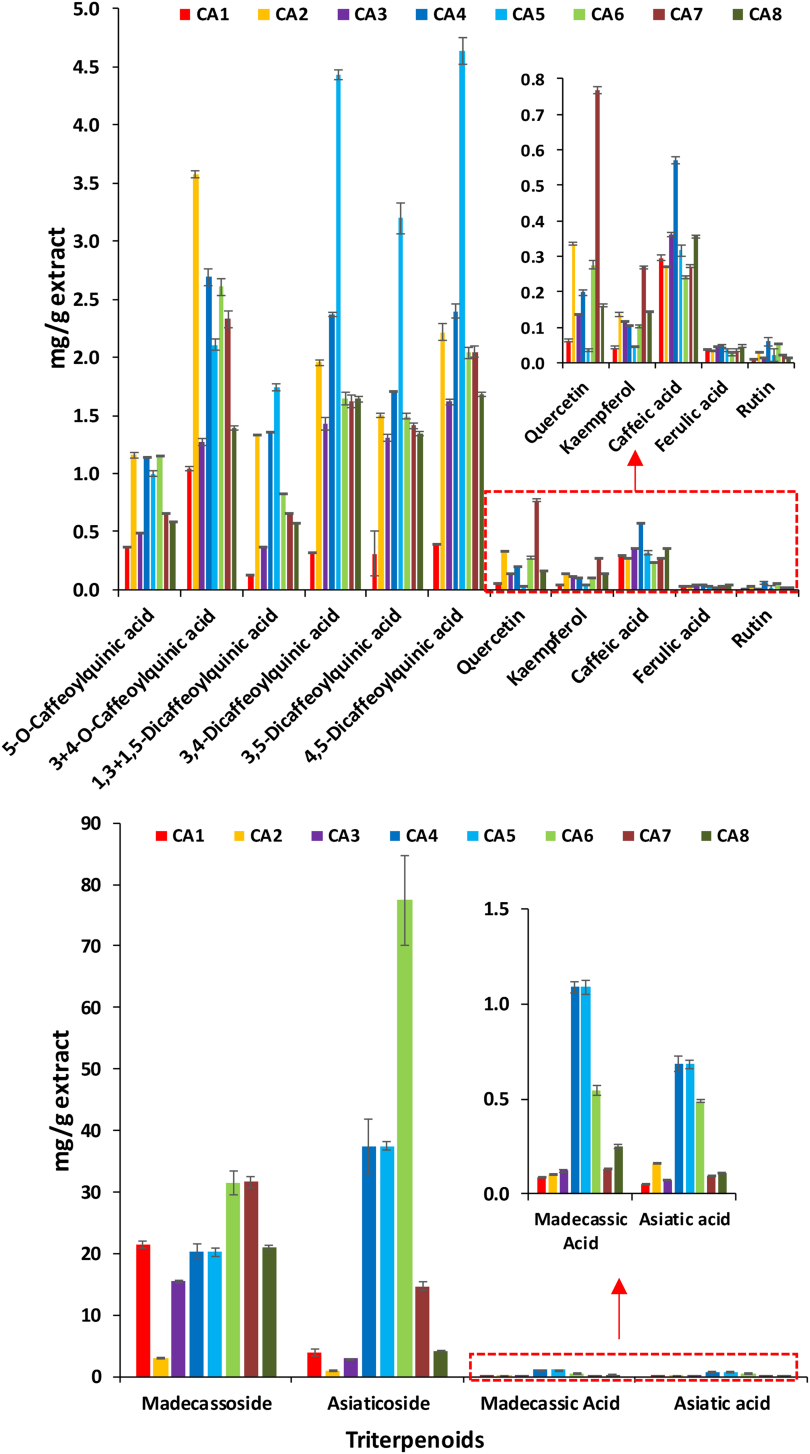
Precursor ion (MS1) quantification of 15 phytochemicals in the eight different *Centella asiatica* accessions (water extracts). Results are presented as milligrams per gram of dry extract; standard error derived from triplicates analysis. [Colour figure can be viewed at wileyonlinelibrary.com]

Our study shows that CA extracts were particularly rich in mono‐caffeoylquinic acids, such as 3‐caffeoylquinic acid, 4‐caffeoylquinic acid and 5‐caffeoylquinic acid, and di‐caffeoylquinic acids, such as 1,3‐di‐caffeoylquinic acid, 1,5‐di‐caffeoylquinic acid, 3,4‐di‐caffeoylquinic acid, 3,5‐di‐caffeoylquinic acid and 4,5‐di‐caffeoylquinic acid, as well as some triterpenoids such asiaticoside, madecassoside and their aglycones (Table 3, Figures 4 and 5). CA extracts also contained several flavonoids and hydroxycinnamic acid derivatives. Figure S8 compiles positive matches with certified standard compounds. Figure 4 shows XICs for 18 selected compounds quantified in CA water extracts using area under the curve of the precursor ion acquired in DDA mode.

Under the HPLC conditions used, 1,3‐ and 1,5‐di‐caffeoylquinic acid co‐elute since they are stereoisomers (structures shown in Figure S4). For all other compounds quantified in this study, the combination of suitable separation conditions with extraction of molecular ion (MS1) chromatograms at accurate *m/z* values (Figure S5, Table 2) enabled detection limits in the low nanomolar to picomolar range for 24 phytochemicals (Tables 2 and S2). The optimised analytical procedure minimised interferences by improving the chromatographic separation of isomers with similar fragmentation patterns and thereby also optimised detection limits.

In this study, eight accessions of *C. asiatica* were quantified using precursor ion (MS1) quantification (Figure 5). Comparing samples CA6 and CA1, the concentrations of asiaticoside varied by 19.9‐fold, asiatic acid by 9.1, and madecassoside by 1.5. In the case of di‐caffeoylquinic acids, comparing CA5 and CA1, 4,5‐dicaffeoylquinic acid varied 11‐fold. Mono‐caffeoylquinic acids presented less variation across the accessions (Figure 5). This emphasises the importance of establishing rigorous analytical procedures for botanical extracts and supplements to ensure product integrity and batch‐to‐batch reproducibility.

To conclude, we developed a method for untargeted and targeted characterisation of CA extracts using the same chromatographic run. The combination of suitable separation conditions with mass spectral data acquired with high resolving power using DDA enables the extraction of high resolution accurate mass precursor ions with exact *m/z* values that allows accurate quantification of phytochemicals with LOQs at 1.06 μg/L or lower. The described method was validated for the quantification of (a) seven flavonoids, (b) three structural isomers of caffeoylquinic acids, (c) five di‐caffeoylquinic acids, (d) five caffeic acids derivatives and (e) four terpenoids. The concentration of targeted metabolites across different CA accessions was substantial, with the largest difference (20‐fold) observed for asiaticoside between CA1 versus CA6, demonstrating that standardisation and detailed characterisation of plant extracts are prerequisites for reliable and reproducible studies aiming to determine the biological activity of CA and botanical extracts in general.

Overall, the current study underscores the need for methods to efficiently analyse highly complex plant extracts to support the standardisation of botanicals destined for preclinical studies, clinical trials and commercial products.

### 3.6 Mass spectrometry data deposition

MS/MS data have been deposited to the GNPS repository (http://gnps.ucsd.edu) with the dataset identifiers MSV000084588 (ESI+) and MSV000084621 (ESI−).

## Supporting information


**Table S1.** Additional parameters for 117 identified or tentatively identified compounds detected in *Centella asiatica* aqueous extracts using positive and negative ion mode. Compounds confirmed using authentic standards are shown in bold. For tentatively assigned compounds (L2 annotations) the MS/MS spectral matches that supported the annotation are compiled in Figure S7.
**Table S2.** Linear regression functions calculated for 24 precursor ions. Calibration curves were stablished in negative ion mode with correlation factor *r* > 0.990. Calibration curves were prepared from mixed standard solutions containing analytical blanks, 0.005, 0.01, 0.05, 0.10, 0.50, 1.00, 5.0 and 10.0 mg/L. Compounds are sorted by retention time.
**Figure S1.** Untargeted workflow approach. This workflow yielded 24 identified (L1 annotation) and 93 tentatively assigned (L2) compounds (117 in total). Compound description is detailed in Table S1.
**Figure S2.** Examples of extracted ion chromatograms of compounds detected in *Centella asiatica* water extract by data‐dependent LC–MS/MS analysis in the positive ionisation mode (ESI+). Table S1 compiles retention time and mass spectral data for identified (L1 annotation) and tentatively annotated (L2) compounds including retention time, *m/z*, molecular formula and detected adducts.
**Figure S3.** Examples of extracted ion chromatograms of compounds detected in *Centella asiatica* water extract by data‐dependent LC–MS/MS analysis in the negative ionisation mode (ESI‐). Table S1 compiles retention time and mass spectral data for identified (L1 annotation) and tentatively annotated (L2) compounds including retention time, *m/z*, molecular formula and detected adducts.
**Figure S4.** Chemical structures of compounds for which authentic standards were available and which were selected as marker compounds for quantification. Three mono‐caffeoylquinic acids (green), five di‐caffeoylquinic acids (magenta), seven flavonoids (purple), five hydroxycinnamic acid derivatives (black) and four triterpenes (blue).
**Figure S5.** Extracted ion chromatograms obtained for the calibration solution containing the 24 selected phytochemical marker compounds (1 mg/L each). Negative ion mode, extracted *m/z* values are indicated in the figure. Analytical parameters are shown in Table 2.
**Figure S6.** Standard addition experiment. Total ion chromatogram (TIC) obtained for *Centella asiatica* (CA) water extract (solid line, 10 μL injection) and same sample after standard addition (dotted line). For the standard addition experiment, 1.0 mL of standard mix containing 1.0 mg/L of each compound was added to 1.0 mL of the pooled CA sample (200 mg/L).
**Figure S7.** MS/MS spectra of compounds in *Centella asiatica* extracts (pooled CA sample) that were assigned tentatively (L2 annotations) by extensive querying and comparison with spectral libraries [including METLIN, our in‐house library, ChEBI, and the Human Metabolite Database (HMDB)] using Progenesis QI™ and applying the workflow shown in Figure S1. Red lines were matches against the databases. Eighty‐seven compounds that were detected in *C. asiatica* aqueous extracts and tentatively assigned but have not been reported for *C. asiatica* as of to date are denoted with an asterisk ‘*’ in Table 1. The number shown in the spectra matches the entry number # in Table S1. MS/MS scores are indicated in square brackets ‘[]’ and were obtained using Progenesis QI. GNPS identifiers are provided in curly brackets ‘{}’.
**Figure S8.** MS/MS spectra for compounds present in *Centella asiatica* (CA) water extracts that were identified using authentic standards (L1 annotations). MS/MS score is indicated in square brackets ‘[]’.
**Figure S9.** Recovery of marker compounds using different sonication times. Injection of 10 μL of *Centella asiatica* water extract (0.1 mg/mL). Sonication was for 15 min (pink line) and 30 min (blue line). The 30 min increases the recovery of less hydrophilic compounds such as di‐caffeoylquinic acids with no degradation of mono‐caffeoylquinic acids.
