# Supplementary material for: Integration of mass spectral fingerprinting analysis with precursor ion (MS1) quantification for the characterisation of botanical extracts: application to extracts of Centella asiatica (L.) Urban
Source: Phytochem Anal. 2020 Apr 12;31(6):722–38. doi: 10.1002/pca.2936 (PMC7587007; doi:10.1002/pca.2936)
Supplement: Supplementary file 1 — Table S1. Additional parameters for 117 identified or tentatively identified compounds detected in Centella asiatica aqueous extracts using positive and negative ion mode. Compounds confirmed using authentic standards are shown in bold. For tentatively assigned compounds (L2 annotations) the MS/MS spectral matches that supported the annotation are compiled in Figure S7. Table S2. Linear regression functions calculated for 24 precursor ions. Calibration curves were stablished in negative ion mode with correlation factor r > 0.990. Calibration curves were prepared from mixed standard solutions containing analytical blanks, 0.005, 0.01, 0.05, 0.10, 0.50, 1.00, 5.0 and 10.0 mg/L. Compounds are sorted by retention time. Figure S1. Untargeted workflow approach. This workflow yielded 24 identified (L1 annotation) and 93 tentatively assigned (L2) compounds (117 in total). Compound description is detailed in Table S1. Figure S2. Examples of extracted ion chromatograms of compounds detected in Centella asiatica water extract by data‐dependent LC–MS/MS analysis in the positive ionisation mode (ESI+). Table S1 compiles retention time and mass spectral data for identified (L1 annotation) and tentatively annotated (L2) compounds including retention time, m/z, molecular formula and detected adducts. Figure S3. Examples of extracted ion chromatograms of compounds detected in Centella asiatica water extract by data‐dependent LC–MS/MS analysis in the negative ionisation mode (ESI‐). Table S1 compiles retention time and mass spectral data for identified (L1 annotation) and tentatively annotated (L2) compounds including retention time, m/z, molecular formula and detected adducts. Figure S4. Chemical structures of compounds for which authentic standards were available and which were selected as marker compounds for quantification. Three mono‐caffeoylquinic acids (green), five di‐caffeoylquinic acids (magenta), seven flavonoids (purple), five hydroxycinnamic acid derivatives (black) and [file PCA-31-722-s001.docx]

**Supporting Information**

**Integration of mass spectral fingerprinting analysis with precursor ion [MS1] quantification for the characterization of botanical extracts: Application to extracts** **of** ***Centella asiatica***

**Armando Alcazar Magana^1,5^, Kirsten Wright^2^, Ashish Vaswani^1^, Maya Caruso^2^, Ralph L. Reed^4,5^, Conner F. Bailey^1^, Thuan Nguyen^6^, Nora E. Gray^2^, Amala Soumyanath^2^, Joseph Quinn^2,3^, Jan F. Stevens^4,5^, and Claudia S. Maier^1,5*^**

^1^Department of Chemistry, Oregon State University, 153 Gilbert Hall, Corvallis, Oregon 97331

^2^Department of Neurology, Oregon Health and Science University, Portland, Oregon

^3^Department of Neurology and Parkinson’s Disease Research Education and Clinical Care Center (PADRECC), VA Portland Healthcare System, Portland, Oregon

^4^Department of Pharmaceutical Sciences, Oregon State University, 1601 SW Jefferson way, Corvallis, Oregon 97331

^5^Linus Pauling Institute, Oregon State University, 2900 SW Campus way, Corvallis, Oregon 97331

^6^OHSU-PSU School of Public Health, Oregon Health & Science University, Portland, Oregon

***** [claudia.maier@oregonstate.edu](mailto:claudia.maier@oregonstate.edu)

**Table of Contents**

| Additional parameters for 117 identified or tentatively identified compounds detected in Centella asiatica aqueous extracts. | ………….….Table S-1 |
| --- | --- |
| Linear regression functions calculated for 24 precursor ions | ………….….Table S-2 |
| Untargeted workflow approach | …………....Figure S-1 |
| Examples of extracted ion chromatograms (positive ion mode). | ……….…...Figure S-2 |
| Examples of extracted ion chromatograms (negative ion mode). | …………....Figure S-3 |
| Chemical structures of authentic standards | …………....Figure S-4 |
| Extracted ion chromatograms obtained for the calibration solution | …………....Figure S-5 |
| Standard addition experiment | …………....Figure S-6 |
| MS/MS spectra of tentatively identified compounds (L2 annotations) | ………....Figure S-7a |
| MS/MS spectra for compounds present at relatively high concentration matched with authentic standards | ……….. Figure S-7b |
| Recovery of marker compounds using different sonication time | …………..Appendix 1 |
|  |  |

**SUPPLEMENTAL TABLES**

**Table S1.** Additional parameters for 117 identified or tentatively identified compounds detected in *Centella asiatica* aqueous extracts using positive and negative ion mode. Compounds confirmed using authentic standards are shown in bold. For tentatively assigned compounds (L2 annotations) the MS/MS spectral matches that supported the annotation are compiled in Figure S7.

| **#^a^** | **Accepted Description** | **m/z** | **RT (min)** | **Detected Adducts** | **Δ**  **ppm** | **Neutral Formula^b^** | **PubChemCID** |
| --- | --- | --- | --- | --- | --- | --- | --- |
| 1 | **1,3-Dicaffeoylquinic acid^c^** | 515.1184 | 17.49 | [M-H]^-^ | 2.3 | C_25_H_24_O_12_ | 6474640 |
| 2 | 1,4-Dicaffeoylquinic acid | 515.1184 | 19.71 | [M-H]^-^ | 2.3 | C_25_H_24_O_12_ | 12358846 |
| 3 | **1,5-Dicaffeoylquinic acid** | 515.1184 | 17.49 | [M-H]^-^ | 2.3 | C_25_H_24_O_12_ | 5281769 |
| 4 | 12-Oxodihydrophytodienoic acid | 317.2090 | 25.11 | [M+H-H_2_O]^+^, [M+Na]^+^ | 1.6 | C_18_H_30_O_3_ | 5716902 |
| 5 | 16-hydroxypalmitic acid | 271.2271 | 24.81 | [M-H]^-^ | 3.1 | C_16_H_32_O_3_ | 10466 |
| 6 | 1-beta-D-Glucopyranosyl-L-tryptophan | 367.1501 | 12.29 | [M+H]^+^, [M+Na]^+^ | 1.1 | C_17_H_22_N_2_O_7_ | 11772967 |
| 7 | 1-Caffeoyl-5-feruloylquinic acid | 553.1296 | 19.16 | [M+Na]^+^ | 3.3 | C_26_H_26_O_12_ | 121225501 |
| 8 | 2,6-Piperidinedicarboxylic acid | 174.0754 | 5.94 | [M+H]^+^ | 2.3 | C_7_H_11_NO_4_ | 557515 |
| 9 | 26-(2-Glucosyl-6-acetylglucosyl]-1,3,11,22-tetrahydroxyergosta-5,24-dien-26-oate | 863.4065 | 23.57 | [M+Na-2H]^-^ | 1.8 | C_42_H_66_O_17_ | 131752817 |
| 10 | 2'-O-Methyladenosine | 282.1195 | 13.45 | [M+H]^+^, [M+Na]^+^ | 0.4 | C_11_H_15_N_5_O_4_ | 102213 |
| 11 | 2-Pyrrolidone-5-carboxylic acid | 130.0491 | 7.54 | [M+H]^+^ | 3.8 | C_5_H_7_NO_3_ | 499 |
| 12 | 3 Hydroxycoumarin | 163.0380 | 15.27 | [M+H]^+^ | 4.2 | C_9_H_6_O_3_ | 13650 |
| 13 | **3,4-Dicaffeoylquinic acid** | 515.1189 | 19.11 | [M-H]^-^,  [M+Na-2H]^-^ | 1.3 | C_25_H_24_O_12_ | 5281780 |
| 14 | 3,4-Dihydroxybenzaldehyde | 139.0382 | 14.29 | [M+H]^+^ | 3.5 | C_7_H_6_O_3_ | 8768 |
| 15 | **3,5-Dicaffeoylquinic acid** | 515.1189 | 19.45 | [M-H]^-^,  [M+Na-2H]^-^ | 1.3 | C_25_H_24_O_12_ | 6474310 |
| 16 | 3,5-Dihydroxy-2-methylphenyl beta-D-glucopyranoside | 325.0896 | 9.73 | [M+Na]^+^ | 1.3 | C_13_H_18_O_8_ | 46184089 |
| 17 | 3,5-Dihydroxyphenyl 1-O-(6-O-galloyl-beta-D-glucopyranoside) | 463.0845 | 13.58 | [M+H-H_2_O]^+^, [M+Na]^+^ | 0.1 | C_19_H_20_O_12_ | 131752603 |
| 18 | 3-Hydroxy-2-oxo-3-phenylpropanoic acid | 163.0375 | 19.95 | [M+H-H_2_O]^+^ | 4.6 | C_9_H_8_O_4_ | 71581094 |
| 19 | **3-O-Caffeoylquinic acid** | 353.0872 | 15.28 | [M-H]^-^ | 1.9 | C_16_H_18_O_9_ | 1794427 |
| 20 | **4,5-Dicaffeoylquinic acid** | 515.1184 | 19.91 | [M-H]^-^ | 2.3 | C_25_H_24_O_12_ | 6474309 |
| 21 | 4-Guanidinobutanoic acid | 146.0915 | 5.45 | [M+H]^+^ | 4.3 | C_5_H_11_N_3_O_2_ | 25200642 |
| 22 | 4-Hydroxybenzaldehyde | 121.0297 | 17.04 | [M-H]^-^ | 1.0 | C_7_H_6_O_2_ | 126 |
| 23 | **4-O-Caffeoylquinic acid** | 353.0874 | 11.86 | [M-H]^-^, [M+Na-2H]^-^ | 1.4 | C_16_H_18_O_10_ | 9798666 |
| 24 | 5'-Deoxy-5'-(methylsulfinyl)adenosine | 314.0917 | 11.71 | [M+H]^+^, [M+Na]^+^ | 0.7 | C_11_H_15_N_5_O_4_S | 165114 |
| 25 | 5-Methoxy-L-tryptophan | 217.0970 | 14.58 | [M+H-H_2_O]^+^ | 2.6 | C_12_H_14_N_2_O_3_ | 151018 |
| 26 | 5-Methoxysalicylic acid | 167.0351 | 17.38 | [M-H]^-^ | 0.3 | C_8_H_8_O_4_ | 75787 |
| 27 | **5-O-Caffeoylquinic acid** | 353.0874 | 11.86 | [M-H]^-^,  [M+Na-2H]^-^ | 1.4 | C_16_H_18_O_9_ | 5280633 |
| 28 | 6-Amino-9H-purine-9-propanoic acid | 208.0837 | 9.09 | [M+H]^+^ | 5.2 | C_8_H_9_N_5_O_2_ | 255450 |
| 29 | 6-C-(alpha-L-arabinosyl)-8-C-(beta-L-arabinosyl)apigenin | 557.1259 | 17.40 | [M+Na]^+^ | 0.8 | C_25_H_26_O_13_ | 122391238 |
| 30 | 6-Docosenamide | 360.3234 | 26.15 | [M+H]^+^, [M+Na]^+^ | 0.1 | C_22_H_43_NO | 44584605 |
| 31 | 6-Oxo-2-piperidinecarboxylic acid | 126.0541 | 12.80 | [M+H-H_2_O]^+^ | 1.1 | C_6_H_9_NO_3_ | 3014237 |
| 32 | 8-Acetoxy-4'-methoxypinoresinol 4-glucoside | 593.2188 | 19.59 | [M+H]^+^ | 6.4 | C_29_H_36_O_13_ | 73830447 |
| 33 | Adenine | 136.0612 | 7.80 | [M+H]^+^ | 2.2 | C_5_H_5_N_5_ | 190 |
| 34 | Adenosine | 312.0945 | 9.09 | [M-H]^-^,  [M+FA-H]^-^ | 0.8 | C_10_H_13_N_5_O_4_ | 60961 |
| 35 | Aesculin | 363.0688 | 14.37 | [M+H]^+^, [M+Na]^+^ | 1.0 | C_15_H_16_O_9_ | 5281417 |
| 36 | Apimaysin | 541.1355 | 4.67 | [M-H_2_O-H]^-^ | 1.0 | C_27_H_28_O_13_ | 101920411 |
| 37 | **Asiatic acid** | 487.3421 | 21.91 | [M-H]^-^ | 1.8 | C_30_H_48_O_5_ | 119034 |
| 38 | **Asiaticoside** | 957.5101 | 20.50 | [M-H]^-^ | 3.8 | C_48_H_78_O_19_ | 24721205 |
| 39 | Astragalin | 447.0916 | 20.07 | [M-H]^-^ | 3.9 | C_21_H_20_O_11_ | 5282102 |
| 40 | b-Chlorogenin 3-[4'-(2'-glucosyl-3'-xylosylglucosyl)galactoside] | 1049.5217 | 21.01 | [M-H]^-^ | 4.0 | C_50_H_82_O_23_ | 74193143 |
| 41 | Betaine | 118.0853 | 4.00 | [M+H]^+^ | 5.7 | C_5_H_11_NO_2_ | 247 |
| 42 | **Caffeic acid** | 179.0350 | 15.86 | [M-H]^-^ | 0.3 | C_9_H_8_O_4_ | 689043 |
| 43 | cAMP | 328.0448 | 7.85 | [M-H]^-^ | 1.6 | C_10_H_12_N_5_O_6_P | 6076 |
| 44 | Caprylic acid | 125.0977 | 21.76 | [M-H_2_O-H]^-^ | 5.4 | C_8_H_16_O_2_ | 379 |
| 45 | Carlosic acid methyl ester | 223.0615 | 17.32 | [M-H_2_O-H]^-^ | 2.1 | C_11_H_14_O_6_ | 122391261 |
| 46 | **Catechin** | 289.0710 | 14.48 | [M-H]^-^ | 2.9 | C_15_H_14_O_6_ | 9064 |
| 47 | Choline | 104.1066 | 6.46 | [M+H]^+^ | 1.0 | C_5_H_13_NO | 305 |
| 48 | Choline O-Sulfate | 184.0627 | 6.73 | [M+H]^+^ | 4.5 | C_5_H_13_NO_4_S | 486 |
| 49 | Citric acid | 191.0193 | 5.82 | [M-H]^-^ | 2.6 | C_6_H_8_O_7_ | 19782904 |
| 50 | Coumarin | 147.0432 | 18.38 | [M+H]^+^ | 3.9 | C_9_H_6_O_2_ | 323 |
| 51 | Cytosine | 112.0498 | 5.18 | [M+H]^+^ | 4.1 | C_4_H_5_N_3_O | 597 |
| 52 | D-1-[(3-Carboxypropyl)amino]-1-deoxyfructose | 266.1233 | 4.85 | [M+H]^+^ | 0.6 | C_10_H_19_NO_7_ | 131752417 |
| 53 | Daucic acid | 203.0189 | 6.08 | [M-H]^-^,  [M+Na-2H]^-^ | 4.4 | C_7_H_8_O_7_ | 5316316 |
| 54 | Deoxyfructosazine | 305.1343 | 5.34 | [M+H]^+^ | 0.8 | C_12_H_20_N_2_O_7_ | 73452 |
| 55 | Digalacturonate | 369.0673 | 4.51 | [M-H]^-^ | 0.6 | C_12_H_18_O_13_ | 439694 |
| 56 | Dihydroactinidiolide | 181.1219 | 23.39 | [M+H]^+^, [M+Na]^+^ | 0.7 | C_11_H_16_O_2_ | 27209 |
| 57 | **Dihydrocaffeic acid** | 181.0501 | 17.72 | [M-H]^-^ | 3.3 | C_9_H_10_O_4_ | 348154 |
| 58 | **Dihydroferulic acid** | 195.0665 | 19.02 | [M-H]^-^ | 0.7 | C_10_H_12_O_4_ | 14340 |
| 59 | Dysolenticin B | 451.3197 | 23.72 | [M+H-H_2_O]^+^, [M+H]^+^ | 1.5 | C_30_H_42_O_3_ | 56601655 |
| 60 | Enicoflavine | 210.0771 | 17.99 | [M-H]^-^ | 0.7 | C_10_H_13_NO_4_ | 5281564 |
| 61 | **Epicatechin** | 289.0715 | 16.80 | [M-H]^-^ | 1.2 | C_15_H_14_O_6_ | 72276 |
| 62 | **Epigallocatechin** | 305.0666 | 13.48 | [M-H]^-^ | 0.5 | C_15_H_14_O_7_ | 72277 |
| 63 | **Ferulic acid** | 193.0500 | 19.54 | [M-H]^-^ | 3.7 | C_10_H_10_O_4_ | 445858 |
| 64 | Folinic acid | 474.1733 | 17.01 | [M+H]^+^ | 0.9 | C_20_H_23_N_7_O_7_ | 6006 |
| 65 | Furaneol 4-(6-malonylglucoside) | 397.0745 | 12.70 | [M+Na-2H]^-^ | 2.7 | C_15_H_20_O_11_ | 131750900 |
| 66 | Gentiopicroside | 379.0999 | 18.91 | [M+Na]^+^ | 0.5 | C_16_H_20_O_9_ | 88708 |
| 67 | Ginkgoic acid | 345.2435 | 26.26 | [M-H]^-^ | 0.2 | C_22_H_34_O_3_ | 5281858 |
| 68 | Ginsenoyne K | 299.1613 | 24.98 | [M+Na]^+^ | 0.8 | C_17_H_24_O_3_ | 15736266 |
| 69 | Glabraoside A | 597.1607 | 22.37 | [M-H]^-^,  [M+Na-2H]^-^ | 1.2 | C_30_H_30_O_13_ | 102393599 |
| 70 | Guanosine | 282.0841 | 7.83 | [M-H]^-^ | 1.3 | C_10_H_13_N_5_O_5_ | 6802 |
| 71 | **Isoferulic acid** | 193.0497 | 20.52 | [M-H]^-^ | 5.2 | C_10_H_10_O_4_ | 736186 |
| 72 | Isovalerylglucuronide | 301.0896 | 10.01 | [M+Na]^+^ | 1.5 | C_11_H_18_O_8_ | 137383 |
| 73 | **Kaempferol** | 285.0404 | 21.96 | [M-H]^-^ | 0.5 | C_15_H_10_O_6_ | 5280863 |
| 74 | Kuwanon Y | 605.1794 | 7.24 | [M+Na]^+^ | 2.4 | C_34_H_30_O_9_ | 14334307 |
| 75 | Kynurenic acid | 190.0491 | 17.62 | [M+H]^+^ | 2.6 | C_10_H_7_NO_3_ | 3845 |
| 76 | L-Arginine | 175.1184 | 4.34 | [M+H]^+^ | 1.5 | C_6_H_14_N_4_O_2_ | 28782 |
| 77 | Linustatin | 410.1663 | 8.22 | [M+H]^+^ | 2.2 | C_16_H_27_NO_11_ | 119301 |
| 78 | Longicamphenylone | 207.1742 | 24.81 | [M+H-H_2_O]^+^, [M+H]^+^, [M+Na]^+^ | 0.7 | C_14_H_22_O | 91747202 |
| 79 | Longifolenaldehyde | 221.1897 | 23.87 | [M+H]^+^ | 0.0 | C_15_H_24_O | 565584 |
| 80 | L-Ribulose | 149.0451 | 5.19 | [M-H]^-^,  [M+FA-H]^-^ | 3.5 | C_5_H_10_O_5_ | 644111 |
| 81 | **Madecassic Acid** | 503.3367 | 23.79 | [M-H]^-^ | 2.3 | C_30_H_48_O_6_ | 73412 |
| 82 | **Madecassoside** | 973.5017 | 21.35 | [M-H]^-^ | 0.3 | C_48_H_78_O_20_ | 91885295 |
| 83 | Malate | 133.0143 | 5.32 | [M-H_2_O-H]^-^,  [M-H]^-^ | 0.2 | C_4_H_6_O_5_ | 20130941 |
| 84 | Mangiferin | 421.0759 | 16.80 | [M-H]^-^ | 4.3 | C_19_H_18_O_11_ | 5281647 |
| 85 | Muramic acid | 234.0967 | 4.65 | [M+H-H_2_O]^+^, [M+H]^+^ | 0.9 | C_9_H_17_NO_7_ | 433580 |
| 86 | N-(1-Deoxy-1-fructosyl)phenylalanine | 328.1393 | 10.27 | [M+H-H_2_O]^+^, [M+H]^+^, [M+Na]^+^ | 1.5 | C_15_H_21_NO_7_ | 101039148 |
| 87 | N1,N5,N10,N14-Tetra-trans-p-coumaroylspermine | 809.3504 | 23.17 | [M+H]^+^, [M+Na]^+^ | 1.8 | C_46_H_50_N_4_O_8_ | 9810941 |
| 88 | N-Acetyl-D-glucosamine | 222.0961 | 5.14 | [M+H]^+^ | 3.8 | C_8_H_15_NO_6_ | 899 |
| 89 | N-Acetyl-L-glutamic acid | 212.0525 | 7.54 | [M+H-H_2_O]^+^, [M+H]^+^, [M+Na]^+^ | 1.0 | C_7_H_11_NO_5_ | 70914 |
| 90 | **Naringin** | 579.1710 | 20.12 | [M-H]^-^ | 1.7 | C_27_H_32_O_14_ | 442428 |
| 91 | Niacin (Nicotinic acid) | 124.0383 | 5.32 | [M+H]^+^ | 5.9 | C_6_H_5_NO_2_ | 938 |
| 92 | Nomilinic acid 17-glucoside | 695.2913 | 20.15 | [M+H-H_2_O]^+^ | 1.6 | C_34_H_48_O_16_ | 444212 |
| 93 | Palmitic acid | 255.2323 | 25.44 | [M-H]^-^ | 2.8 | C_16_H_32_O_2_ | 985 |
| 94 | Pantothenic Acid | 220.1176 | 10.51 | [M+H]^+^, [M+Na]^+^ | 0.3 | C_9_H_17_NO_5_ | 6613 |
| 95 | Pelargonidin 3-O-glucoside | 431.0967 | 22.47 | [M-H]^-^ | 4.0 | C_21_H_20_O_10_ | 443648 |
| 96 | Phlorin | 311.0739 | 14.27 | [M+H]^+^, [M+Na]^+^ | 1.2 | C_12_H_16_O_8_ | 476785 |
| 97 | Phosphocholine | 184.0727 | 5.27 | [M+H]^+^ | 1.9 | C_5_H_14_NO_4_P | 1014 |
| 98 | Purgic acid B,(-)-Purgic acid B | 1179.5675 | 22.81 | [M-H]^-^ | 2.0 | C_52_H_92_O_29_ | 16091605 |
| 99 | **Quercetin** | 301.0349 | 21.10 | [M-H]^-^ | 1.8 | C_15_H_10_O_7_ | 5280343 |
| 100 | Quercetin 3-(6'-acetylglucoside) | 505.0996 | 20.30 | [M-H]^-^ | 1.5 | C_23_H_22_O_13_ | 44259187 |
| 101 | Quercetin 3-O-glucoside | 463.0884 | 18.77 | [M-H]^-^ | 0.3 | C_21_H_20_O_12_ | 5280804 |
| 102 | **Rutin** | 609.1465 | 18.88 | [M-H]^-^ | 0.5 | C_27_H_30_O_16_ | 5280805 |
| 103 | Sambacin | 563.2089 | 19.43 | [M+Na]^+^ | 1.4 | C_26_H_36_O_12_ | 131752486 |
| 104 | shanzhiside | 391.1215 | 14.13 | [M-H]^-^ | 8.1 | C_16_H_24_O_11_ | 11948668 |
| 105 | Soyacerebroside I | 758.5428 | 26.17 | [M-H]^-^,  [M+FA-H]^-^ | 0.9 | C_40_H_75_NO_9_ | 131751281 |
| 106 | Stachyose | 689.2104 | 4.58 | [M+Na]^+^ | 0.7 | C_24_H_42_O_21_ | 439531 |
| 107 | Succinate | 117.0195 | 7.39 | [M-H]^-^ | 0.8 | C_4_H_6_O_4_ | 1110 |
| 108 | Succinoadenosine | 384.1156 | 11.33 | [M+H]^+^ | 2.3 | C_14_H_17_N_5_O_8_ | 126969142 |
| 109 | Succinyl-L-proline | 238.0706 | 7.31 | [M+Na]^+^ | 7.3 | C_9_H_13_NO_5_ | 194156 |
| 110 | Swertiamarin | 395.0978 | 17.90 | [M+Na-2H]^-^ | 3.8 | C_16_H_22_O_10_ | 442435 |
| 111 | Tetradecanedioic acid | 279.1623 | 23.65 | [M-H]^-^,  [M+Na-2H]^-^ | 8.3 | C_14_H_26_O_4_ | 13185 |
| 112 | Traumatic Acid | 227.1285 | 22.67 | [M-H]^-^ | 2.0 | C_12_H_20_O_4_ | 5283028 |
| 113 | Tropic acid | 165.0551 | 17.88 | [M-H]^-^ | 4.2 | C_9_H_10_O_3_ | 10726 |
| 114 | Tsangane L 3-glucoside | 395.2039 | 22.28 | [M+Na-2H]^-^ | 3.9 | C_19_H_34_O_7_ | 73981648 |
| 115 | Uric acid | 169.0344 | 5.66 | [M+H]^+^ | 5.6 | C_5_H_4_N_4_O_3_ | 1175 |
| 116 | Vincosamide | 499.2067 | 20.70 | [M+H]^+^ | 1.0 | C_26_H_30_N_2_O_8_ | 10163855 |
| 117 | Xanthurenic acid | 206.0439 | 14.77 | [M+H]^+^ | 2.9 | C_10_H_7_NO_4_ | 5699 |

^a^ Matched with Table S7

^b^ Neutral formula obtained for annotated compounds

^c^ **Bold** - Compounds confirmed using authentic standards

**Table S2.** Linear regression functions calculated for 24 precursor ions. Calibration curves were stablished in negative ion mode with correlation factor r > 0.990. Calibration curves were prepared from mixed standard solutions containing analytical blanks, 0.005, 0.01, 0.05, 0.10, 0.50, 1.00, 5.0 and 10.0 mg/L. Compounds are sorted by retention time.

| **Compound** | **[M-H]- (exact mass), XIC** | **Regression function** | **r** |
| --- | --- | --- | --- |
| 5-O-Caffeoylquinic acid | 353.0875 | A^a^=(4.082 C^b^ + 0.0502)*10^6 | 0.9999 |
| Epigallocatechin | 305.0666 | A=(2.745 C - 0.0063)*10^6 | 0.9999 |
| Catechin | 289.0710 | A=(3.514 C + 0.0954)*10^6 | 0.9990 |
| Dihydrocaffeic acid | 181.0511 | A=(2.661 C + 0.0249)*10^6 | 0.9998 |
| 4-O-Caffeoylquinic acid | 353.0872 | A=(3.312 C + 0.0331)*10^6 | 0.9998 |
| 3-O-Caffeoylquinic acid | 353.0869 | A=(4.072 C + 0.0682)*10^6 | 0.9994 |
| Caffeic acid | 179.0349 | A=(4.516 C + 0.0664)*10^6 | 0.9996 |
| Epicatechin | 289.0715 | A=(3.524 C + 0.0731)*10^6 | 0.9994 |
| 1,5-Dicaffeoylquinic acid | 515.1189 | A=(9.217 C + 0.0927)*10^6 | 0.9999 |
| 1,3-Dicaffeoylquinic acid | 515.1189 | A=(9.217 C + 0.0927)*10^6 | 0.9999 |
| Rutin | 609.1452 | A=(5.673 C + 0.0103)*10^6 | 0.9999 |
| Dihydroferulic acid | 195.0665 | A=(3.234 C + 0.0581)*10^6 | 0.9984 |
| 3,4-Dicaffeoylquinic acid | 515.1190 | A=(3.451 C + 0.0377)*10^6 | 1.0000 |
| 3,5-Dicaffeoylquinic acid | 515.1190 | A=(3.045 C + 0.0300)*10^6 | 1.0000 |
| Ferulic acid | 193.0510 | A=(1.551 C + 0.0241)*10^6 | 0.9992 |
| 4,5-Dicaffeoylquinic acid | 515.1192 | A=(5.546 C + 0.0570)*10^6 | 0.9999 |
| Naringin | 579.1718 | A=(4.730 C + 0.0510)*10^6 | 0.9999 |
| Isoferulic acid | 193.0510 | A=(0.417 C + 0.0032)*10^6 | 0.9999 |
| Quercetin | 301.0340 | A=(7.879 C + 0.2305)*10^6 | 0.9975 |
| Madecassoside | 973.5018 | A=(0.647 C + 0.0401)*10^6 | 0.9997 |
| Asiaticoside | 957.5087 | A=(0.506 C + 0.0107)*10^6 | 0.9996 |
| Kaempferol | 285.0404 | A=(12.150 C + 0.3562)*10^6 | 0.9916 |
| Madecassic Acid | 503.3370 | A=(1.232 C + 0.3609)*10^6 | 0.9895 |
| Asiatic acid | 487.3428 | A=(1.937 C + 0.1587)*10^6 | 0.9866 |

^a^A: peak area for the quantifier

^b^c: concentration, mg l^–1^.

**SUPPLEMENTAL FIGURES**


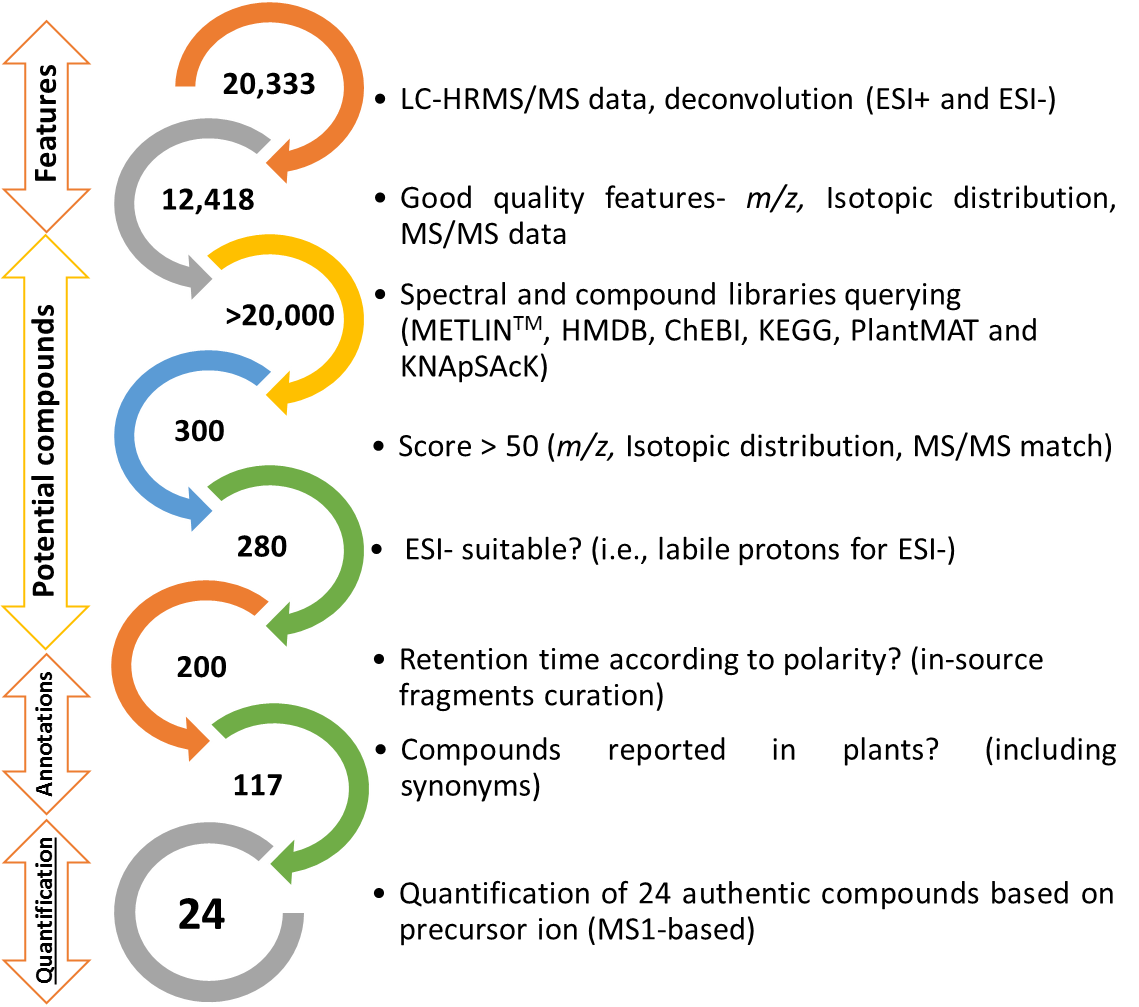


**Figure S1.** Untargeted workflow approach. This workflow yielded 24 identified (L1 annotation) and 93 tentatively assigned (L2) compounds (117 in total). Compound description is detailed in Table S1.


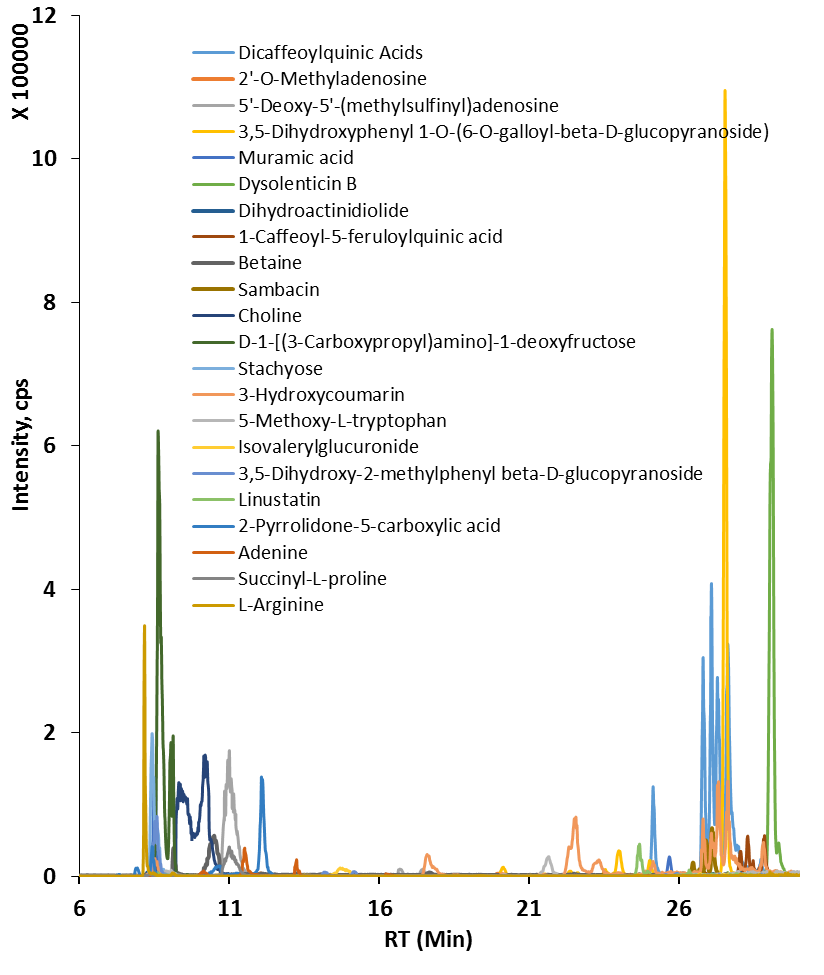


**Figure S2.** Examples of extracted ion chromatograms of compounds detected in *C. asiatica* water extract by data-dependent LC-MS/MS analysis in the positive ionization mode (ESI+). Table S1 compiles retention time and mass spectral data for identified (L1 annotation) and tentatively annotated (L2) compounds including retention time, *m/z*, molecular formula and detected adducts.


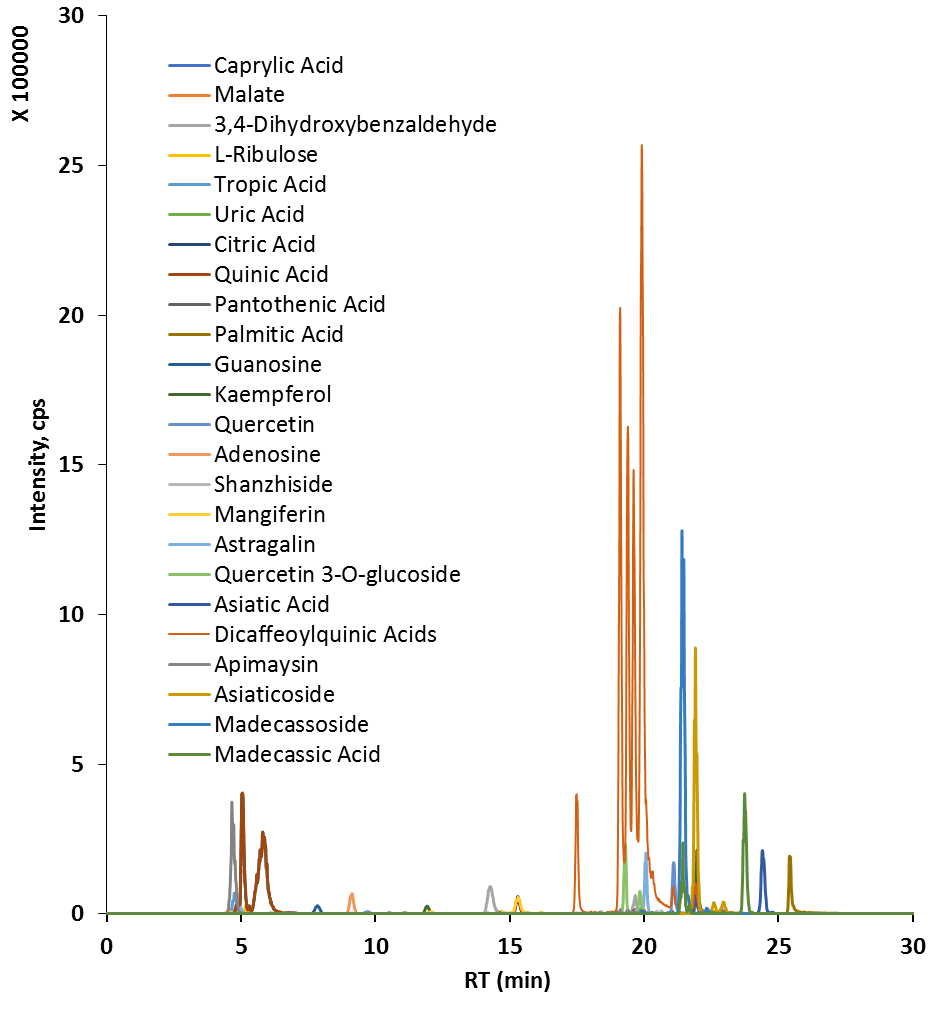


**Figure S3.** Examples of extracted ion chromatograms of compounds detected in *C. asiatica* water extract by data-dependent LC-MS/MS analysis in the negative ionization mode (ESI-). Table S1 compiles retention time and mass spectral data for identified (L1 annotation) and tentatively annotated (L2) compounds including retention time, *m/z*, molecular formula and detected adducts.

**Figure S4.** Chemical structures of compounds for which authentic standards were available and which were selected as marker compounds for quantification. Three mono-caffeoylquinic acids (green), five di-caffeoylquinic acids (magenta), seven flavonoids (purple), five hydroxycinnamic acid derivatives (black) and four triterpenes (blue).


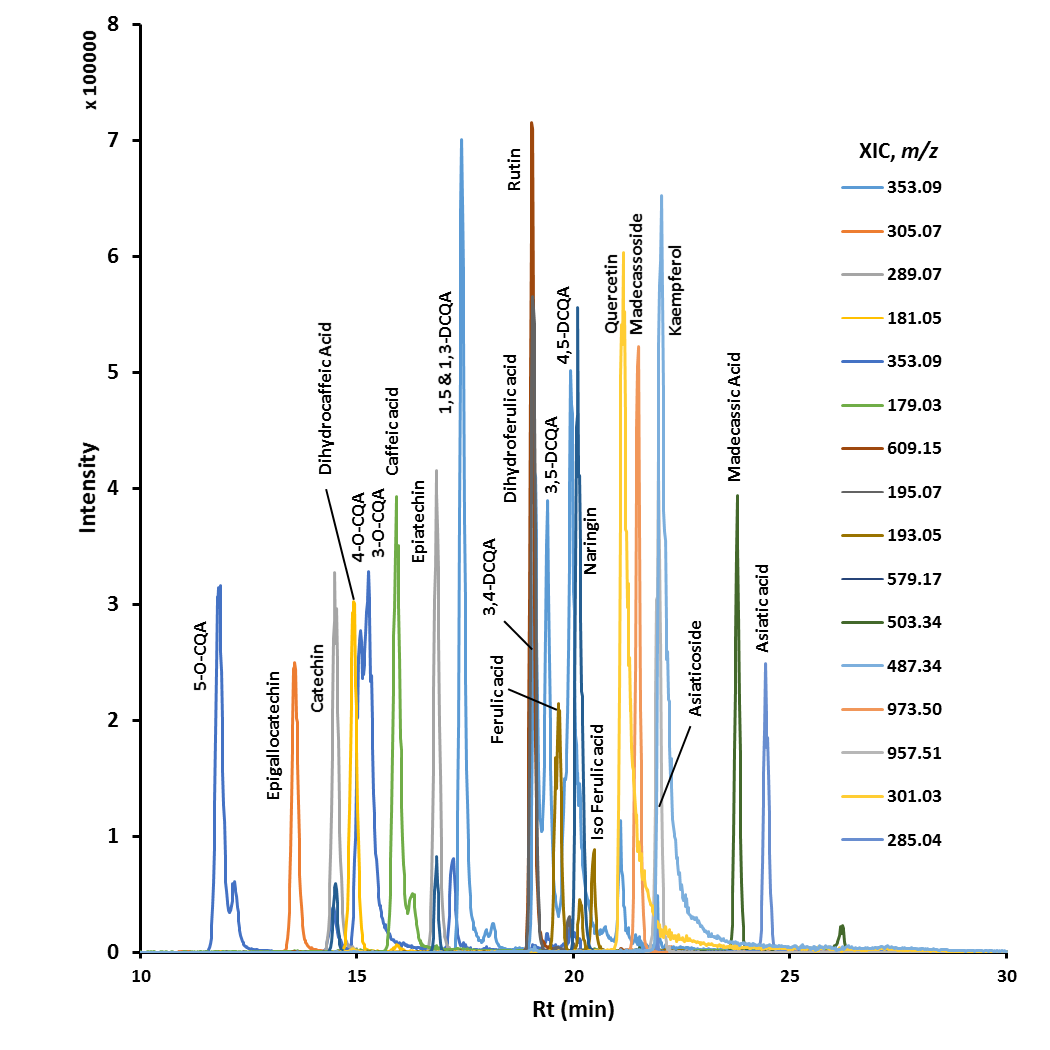


**Figure S5.** Extracted ion chromatograms obtained for the calibration solution containing the 24 selected phytochemical marker compounds (1 mg/L each). Negative ion mode, extracted *m/z* values are indicated in the figure. Analytical parameters are shown in Table 2.


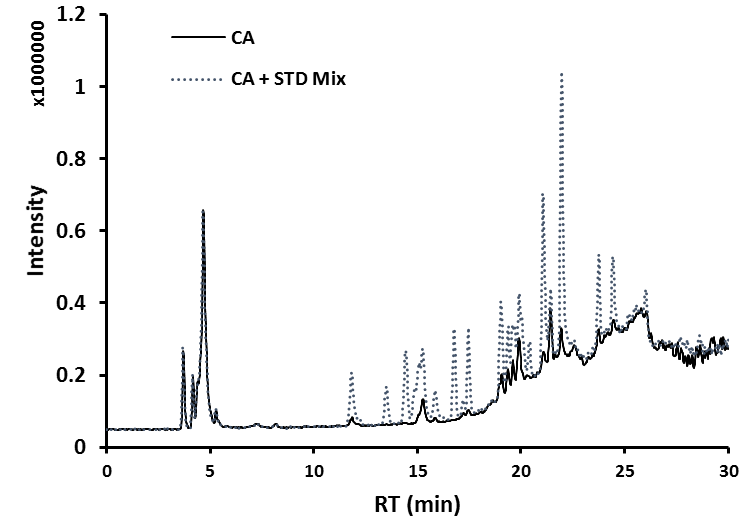


**Figure** **S6.** Standard addition experiment. Total ion chromatogram (TIC) obtained for *C. asiatica* water extract (solid line, 10 µl injection) and same sample after standard addition (dotted line). For the standard addition experiment, 1.0 mL of standard mix containing 1.0 mg/L of each compound was added to 1.0 mL of the pooled CA sample (200 mg/L).

**Figure S7a.** MS/MS spectra of compounds in *C. asiatica* extracts (pooled CA sample) that were assigned tentatively (L2 annotations) by extensive querying and comparison with spectral libraries (including METLIN, our in-house library, ChEBI, and the Human Metabolite Database (HMDB)) using Progenesis QI^TM^ and applying the workflow shown in Figure S1. Red lines were matches against the databases. Eighty-seven compounds that were detected in *C. asiatica* aqueous extracts and tentatively assigned but have not been reported for *C. asiatica* as of to date are denoted with an * in Table 1. The number shown in the spectra matches the entry number # in Table S1. MS/MS scores are indicated in [ ] and were obtained using Progenesis QI. GNPS identifiers are provided in {}.

| 1,4-Dicaffeoylquinic acid, CID: 12358846, in-house library; [68.8]; CCMSLIB00005467925  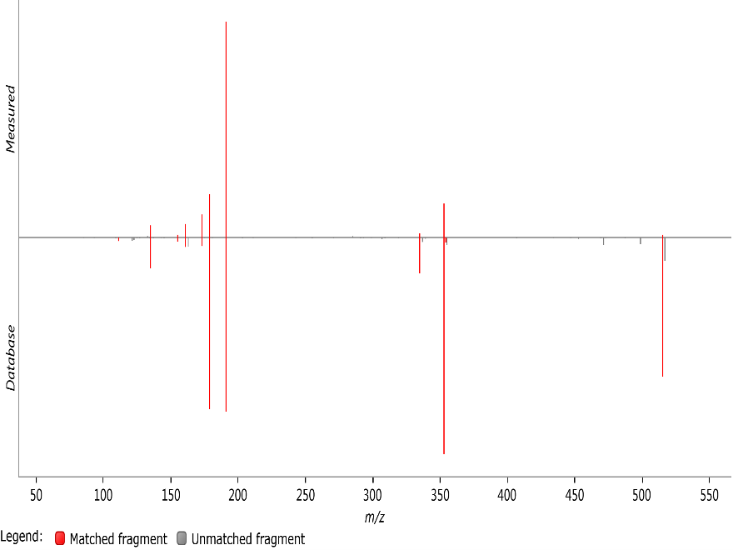  **#2** | 12-Oxodihydrophytodienoic acid, CID: 5716902, ChEBI; [31.0]; CCMSLIB00005467924  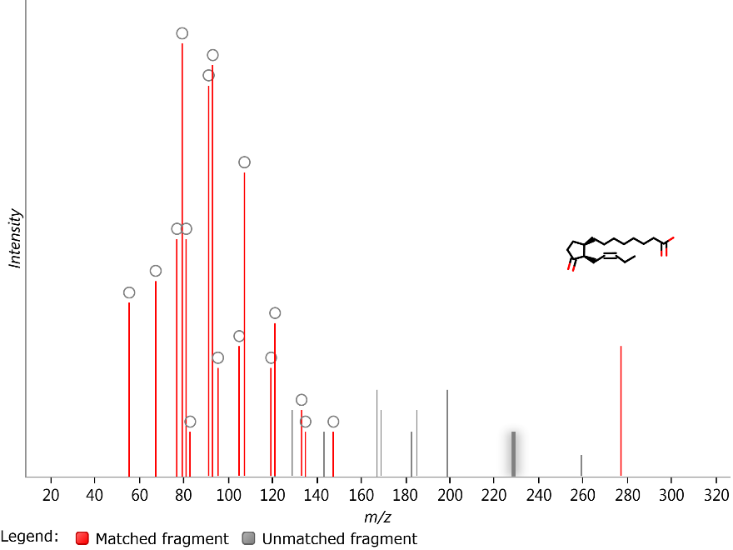  **#4** |
| --- | --- |
| 16-hydroxypalmitic acid, CID: 10466, ChEBI; [86.2]; {CCMSLIB00005467727}  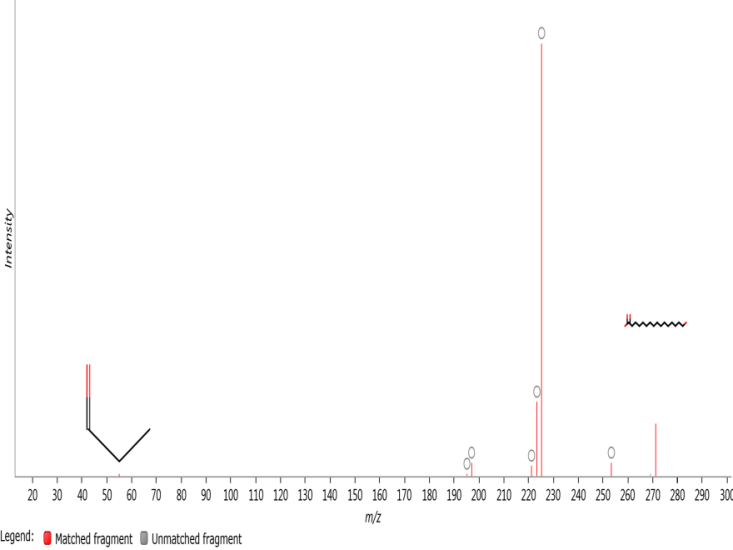  **#5** | 1-beta-D-Glucopyranosyl-L-tryptophan, CID: 11772967, ChEBI; [62.4]; {CCMSLIB00005467655}  **#6**  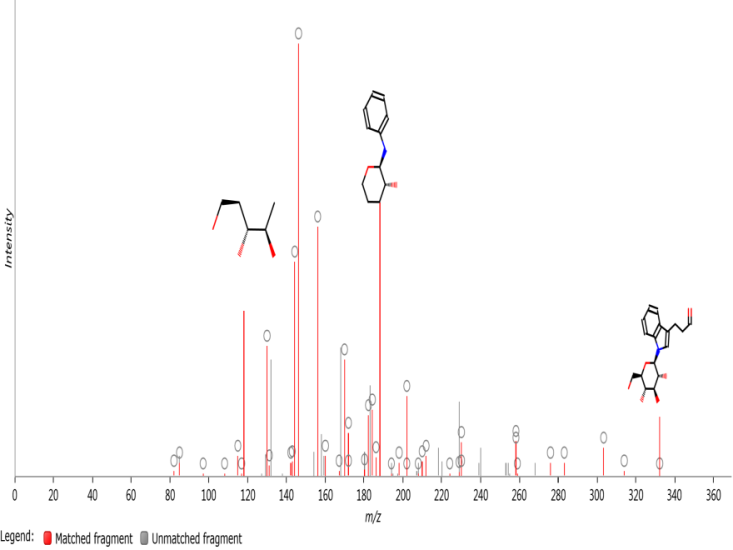 |

| 1-Caffeoyl-5-feruloylquinic acid, CID: 121225501, ChEBI; [49.6]; {CCMSLIB00005467656}  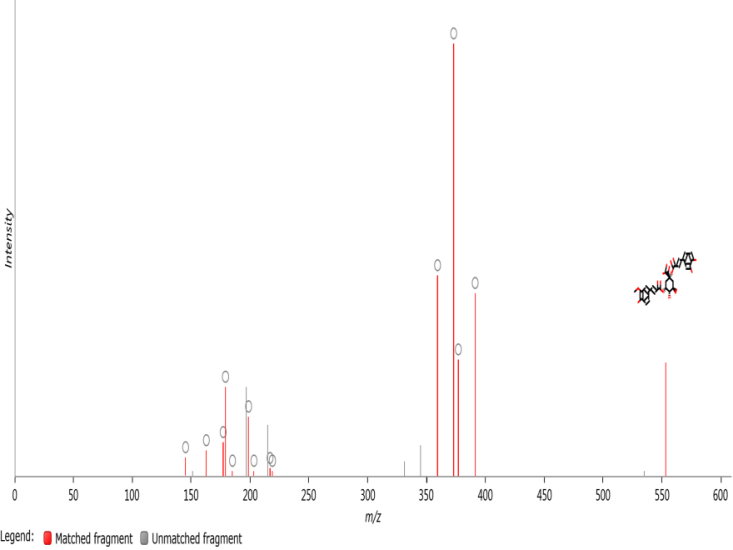  **#7** | | | 2,6-Piperidinedicarboxylic acid, CID: 557515, METLIN; [53.1]; {CCMSLIB00005467657}  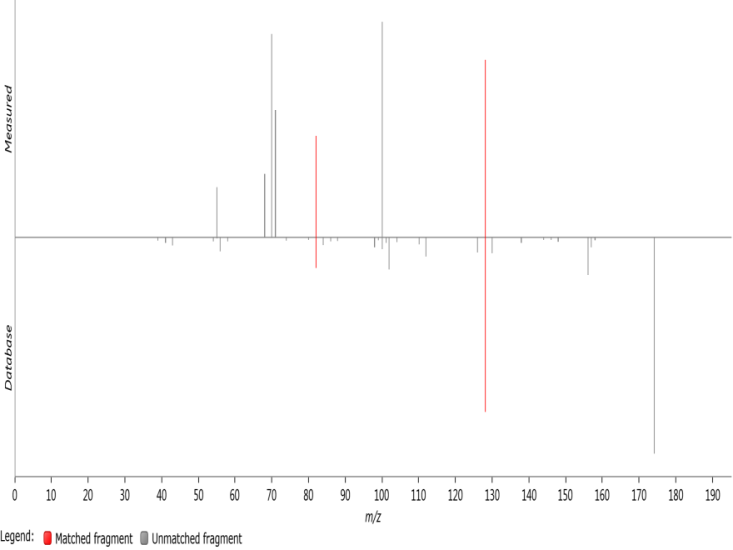  **#8** | |
| --- | --- | --- | --- | --- |
| 26-(2-Glucosyl-6-acetylglucosyl]-1,3,11,22-tetrahydroxyergosta-5,24-dien-26-oate, CID: 131752817, HMDB; [43.5]; {CCMSLIB00005467797}  **#9**  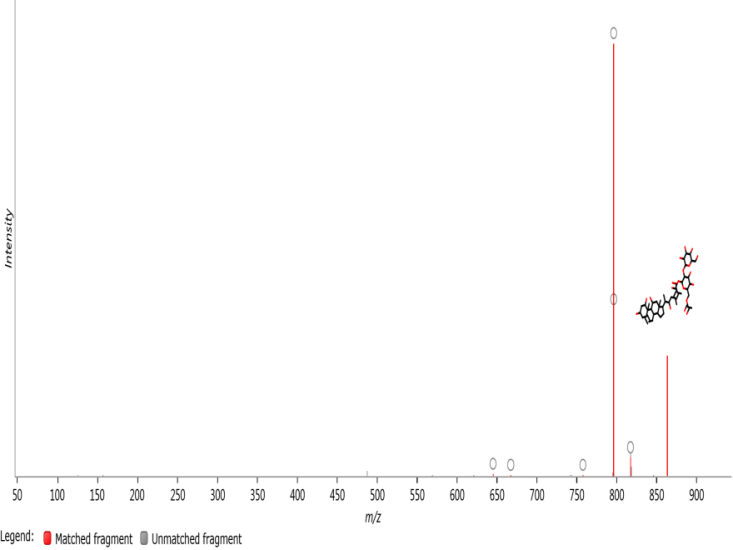 | | | 2'-O-Methyladenosine, CID: 102213, METLIN; [56.2]; {CCMSLIB00005467658}  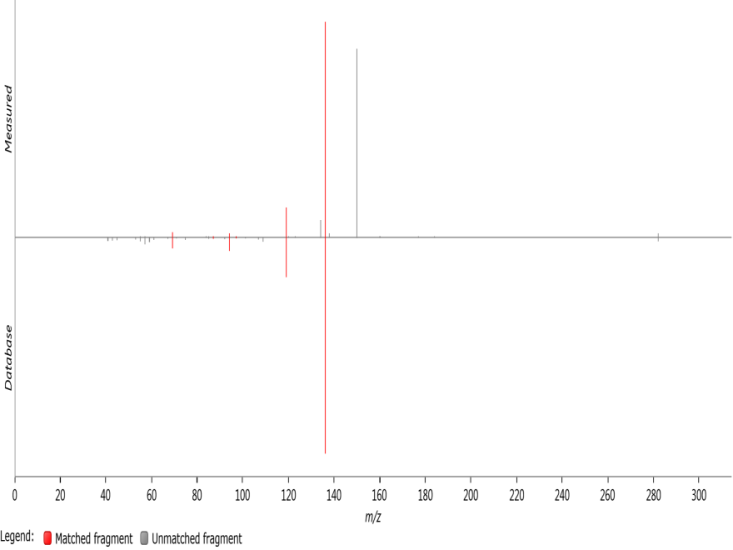  **#10** | |
| 3,4-Dihydroxybenzaldehyde, CID: 8768, ChEBI; [63.5]; {CCMSLIB00005467663}  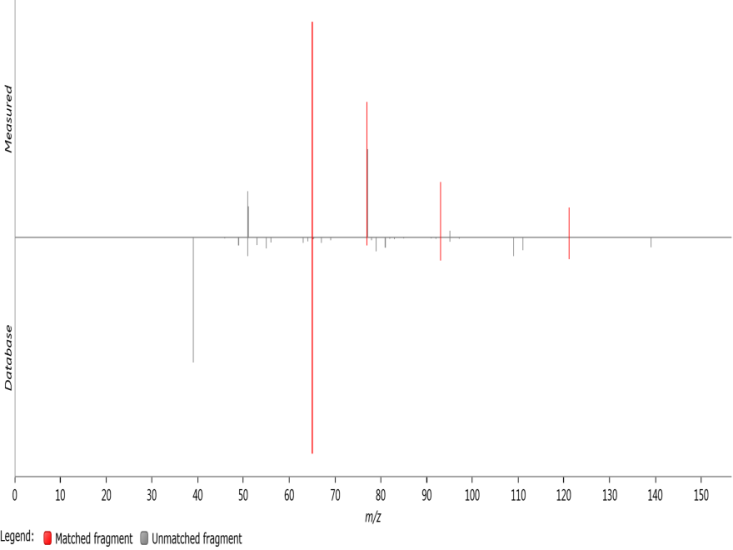  **#14** | | | 3,5-Dihydroxy-2-methylphenyl beta-D-glucopyranoside, CID: 46184089, ChEBI; [59.1]; {CCMSLIB00005467664}  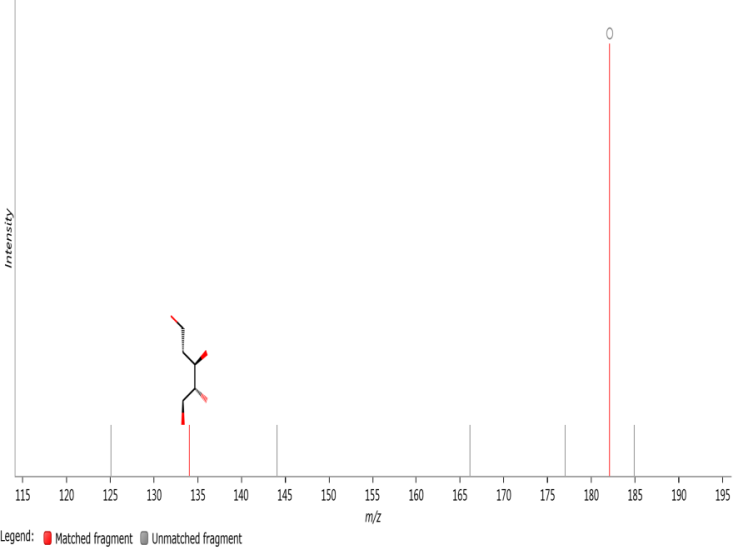  **#16** | |
| 4-Guanidinobutanoic acid, CID: 25200642, METLIN; [79.4]; {CCMSLIB00005467667}  **#21**  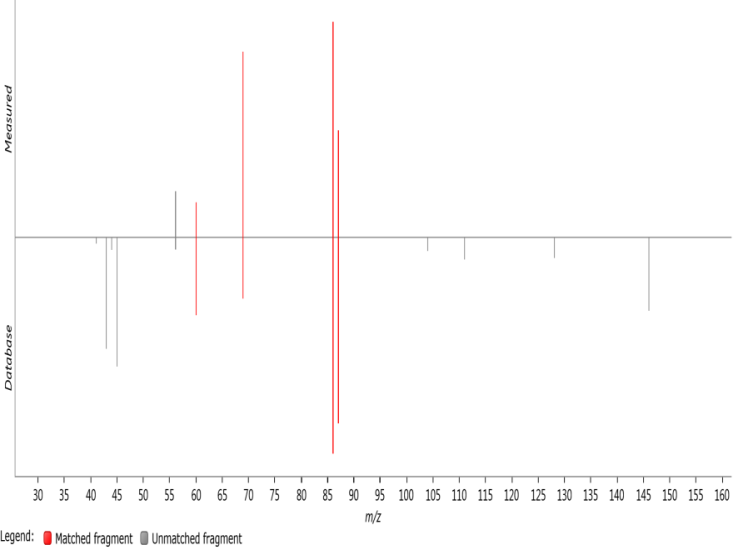 | | | 4-Hydroxybenzaldehyde, CID: 126, METLIN; [78.8]; {CCMSLIB00005467733}  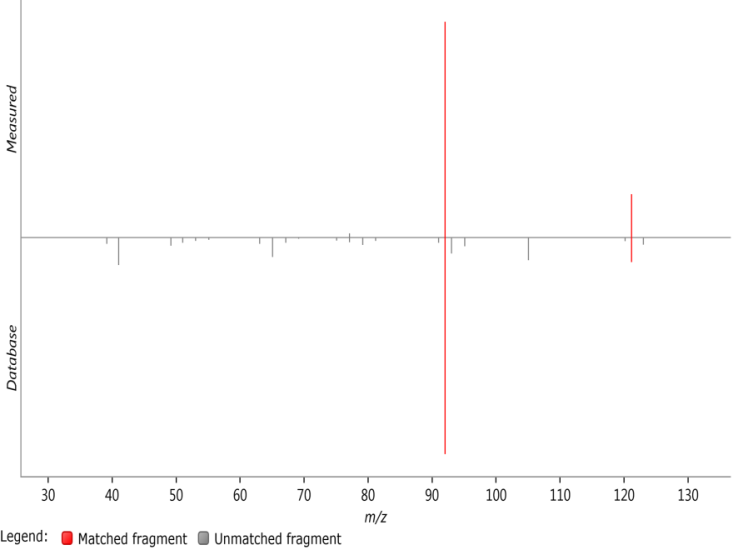  **#22** | |
| 5'-Deoxy-5'-(methylsulfinyl)adenosine, CID: 165114, METLIN; [62.9]; {CCMSLIB00005467668}  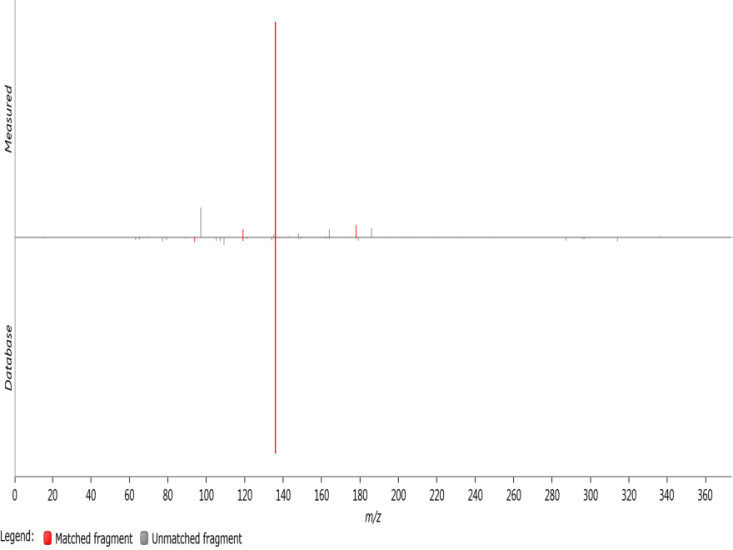  **#24** | | | 5-Methoxy-L-tryptophan, CID: 151018, ChEBI; [58.5]; {CCMSLIB00005467669}  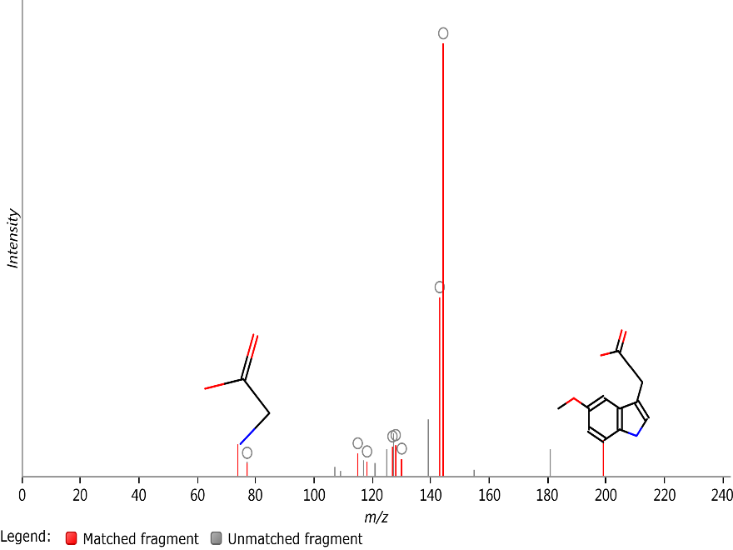  **#25** | |
| 5-Methoxysalicylic acid, CID: 75787, KNApSAcK  ; [69.2]; {CCMSLIB00005467734}  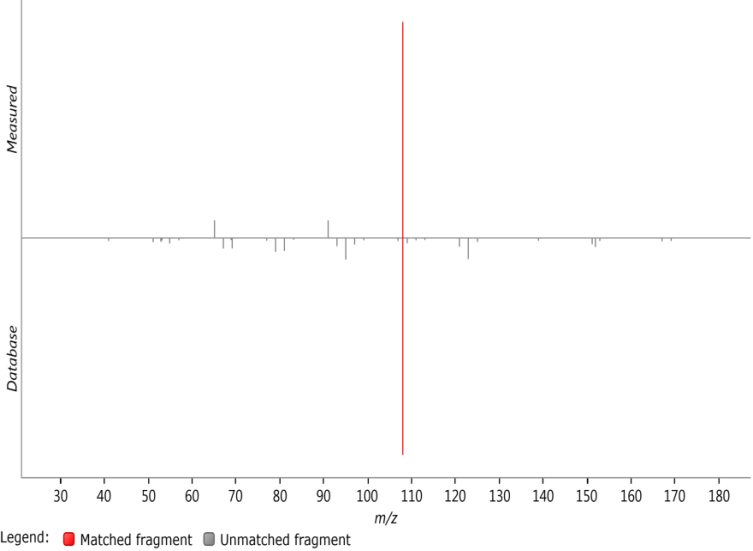  **#26** | | | 6-Amino-9H-purine-9-propanoic acid, CID: 255450, METLIN; [48.8]; {CCMSLIB00005467673}  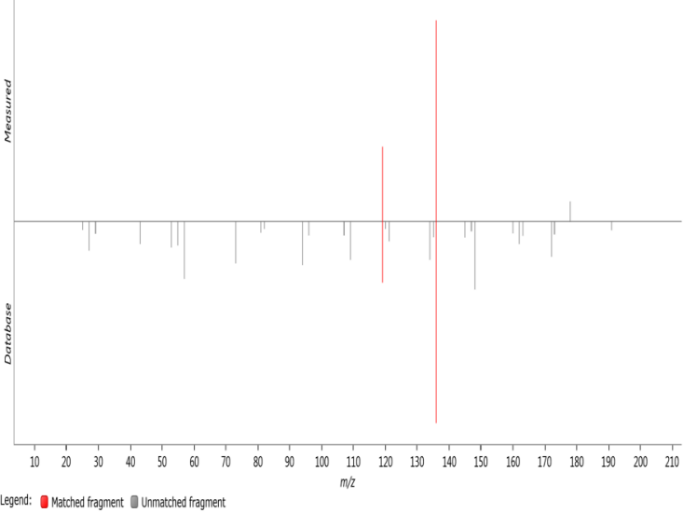  **#28** | |
| 6-Docosenamide, CID: 44584605, ChEBI; [98.7]; {CCMSLIB00005467675}  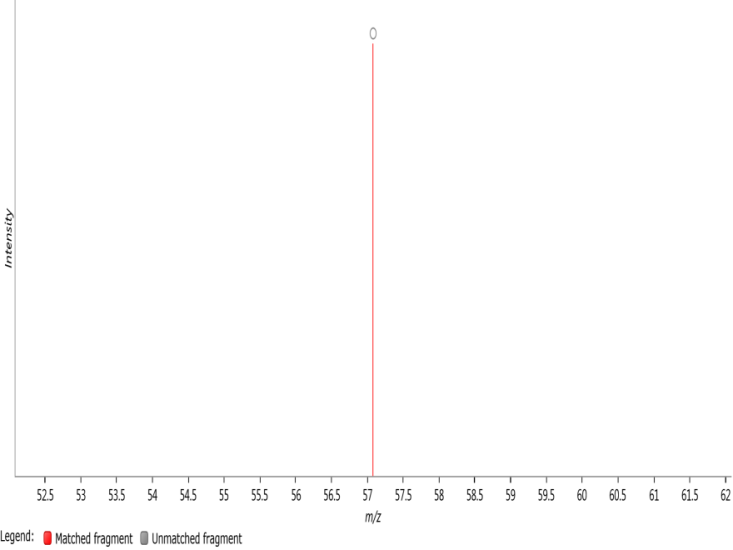  **#30** | 6-Oxo-2-piperidinecarboxylic acid, CID: 3014237, ChEBI; [89.9]; {CCMSLIB00005467676}  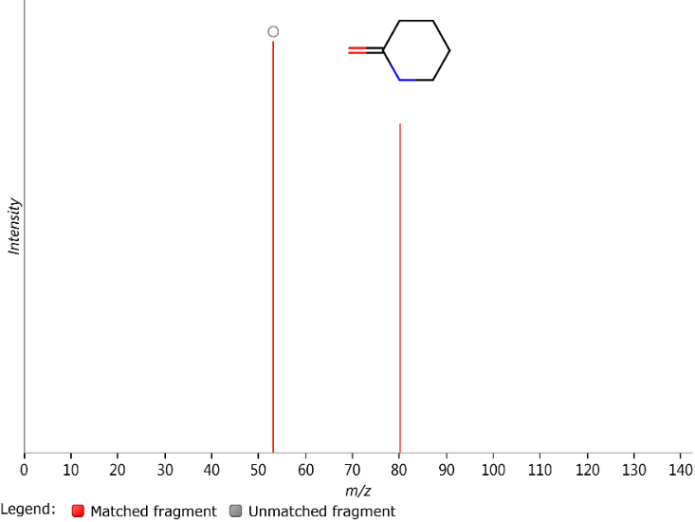  **#31** | | | |
| 8-Acetoxy-4'-methoxypinoresinol 4-glucoside, CID: 73830447, HMDB; [52.9]; {CCMSLIB00005467677}  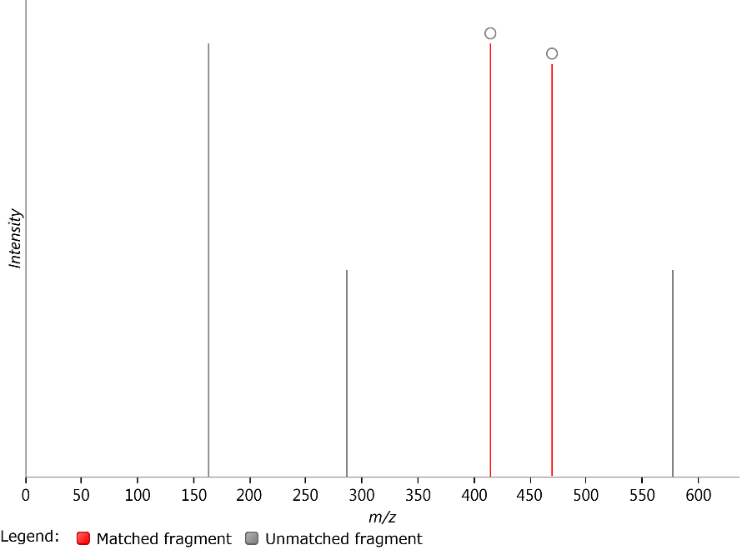  **#32** | Adenine, CID: 190, METLIN; [88.6]; {CCMSLIB00005467678}  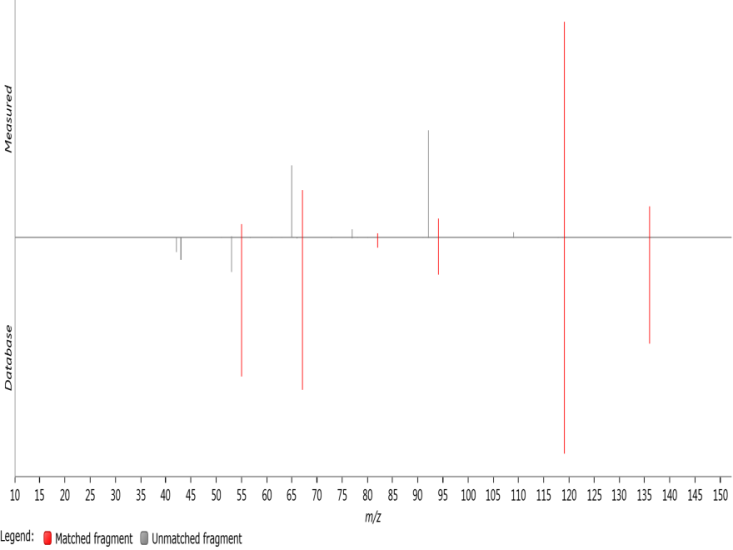  **#33** | | | |
| Adenosine, CID: 60961, in-house library; [91.6]; {CCMSLIB00005467736}  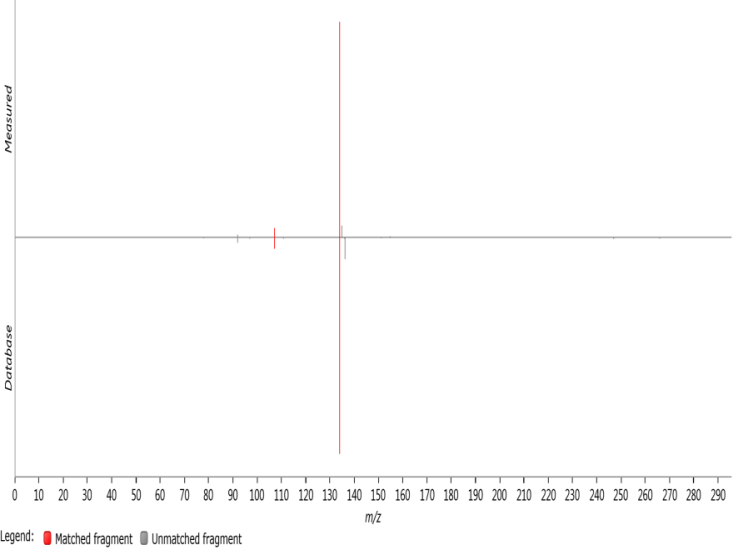  **#34** | Aesculin, CID: 5281417, METLIN; [69.3]; {CCMSLIB00005467679}  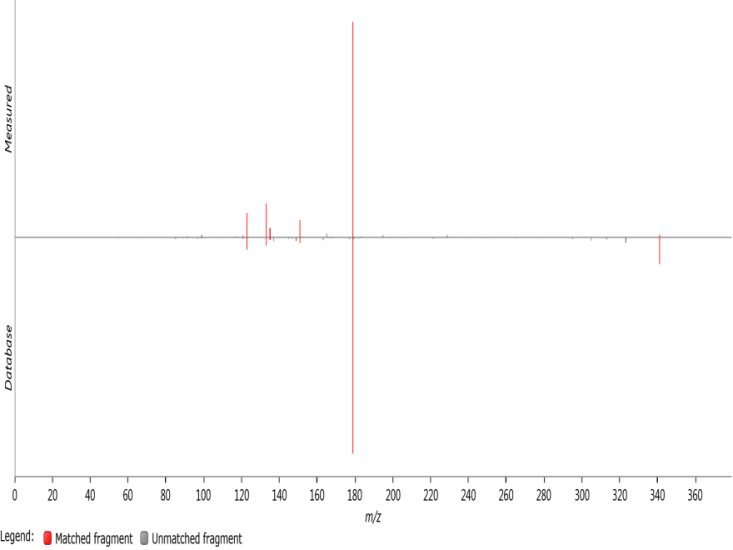  **#35** | | | |
| Apimaysin, CID: 101920411, HMDB; [45.4]; {CCMSLIB00005467737}  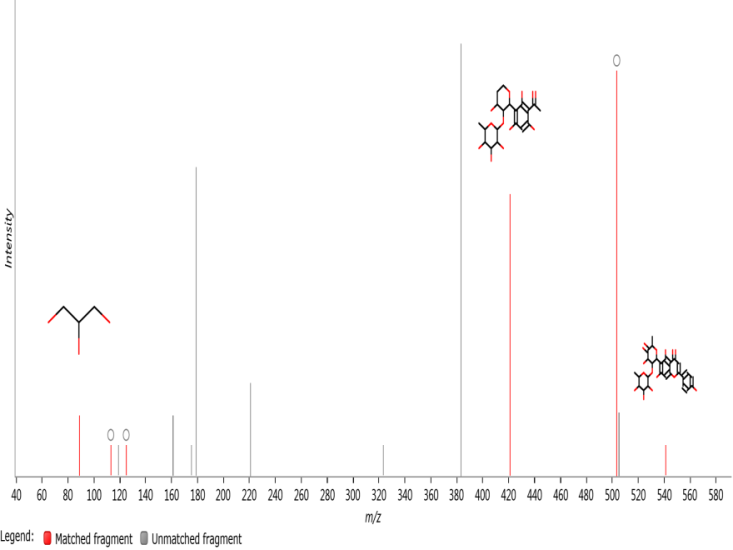  **#36** | Betaine, CID: 247, METLIN; [66.5]; {CCMSLIB00005467650}  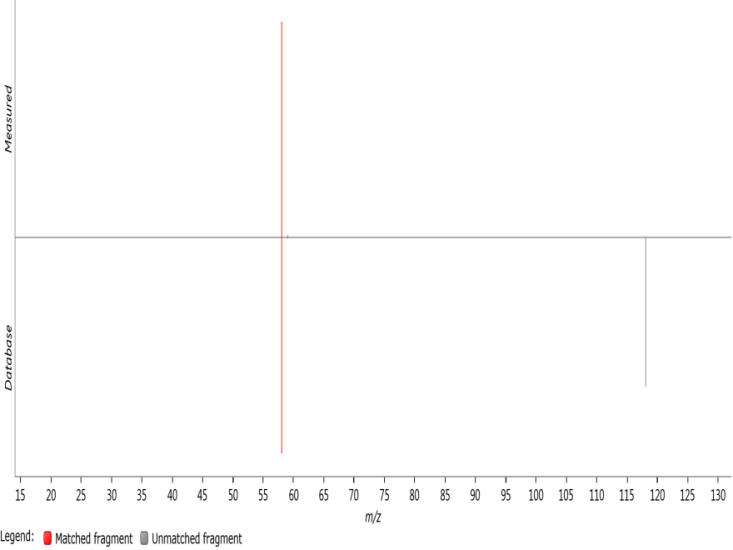  **#41** | | | |
| cAMP, CID: 6076, METLIN; [59.7]; {CCMSLIB00005467743}  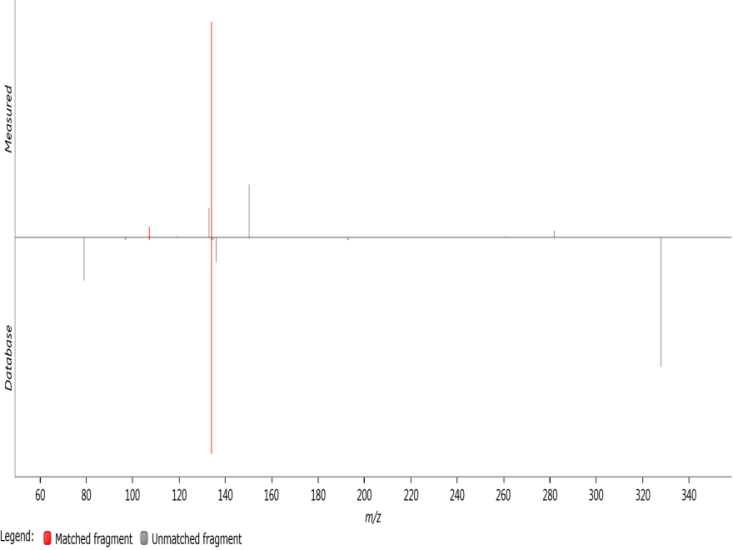  **#43** | Caprylic acid, matched with in-house library, CID: 379, HMDB; [90.2]; {CCMSLIB00005467744}  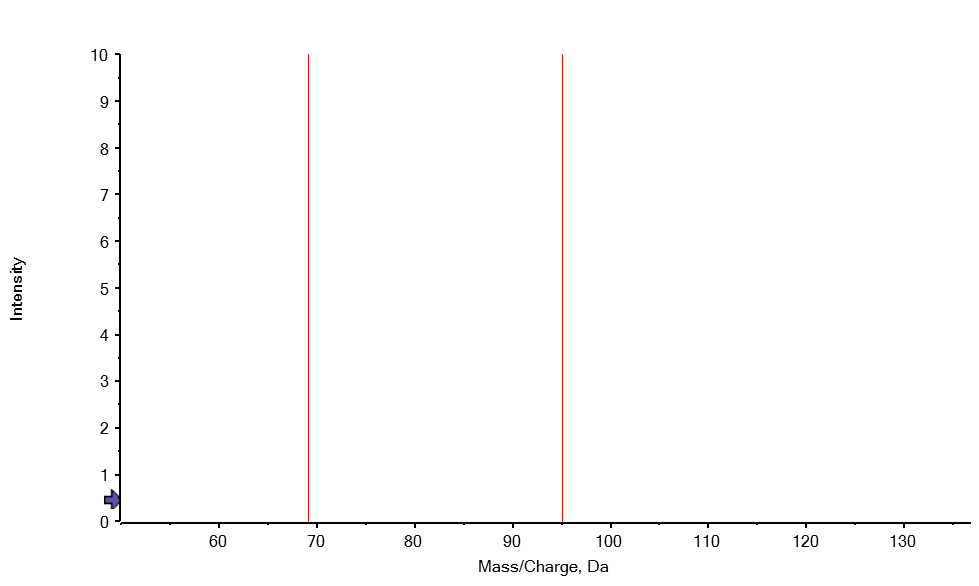  **#44** | | | |
| Carlosic acid methyl ester, CID: 122391261, ChEBI; [70.8]; {CCMSLIB00005467745}  **#45**  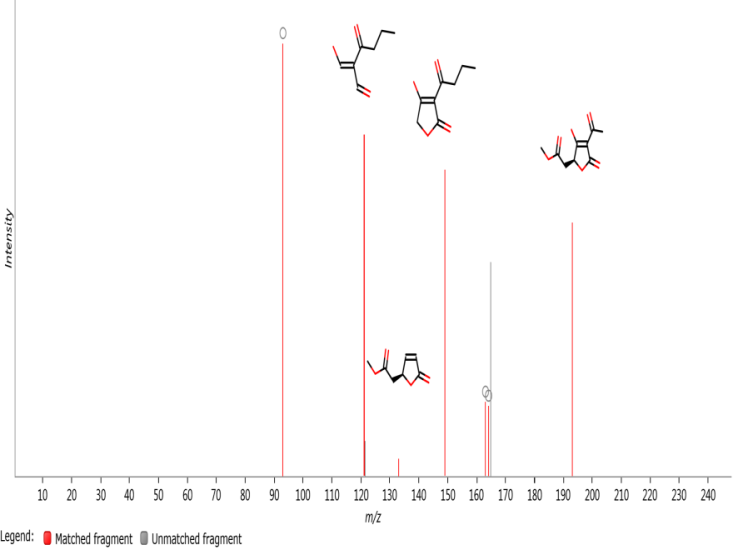 | Choline, CID: 305, METLIN; [75.6]; {CCMSLIB00005467680}  **#47**  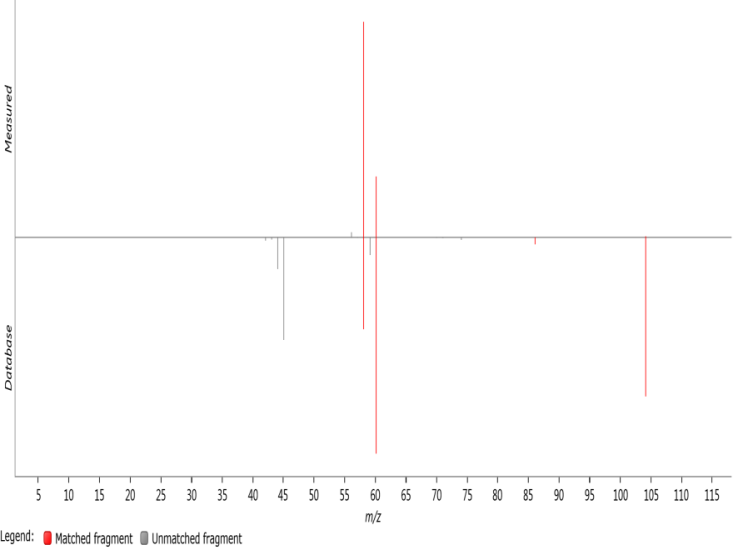 | | | |
| Choline O-Sulfate, CID: 486, ChEBI; [95.1]; {CCMSLIB00005467681}  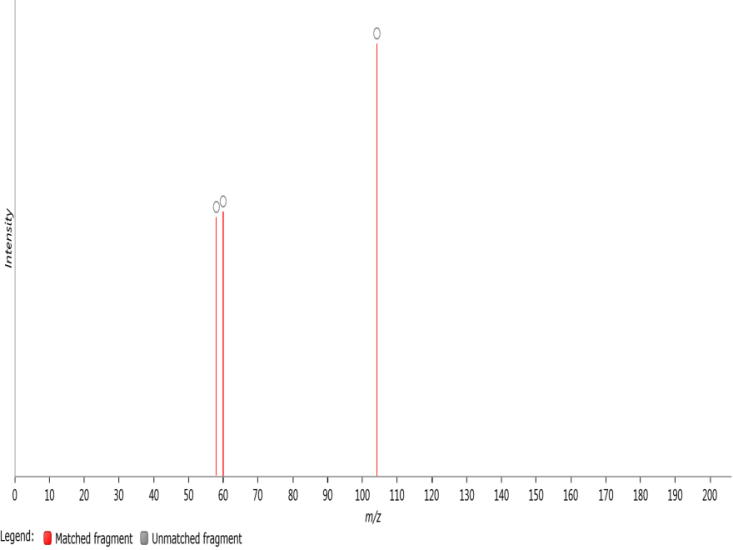  **#48**  **#51** | Citric acid, matched with in-house library, CID: 19782904, METLIN; [69.5]; {CCMSLIB00005467746}  **#49**  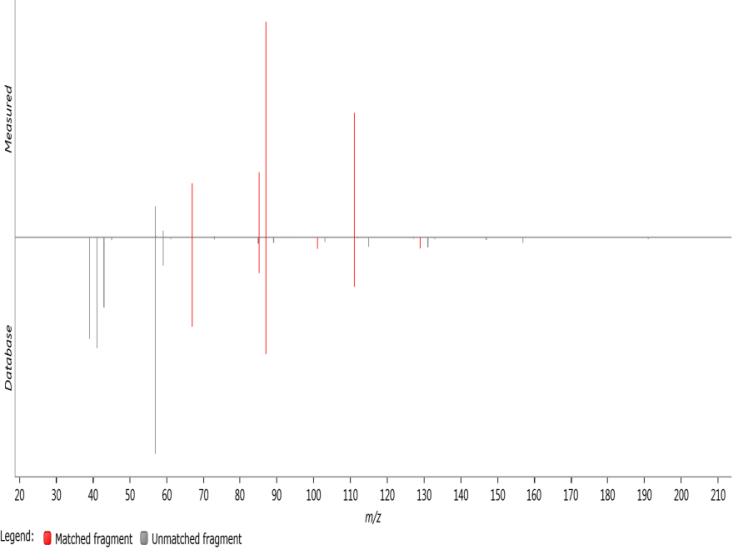 | | | |
| Cytosine, CID: 597, METLIN; [92.3]; {CCMSLIB00005467683}  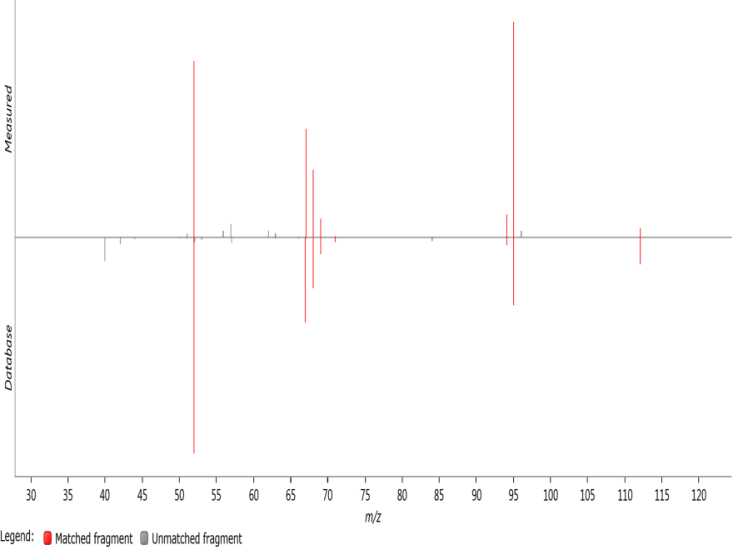 | D-1-[(3-Carboxypropyl)amino]-1-deoxyfructose, CID: 131752417, HMDB; [53.6]; {CCMSLIB00005467684}  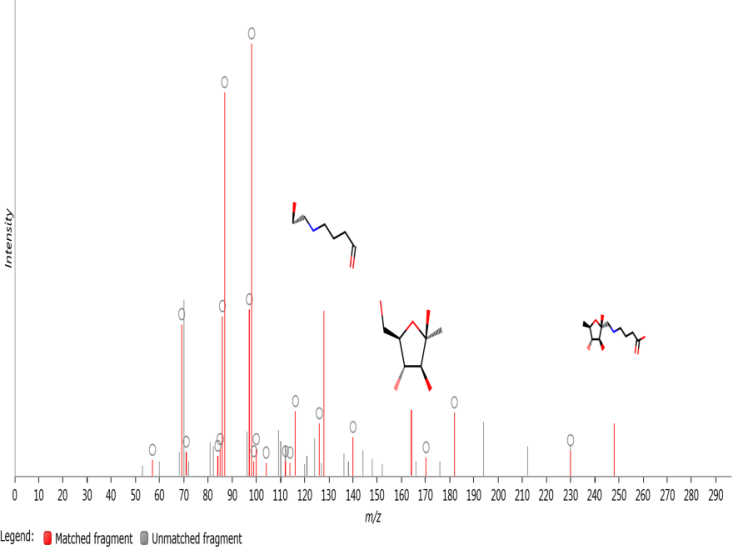  **#52** | | | |
| Daucic acid, CID: 5316316, HMDB; [47.7]; {CCMSLIB00005467747}  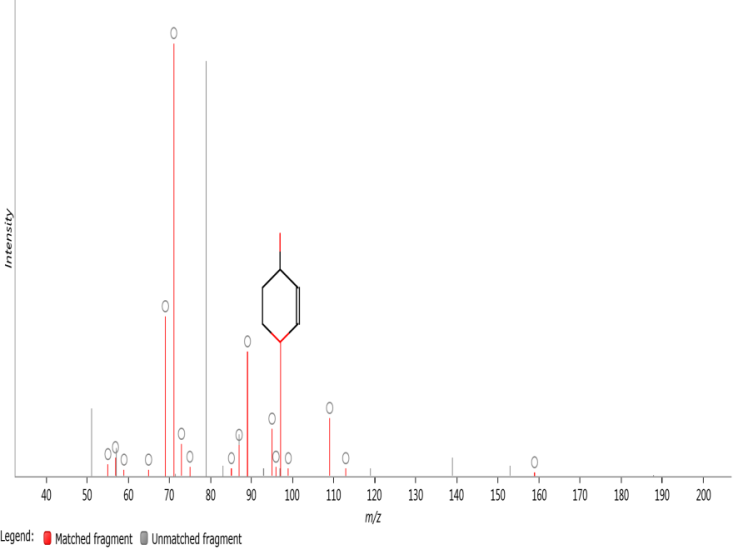  **#53** | Deoxyfructosazine, CID: 73452, HMDB; [60.4] ; {CCMSLIB00005467697}  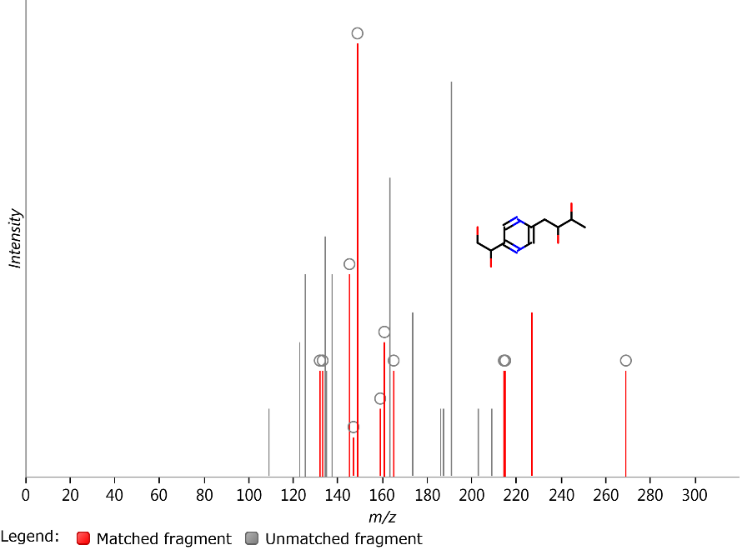  **#54** | | | |
| Digalacturonate, CID: 439694, METLIN; [40.6]; {CCMSLIB00005467748}  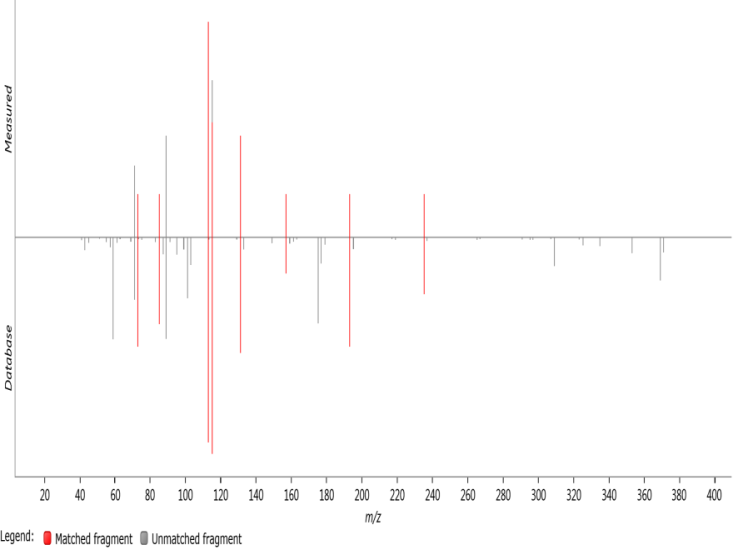  **#55** | | Dihydroactinidiolide, CID: 27209, METLIN; [67.2]; {CCMSLIB00005467698}  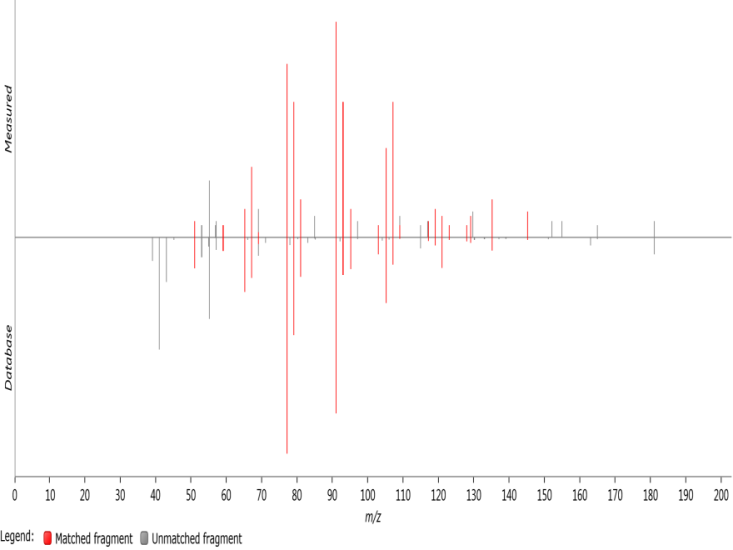  **#56** | | |
| Dihydrocaffeic acid, CID: 348154, ChEBI; [65.1]  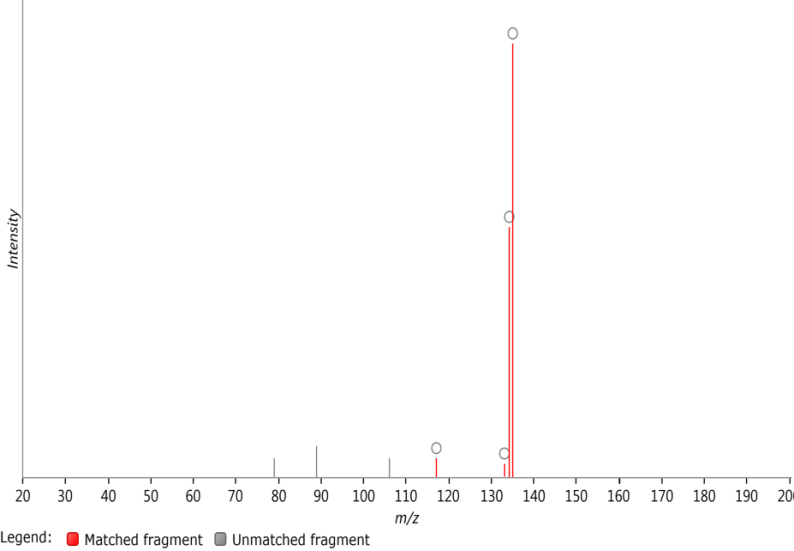  **#57** | | Dihydroferulic acid, matched with in-house library, CID: 14340; [55]  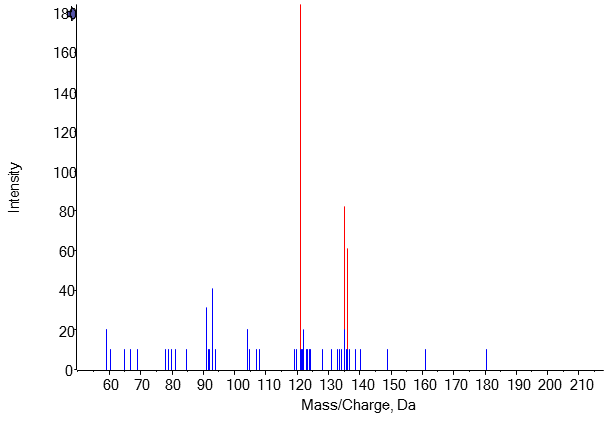  **#58** | | |
| Dysolenticin B, CID: 56601655, ChEBI; [40.4]; {CCMSLIB00005467699}  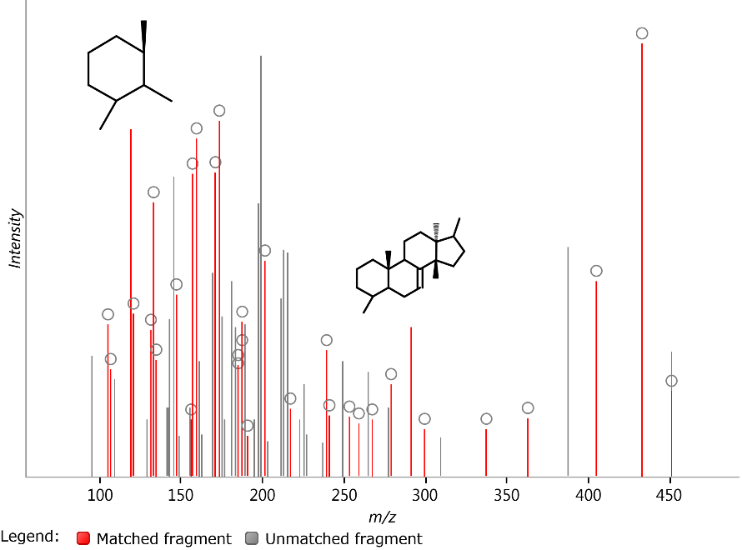  **#59** | | Enicoflavine, CID: 5281564, ChEBI; [71.7]; {CCMSLIB00005467749}  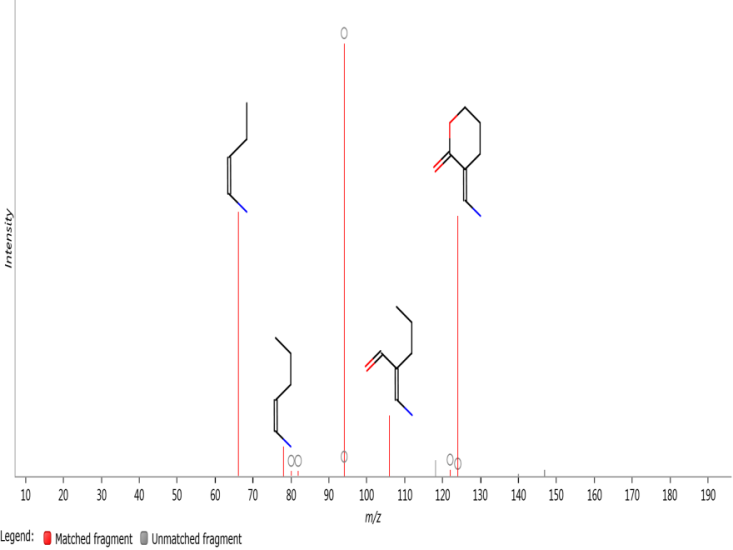  **#60** | | |
| Epigallocatechin, CID: 72277, in-house library; [80.5]  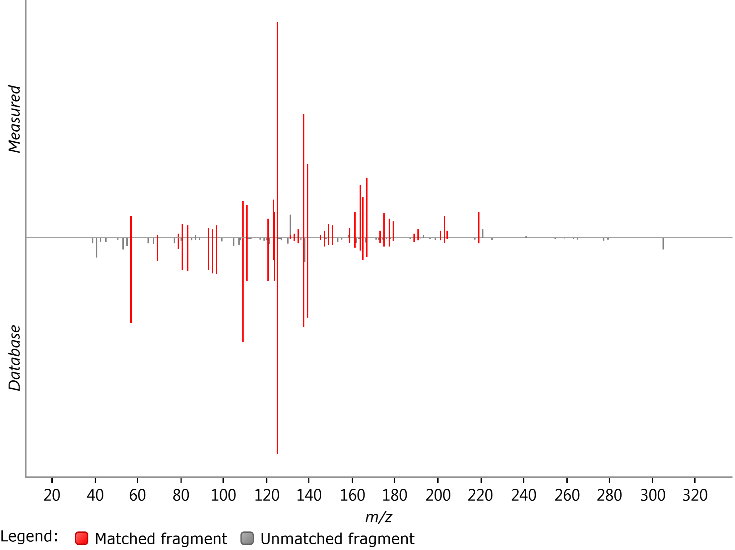  **#62** | | Folinic acid, CID: 6006, in-house library; [50.1]; {CCMSLIB00005467700}  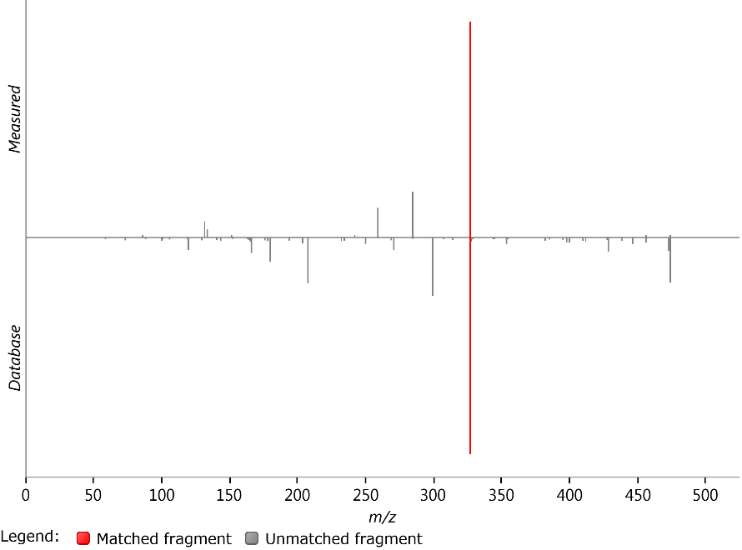  **#64** | | |
| Furaneol 4-(6-malonylglucoside), CID: 131750900, HMDB; [44.1]; ; {CCMSLIB00005467750}  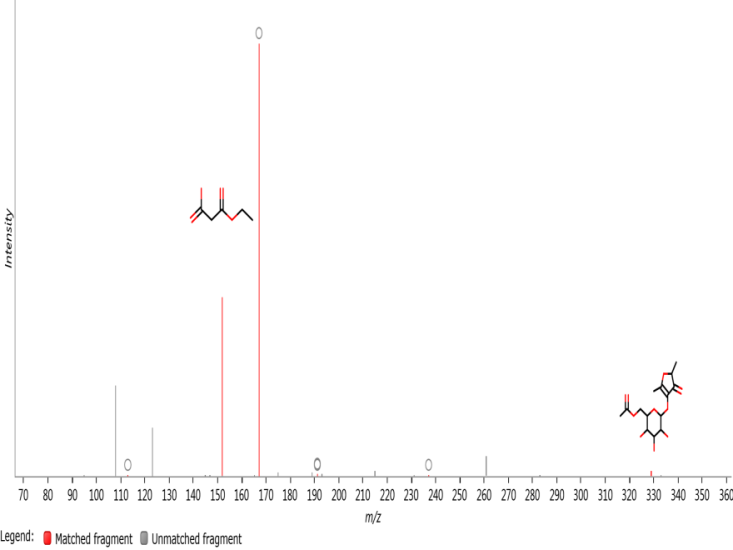  **#65** | | Gentiopicroside, CID: 88708, ChEBI; [34.4]; {CCMSLIB00005467701}  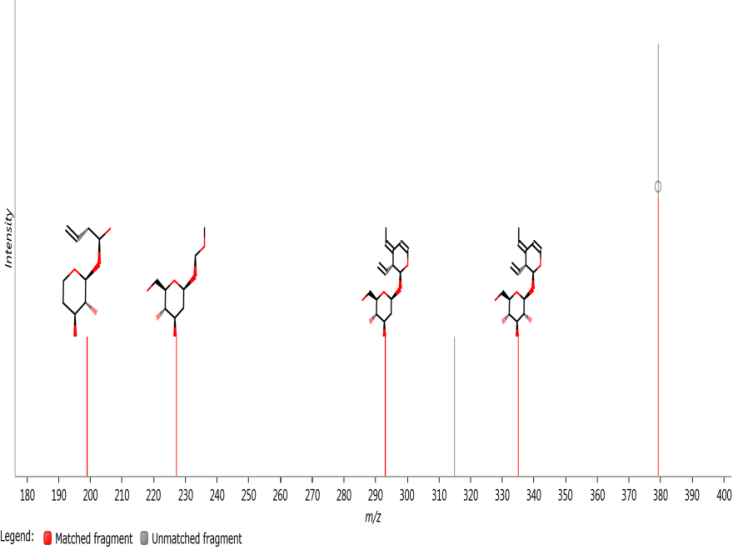  **#66** | | |
| Ginkgoic acid, CID: 5281858, METLIN; [71]; {CCMSLIB00005467751}  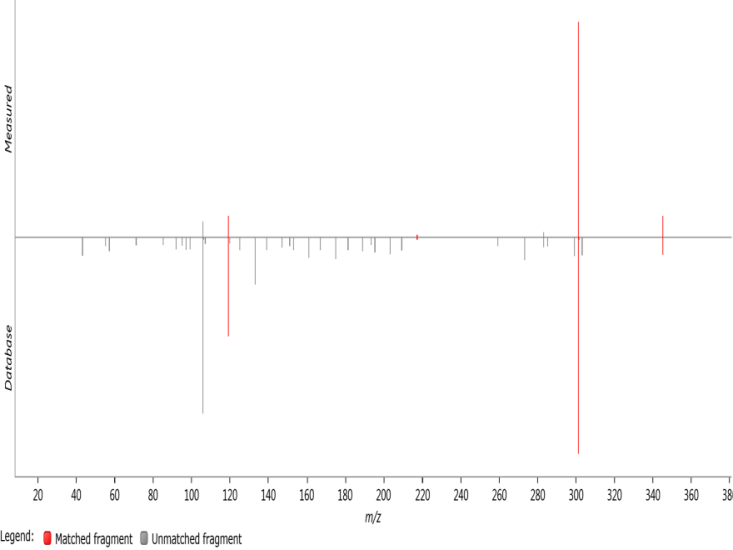  **#67** | | Ginsenoyne K, CID: 15736266, HMDB; [73.3]; {CCMSLIB00005467702}  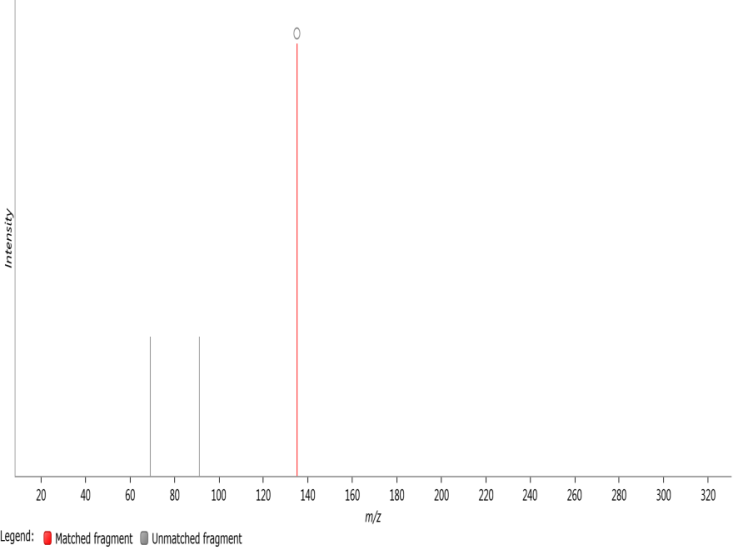  **#68** | | |
| Glabraoside A, CID: 102393599, ChEBI; [36.3]; {CCMSLIB00005467752}  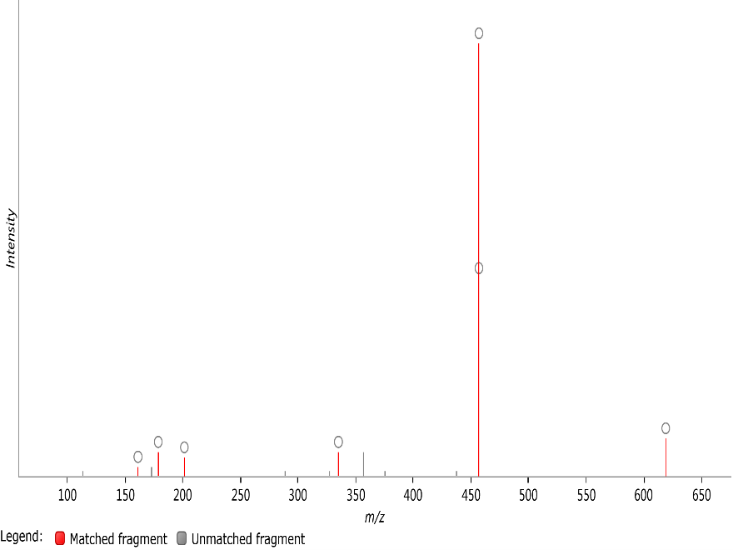  **#69** | | Guanosine, CID: 6802, METLIN; [86.8]; {CCMSLIB00005467753}  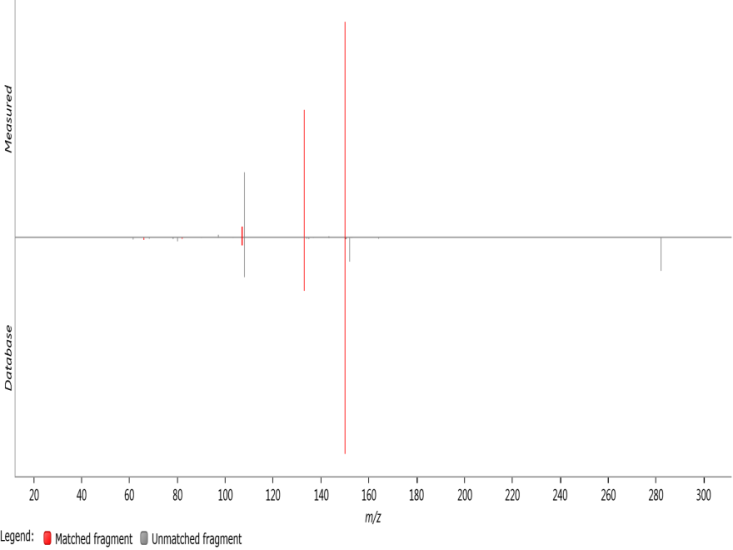  **#70** | | |
| Isoferulic acid, matched with in-house library, CID: 736186, HMDB; [53.6]  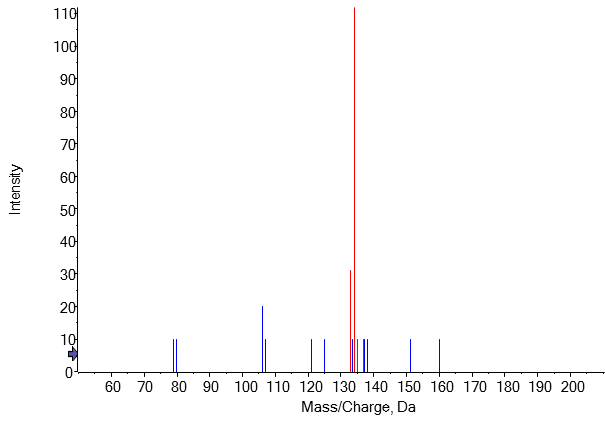  **#71** | | Isovalerylglucuronide, CID: 137383, HMDB; [38.5]; {CCMSLIB00005467703}  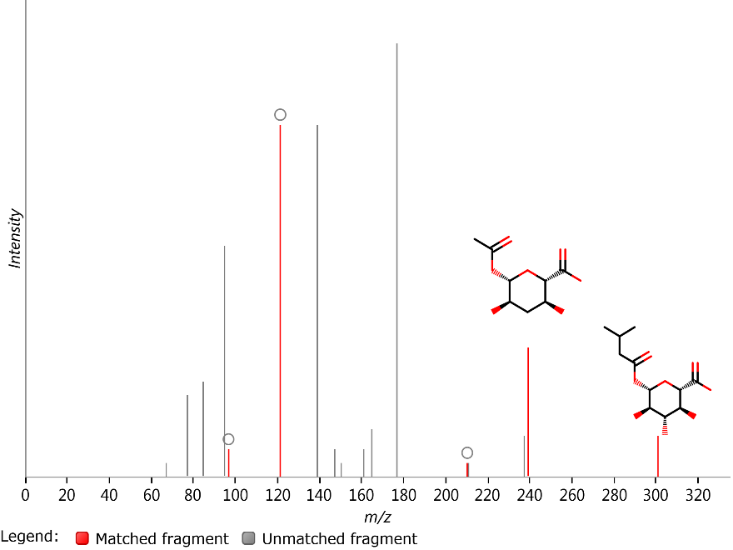  **#72** | | |
| Kuwanon Y, CID: 14334307, HMDB; [86.2]; {CCMSLIB00005467704}  **#74**  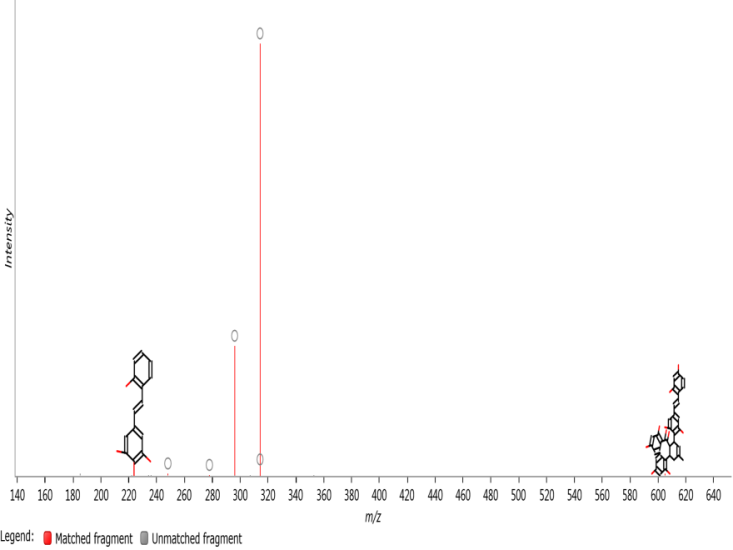 | | Kynurenic acid, CID: 3845, METLIN; [86]; {CCMSLIB00005467705}  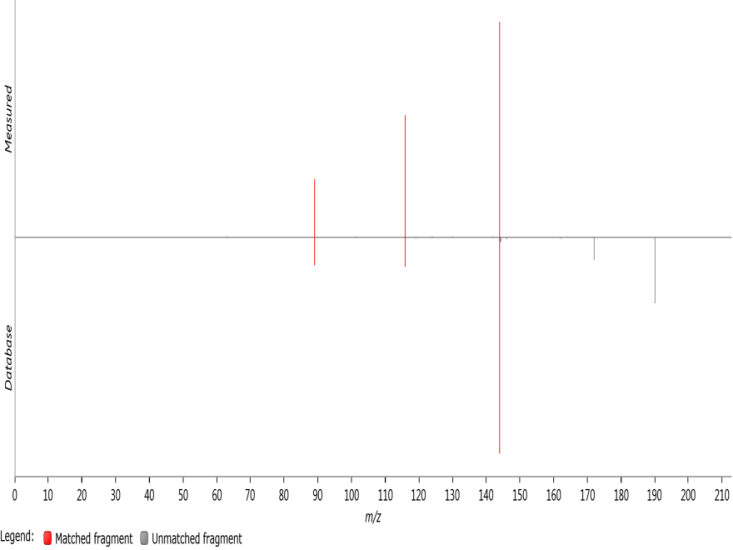  **#75** | | |
| L-Arginine, CID: 28782, METLIN; [74.8]; {CCMSLIB00005467706}  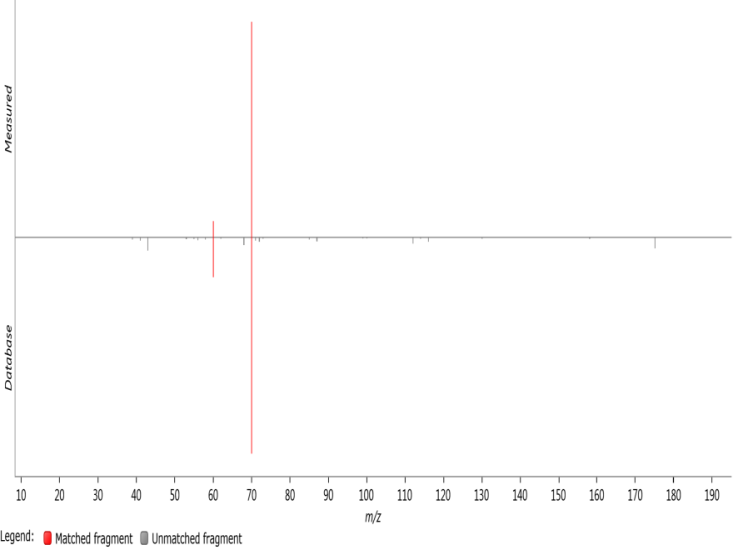  **#76** | | | | Linustatin, CID: 119301, METLIN; [59.8]; {CCMSLIB00005467707}  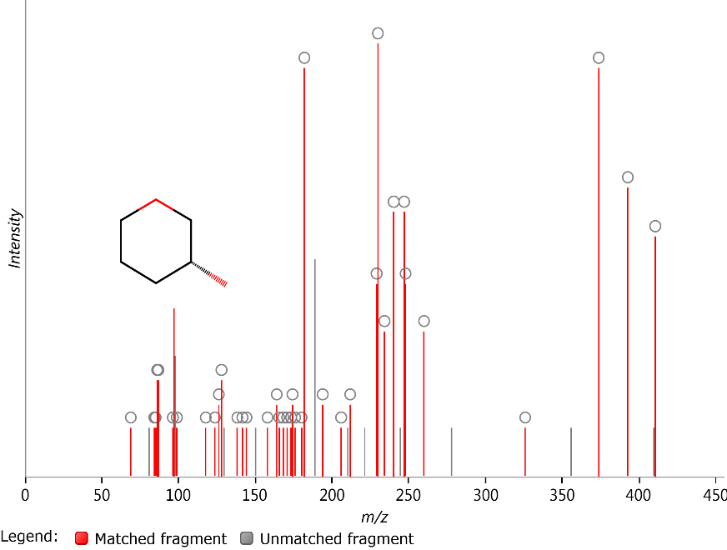  **#77** |
| Longicamphenylone, CID: 91747202, METLIN; [51.4]; {CCMSLIB00005467708}  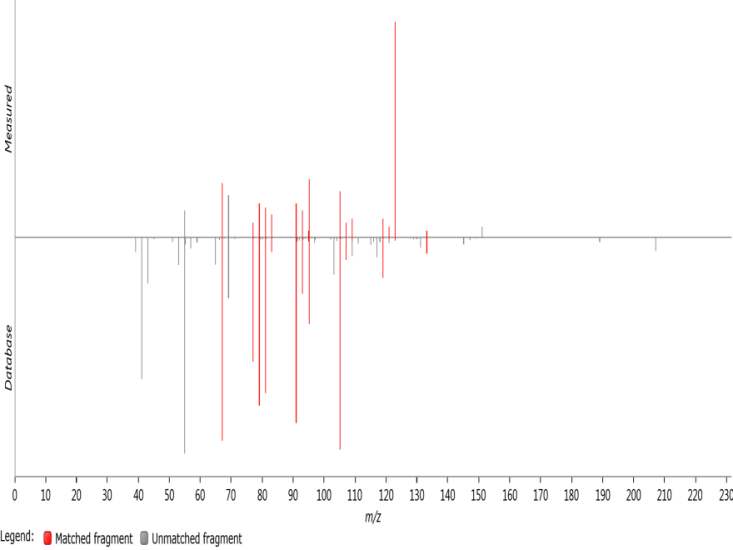  **#78** | | | | Longifolenaldehyde, CID: 565584, METLIN; [50.9]; {CCMSLIB00005467709}  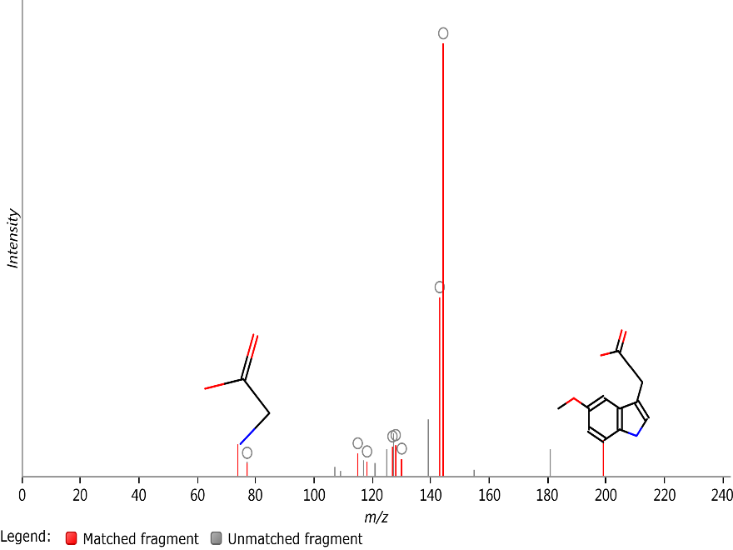  **#79** |
| L-Ribulose, CID: 644111, HMDB; [71.7]; {CCMSLIB00005467754}  **#80**  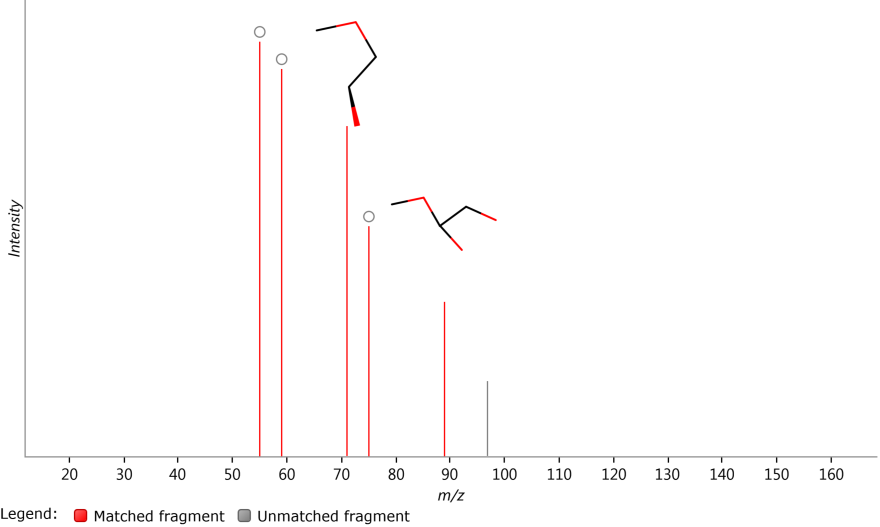 | | | | Malate, CID: 20130941, in-house library; [95.1]; {CCMSLIB00005467755}  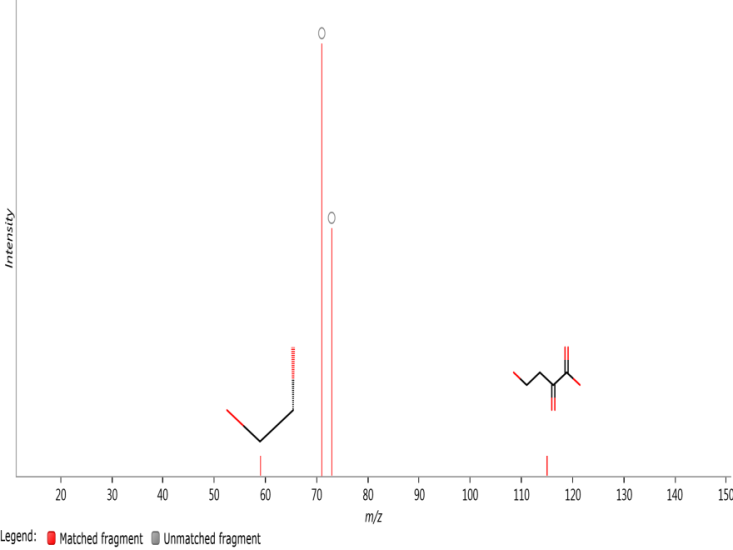  **#83** |
| Mangiferin, CID: 5281647, METLIN; [58.2]; {CCMSLIB00005467756}  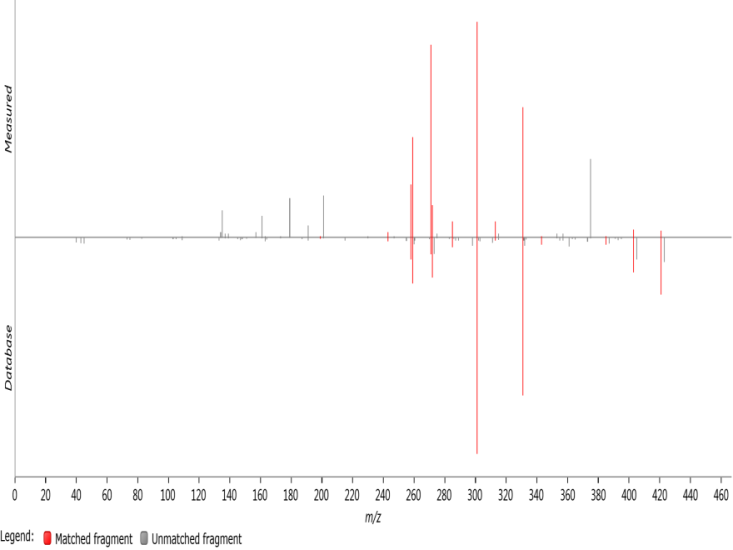  **#84** | | | | Muramic acid, CID: 433580, ChEBI; [90.7]; {CCMSLIB00005467710}  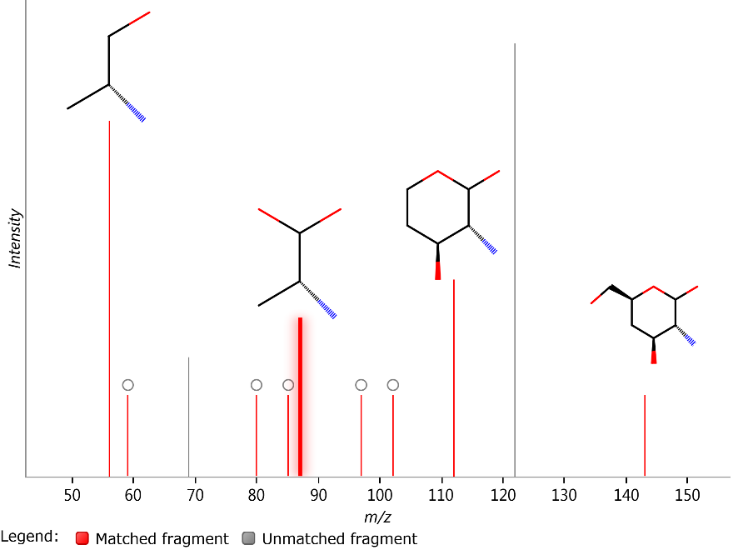  **#85** |
| N-(1-Deoxy-1-fructosyl)phenylalanine, CID: 101039148, HMDB; [52.3]; {CCMSLIB00005467711}    **#86**  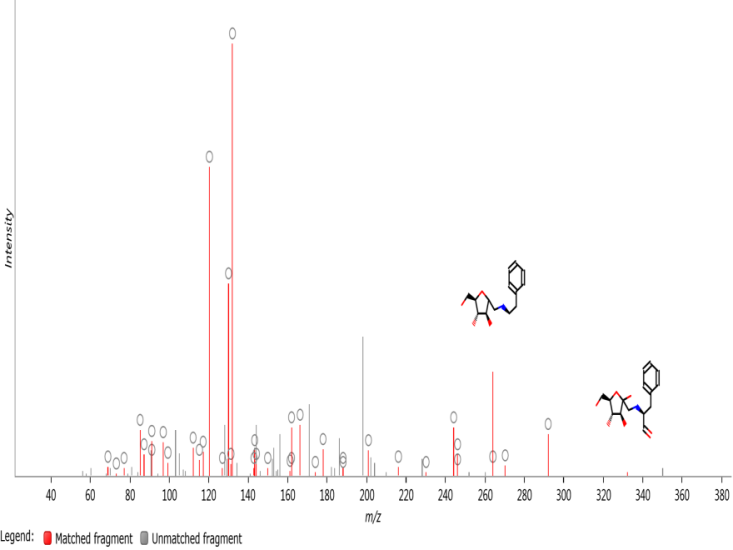 | | | | N1,N5,N10,N14-Tetra-trans-p-coumaroylspermine, CID: 9810941, METLIN; [48.9]; {CCMSLIB00005467712}  **#87**  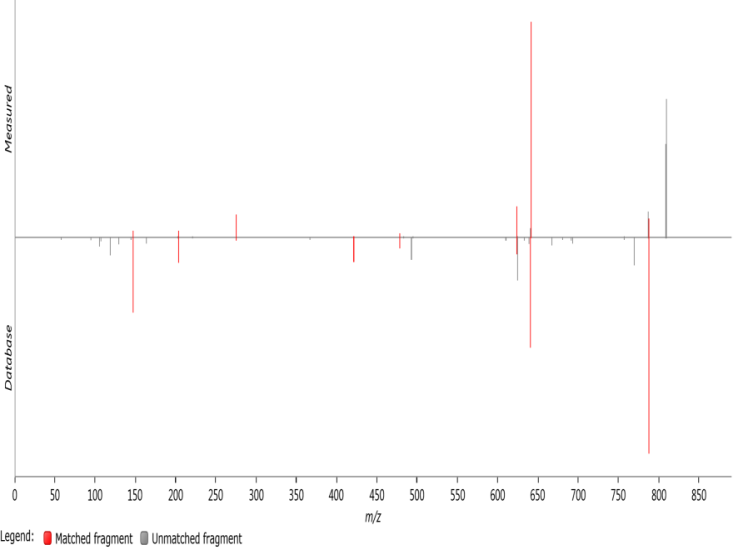 |
| N-Acetyl-D-glucosamine, CID: 899, in-house library; [58.8]; {CCMSLIB00005467713}  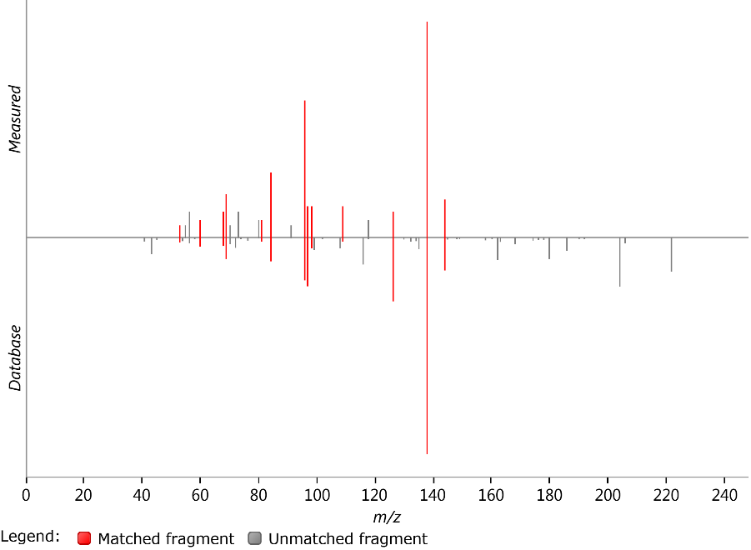  **#88** | | | | N-Acetyl-L-glutamic acid, CID: 70914, METLIN; [65]; {CCMSLIB00005467714}  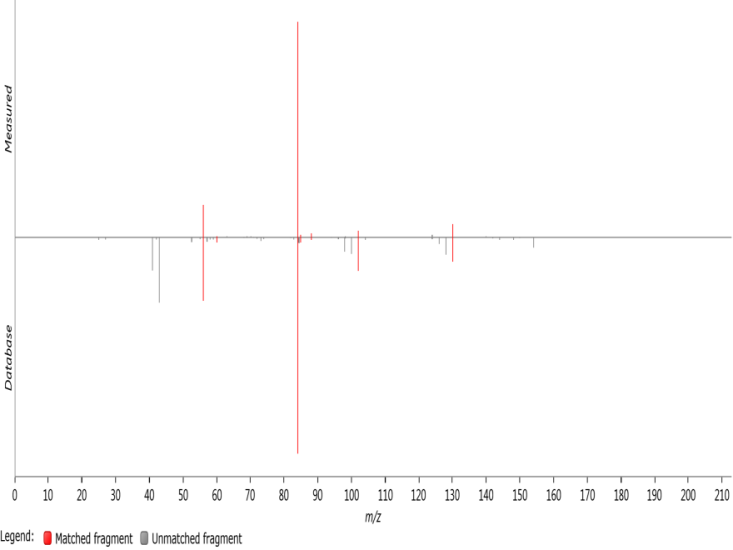  **#89** |
| Niacin (Nicotinic acid), CID: 938, METLIN; [88.8]; {CCMSLIB00005467776}  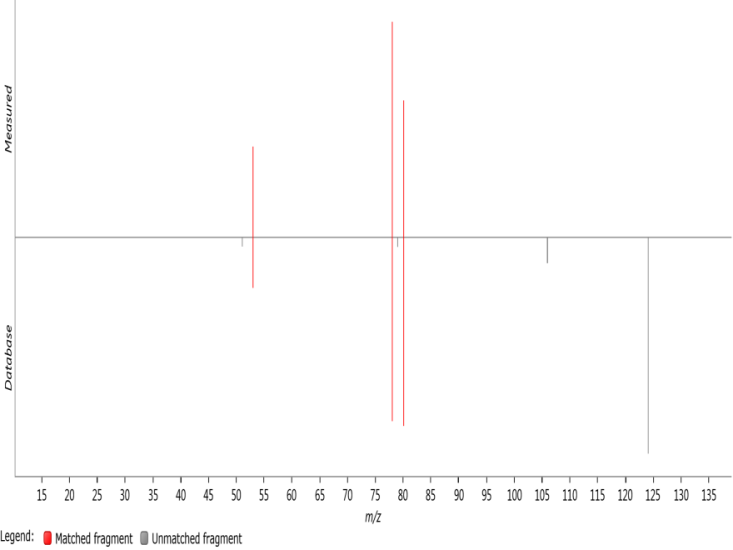  **#91** | | | | Nomilinic acid 17-glucoside, CID: 444212, HMDB; [45.5]; {CCMSLIB00005467716}  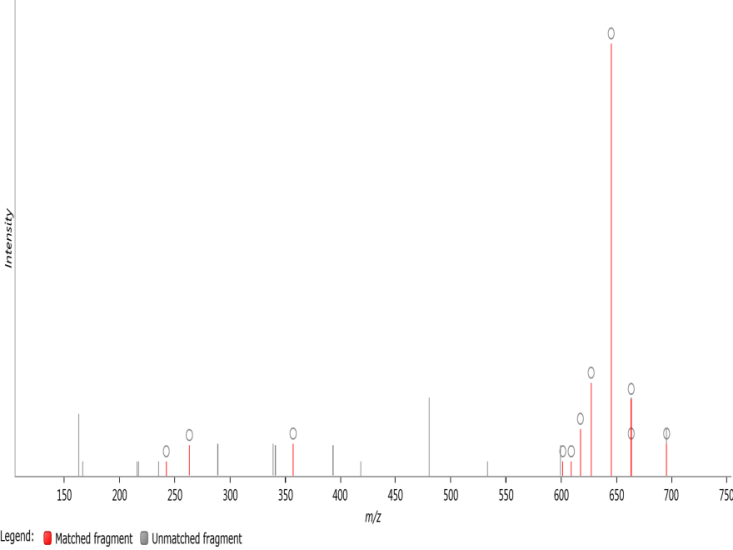  **#92** |
| Palmitic acid, matched with in-house library, CID: 985, HMDB; [26.3]  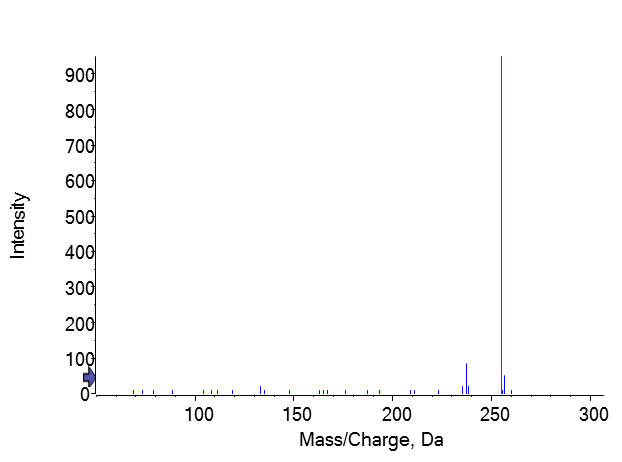  **#93** | | | | Pelargonidin 3-O-glucoside, CID: 443648, METLIN; [54.8]; {CCMSLIB00005467757}  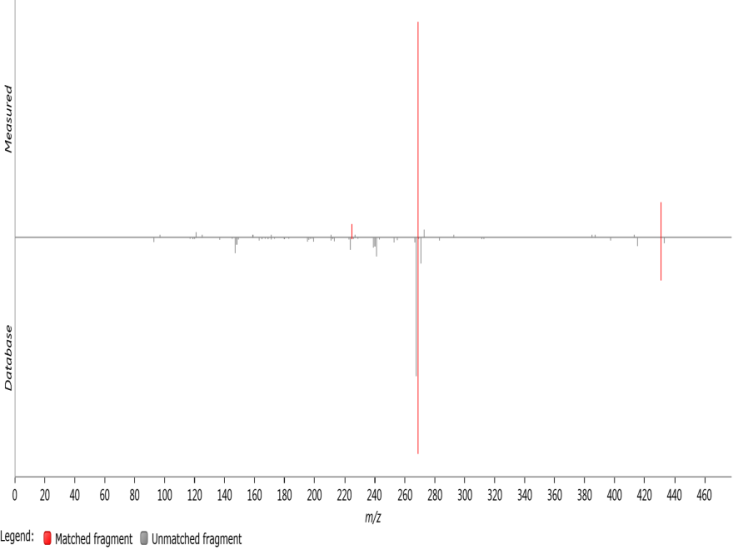  **#95** |
| Phlorin, CID: 476785, METLIN; [37.3]; {CCMSLIB00005467718}  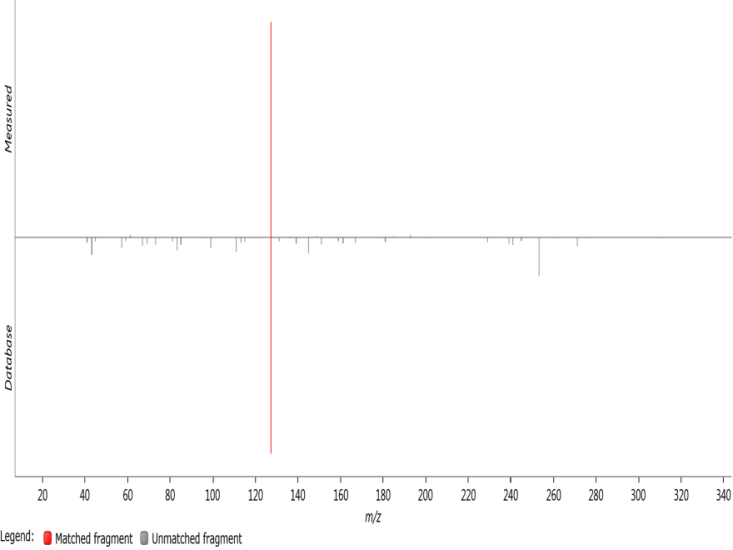  **#96** | | | | Purgic acid B, CID: 16091605, KNApSAcK; [33.4]; {CCMSLIB00005467775}  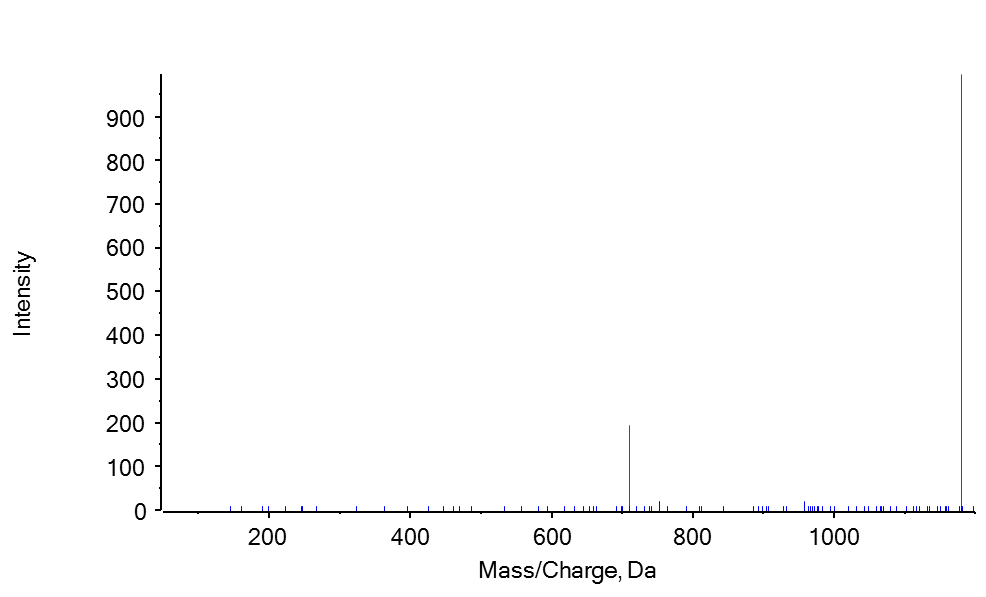  **#98** |

| Quercetin 3-(6'-acetylglucoside), CID: 44259187, METLIN; [59.7]; {CCMSLIB00005467759}  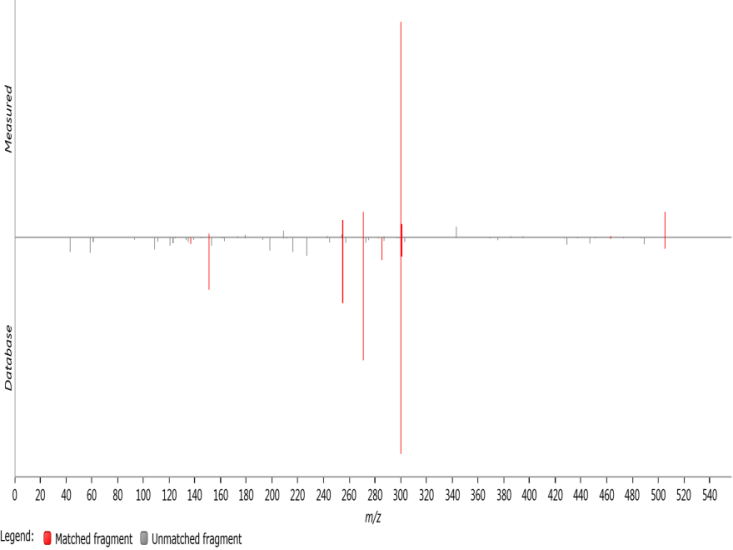  **#100** | Quercetin 3-O-glucoside, CID: 5280804, METLIN; [62.5]; {CCMSLIB00005467759}  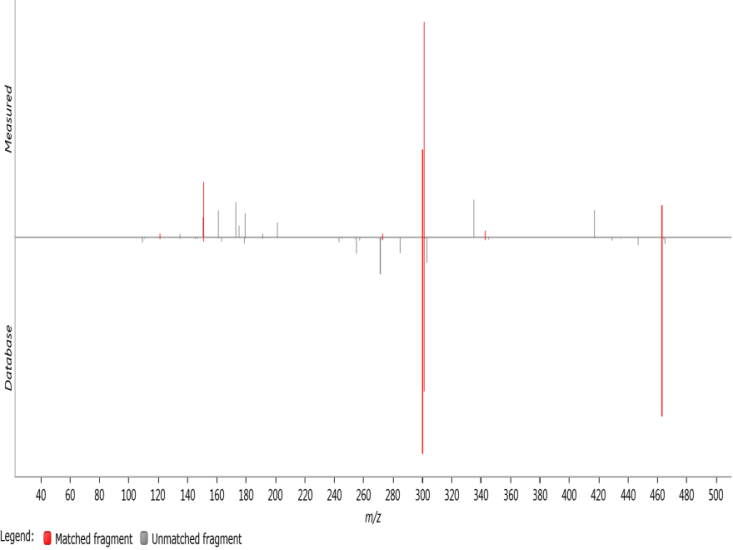  **#101** |
| --- | --- |
| Sambacin, CID: 131752486, HMDB; [34.1]; {CCMSLIB00005467720}  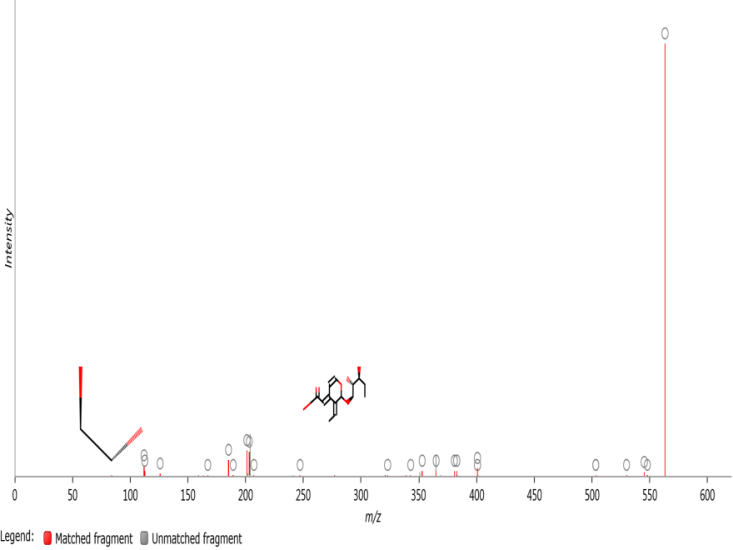  **#103** | Shanzhiside, CID: 11948668, ChEBI; [81.3]  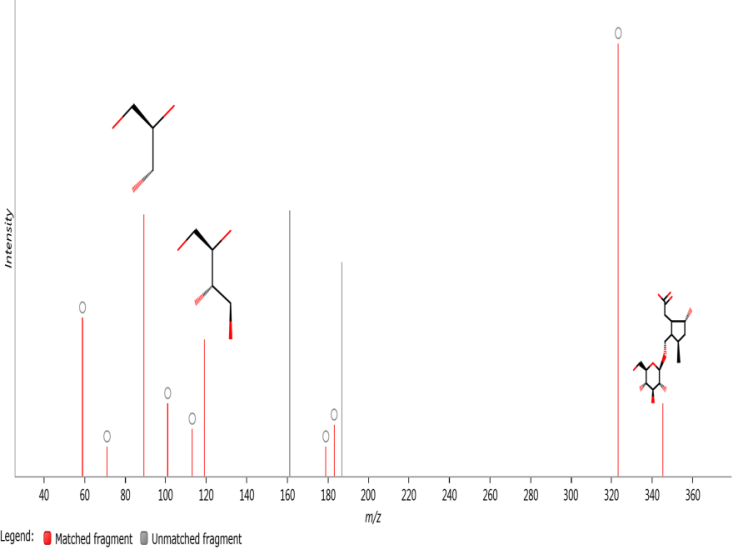  **#104** |
| Soyacerebroside, CID: 131751281, HMDB; [37.7]; {CCMSLIB00005467760}  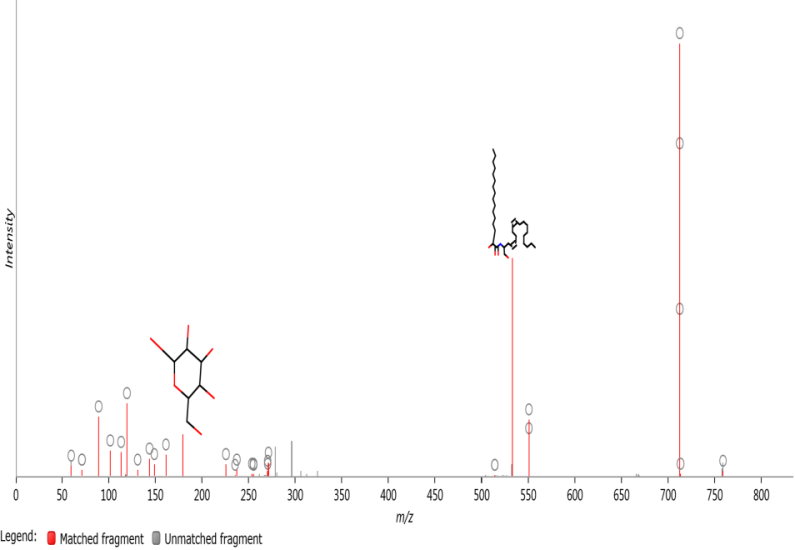  **#105** | Stachyose, CID: 439531, METLIN; [68.6]; {CCMSLIB00005467721}  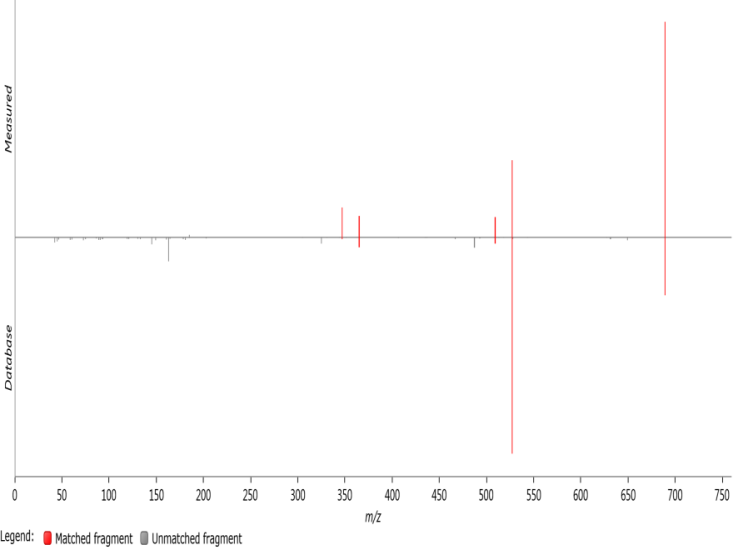  **#106** |
| Succinate, matched with in-house library, CID: 1110, HMDB; [55.0]; {CCMSLIB00005467761}  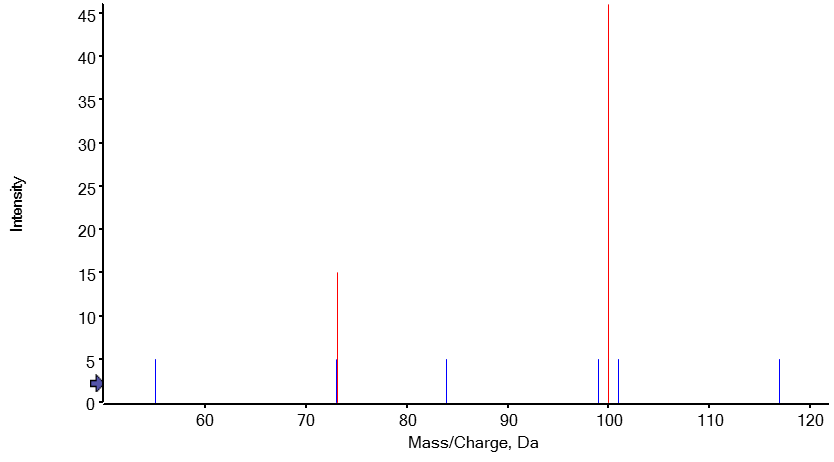  **#107** | Succinoadenosine, CID: 126969142, METLIN; [57.0]; {CCMSLIB00005467722}  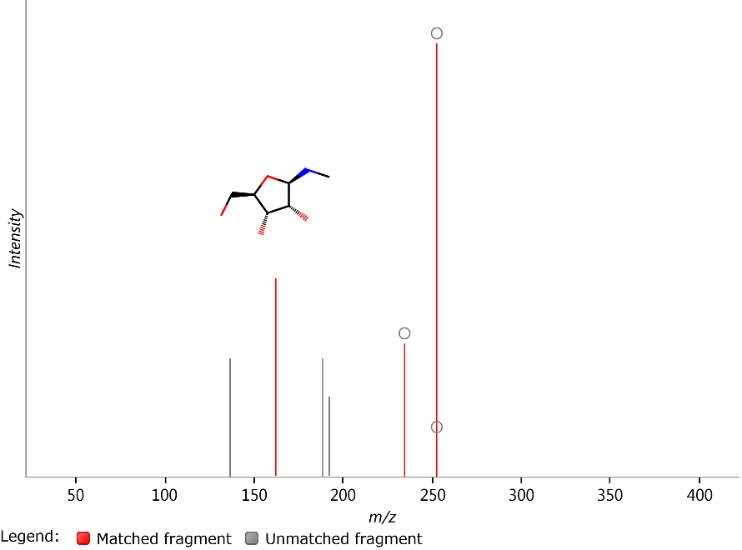  **#108** |
| Succinyl-L-proline, CID: 194156, ChEBI; [67.6]; {CCMSLIB00005467723}  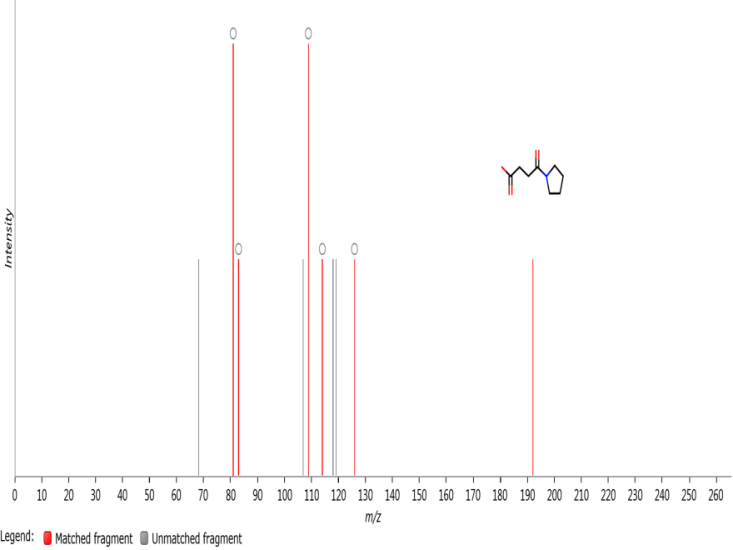  **#109** | Swertiamarin, CID: 442435, ChEBI; [91.9]; {CCMSLIB00005467762}  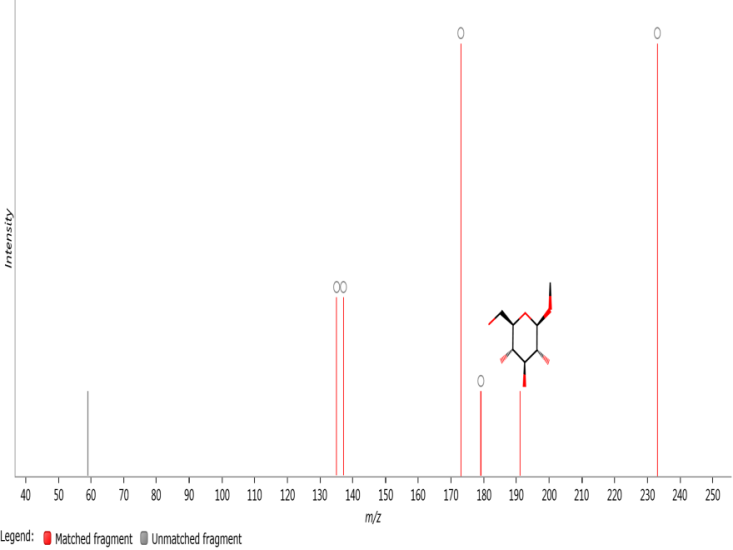  **#110** |
| Tetradecanedioic acid, CID: 13185, METLIN; [71.7]; {CCMSLIB00005467764}  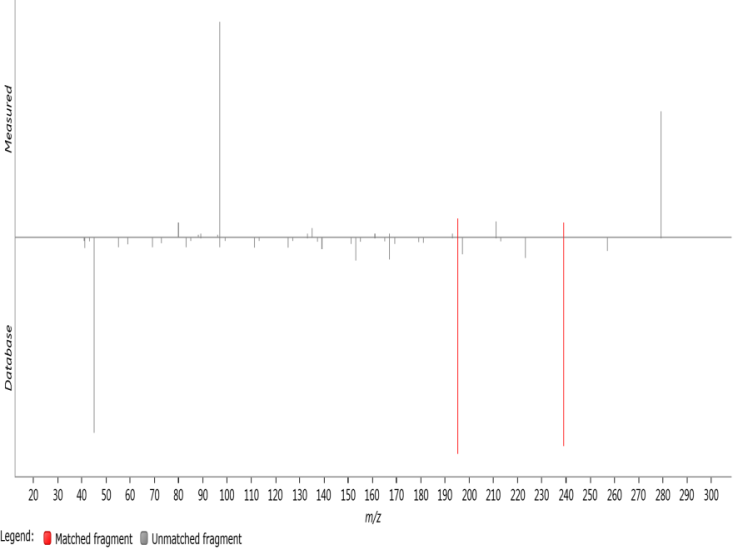  **#111** | Traumatic Acid, CID: 5283028, METLIN; [77.4]; {CCMSLIB00005467765}  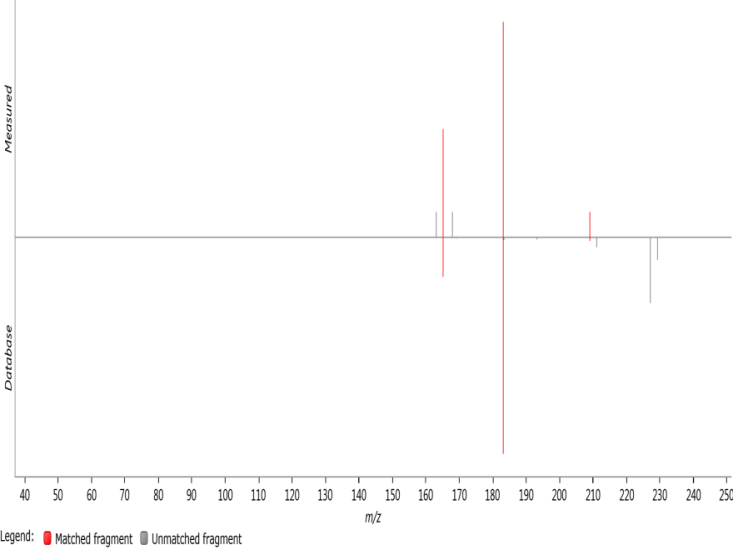  **#112** |
| Tropic acid, CID: 10726, METLIN; [69.0]; {CCMSLIB00005467769}  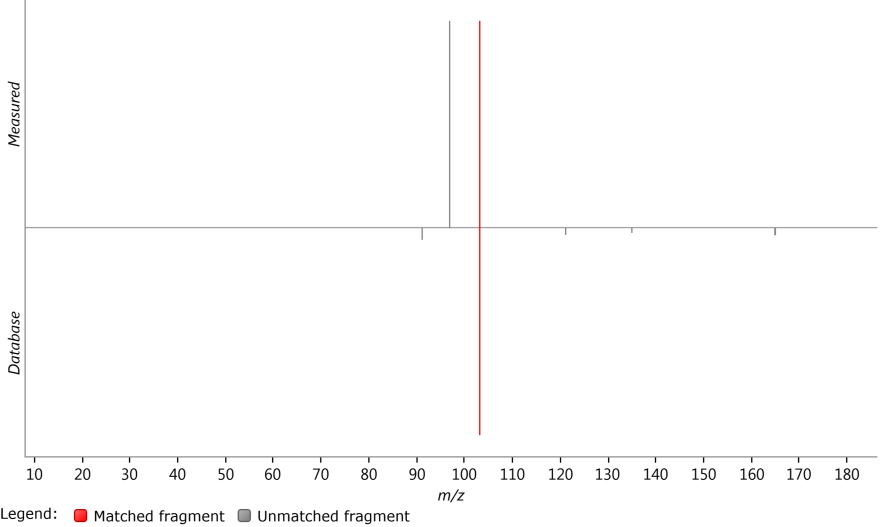  **#113** | Tsangane L 3-glucoside, CID: 73981648, HMDB; [41.6]; {CCMSLIB00005467770}  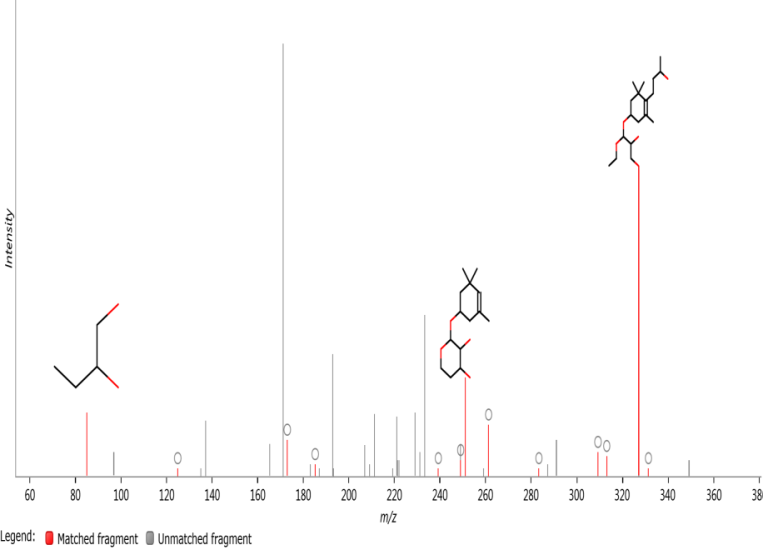  **#114** |
| Uric acid, CID: 1175, METLIN; [56.4]; {CCMSLIB00005467724}  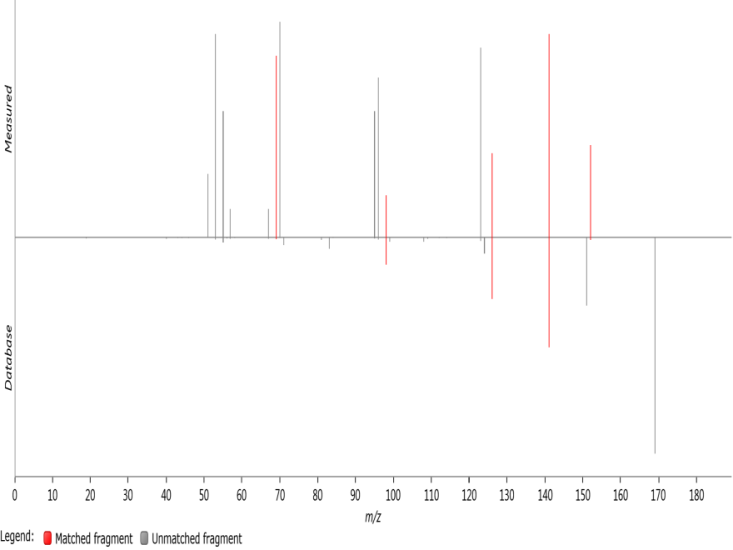  **#115** | Vincosamide, CID: 10163855, ChEBI; [79.9]; {CCMSLIB00005467725}  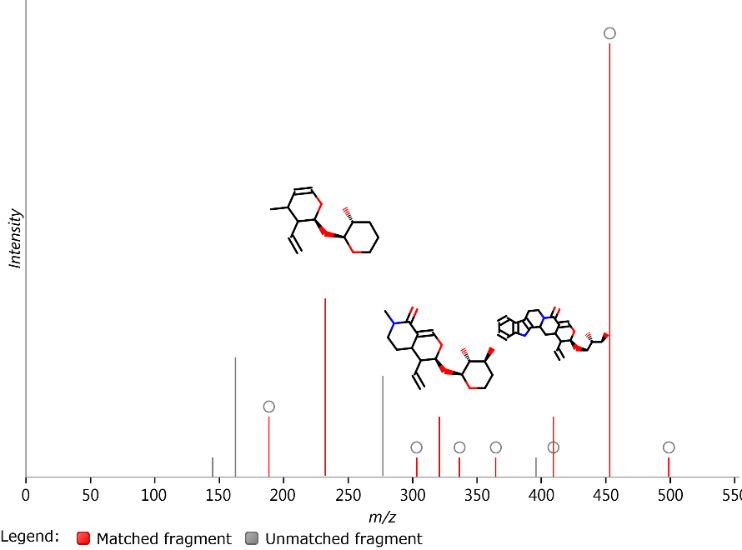  **#116** |
| Xanthurenic acid, CID: 5699, METLIN; [76.6]; {CCMSLIB00005467726}  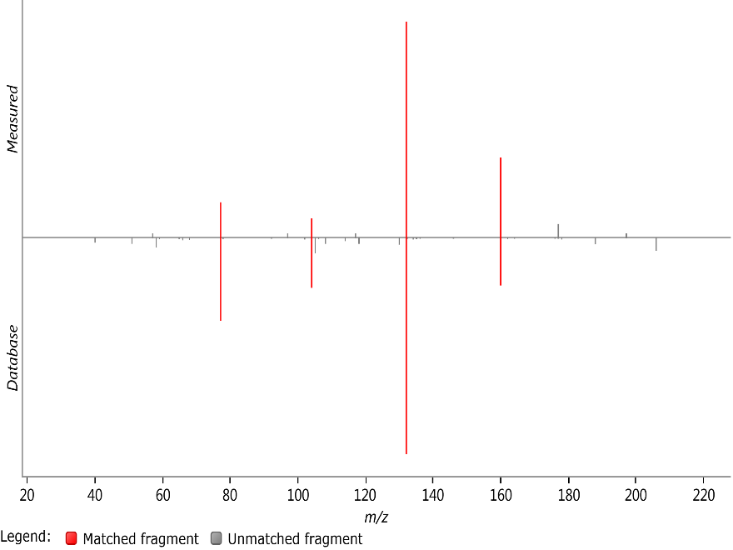  **#117** |  |

**Figure S7b.** MS/MS spectra for compounds present in CA water extracts that were identified using authentic standards (L1 annotations). MS/MS score is indicated in [ ].

| 5-O-Caffeoylquinic acid; [81.8]  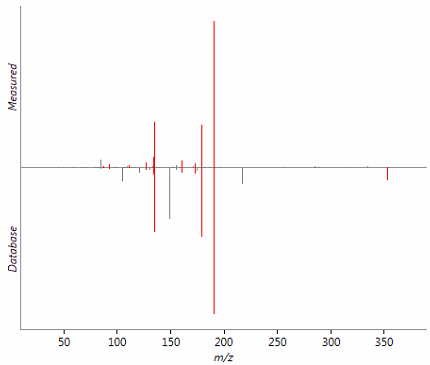 | 3-O-Caffeoylquinic acid; [87.9]  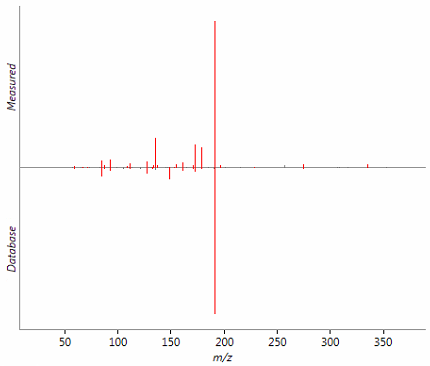 |
| --- | --- |
| 4-O-Caffeoylquinic acid; [79.9]  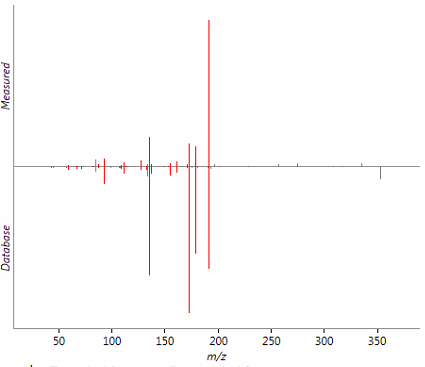 | 1,5-Dicaffeoylquinic acid; [92.8]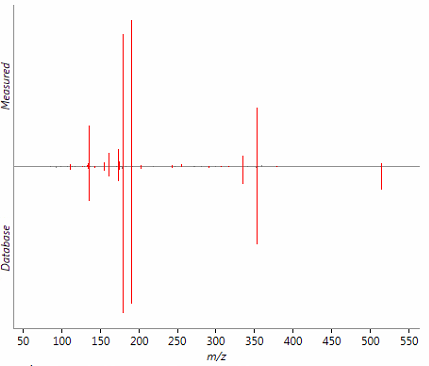 |
| 1,3-Dicaffeoylquinic acid; [85.7]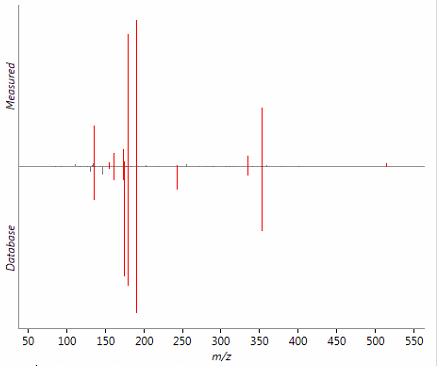 | 3,4-Dicaffeoylquinic acid; [91.9]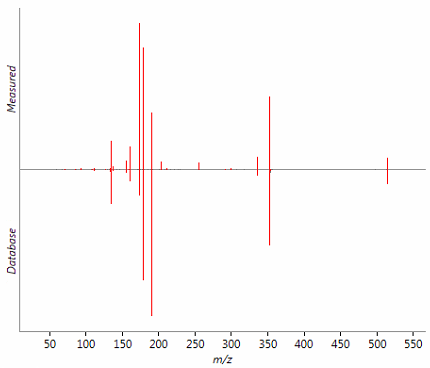 |
| 3,5-Dicaffeoylquinic acid; [93.2]  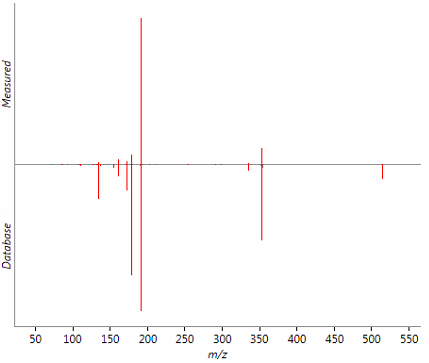 | 4,5-Dicaffeoylquinic acid; [91.2]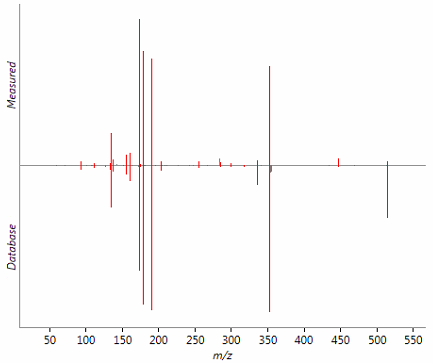 |
| Quercetin; [76.1] | Kaempferol; [92.6] |
| Caffeic acid; [98.2] | Ferulic acid; [48.4] |
| Rutin; [45.4] | Madecassoside; [97.7] |
| Asiaticoside; [86.7] | Madecassic Acid; [47.3] |
| Asiatic acid; [54.7] |  |

**Appendix 1-** Recovery of marker compounds using different sonication time.

**Appendix 1.** Recovery of marker compounds using different sonication times. Injection of 10 µl of *C. asiatica* water extract (0.1 mg/ml). Sonication was for 15 min (pink line) and 30 min (blue line). 30 min increases the recovery of less hydrophilic compounds such as di-caffeoylquinic acids with no degradation of mono-caffeoylquinic acids.
